# Adaptive Antioxidant Nanomedicines Inhibit Ferroptosis in Renal Tubular Epithelial Cells to Alleviate Diabetic Kidney Disease

**DOI:** 10.1002/advs.202505168

**Published:** 2025-07-06

**Authors:** Zerun Liu, Ting Huang, Ying Hong, Yuqi Yang, Wensheng Chen, Qingtao Zeng, Qiaohui Chen, Yongqi Yang, Xiaohong Ying, Wan Zeng, Ziyu Wu, Tianjiao Zhao, Xuesi Wan, Jianlin Chen, Kelong Ai, Qiong Huang

**Affiliations:** ^1^ Department of Pharmacy Xiangya Hospital Central South University Changsha 410008 China; ^2^ National Clinical Research Center for Geriatric Disorders Xiangya Hospital Central South University Changsha 410008 China; ^3^ Xiangya School of Pharmaceutical Sciences Central South University Changsha 410078 China; ^4^ Hunan Provincial Key Laboratory of Cardiovascular Research Xiangya School of Pharmaceutical Sciences Central South University Changsha 410013 China; ^5^ Department of Endocrinology and Diabetes Center The First Affiliated Hospital of Sun Yat‐sen University Guangzhou Guangdong China; ^6^ Pancreatic Surgery Xiangya Hospital Central South University Changsha 410008 China; ^7^ Key Laboratory of Aging‐related Bone and Joint Diseases Prevention and Treatment Ministry of Education Xiangya Hospital Central South University Changsha 410008 China

**Keywords:** adaptive antioxidant nanodrug, diabetic kidney disease, ferroptosis, GPX4, mtROS

## Abstract

Diabetic kidney disease (DKD) imposes a heavy medical burden worldwide due to the lack of effective treatment. High levels of mtROS and mitochondrial damage in the renal tubules are the initiating and core factors driving the progression of DKD. However, the effectiveness of current antioxidant drugs is greatly limited, mainly due to the difficulty of simultaneously breaching the glomerular barrier and targeting tubular mitochondria, as well as their limited ability to sustain treatment of chronic DKD. Here, this study reports a Se embedded adaptive antioxidant nanodrug (AAN) with negative surface charge and high mitochondrial targeting that can pass through the renal tubules and be highly enriched in the affected renal tubular mitochondria in DKD. AAN can eliminate mtROS to release soluble Se, which is then converted into the key bioactive enzymes ‐GPX4, effectively inhibiting ferroptosis and protecting mitochondria by exerting adaptive antioxidant effects. In the DKD mouse model, AAN treatment can effectively restore renal function, and the therapeutic effect at a dose of 10 mg kg^−1^ every 4 days is significantly better than Metformin administered at a dose of 200 mg kg^−1^ per day. In conclusion, this study provides a promising strategy to enhance the effects of antioxidant therapy to break the pathological barriers in DKD treatment.

## Graphical abstract

In this study, an adaptive antioxidant nanodrug (AAN) is developed through the self‐polymerization of L‐selenocysteine. The AAN exhibits dual functionalities: antioxidant activity (scavenging reactive oxygen species) and mitochondrial targeting. Moreover, the released selenium (Se) participates in the synthesis of glutathione peroxidase 4 (GPX4), which further eliminates ROS (Figures A & B). The AAN, characterized by its ultrasmall particle size, efficiently enters renal tubules and targets mitochondria to scavenge ROS. Subsequently, the released Se is utilized for endogenous GPX4 synthesis, which further eliminates ROS and inhibits lipid peroxidation, thereby alleviating ferroptosis. Given that diabetic kidney disease (DKD) is accompanied by inflammation and fibrosis, the AAN concurrently mitigates these pathological processes, demonstrating synergistic interactions with ferroptosis inhibition.

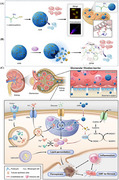

## 1 Introduction

The number of diabetic patients has increased significantly worldwide, exceeding 800 million. Diabetes can cause a series of microvascular complications, among which DKD has attracted much attention due to its high incidence (accounting for 20–40% of diabetic patients).^[^ ^1^ ^]^ Currently, treatments for DKD are limited to controlling blood pressure with renin‐angiotensin‐aldosterone system inhibitors and controlling blood glucose with Metformin and sodium‐glucose cotransporter 2 inhibitors.^[^ ^2^, ^3^ ^]^ Although these methods have certain renal protective effects, the therapeutic effects are unsatisfactory, and it is difficult to inhibit the progression of DKD to end stage renal disease (ESRD) through these methods.^[^ ^4^ ^]^ Due to the lack of effective intervention measures, the 10‐year mortality rate of DKD is as high as 31.1%.^[^ ^5^ ^]^ In fact, the risk of all‐cause mortality from the comorbidity of diabetes and chronic kidney disease is even higher than that from myocardial infarction.^[^ ^6^ ^]^ The large and growing DKD patient population has caused huge social and medical expenditures, and effective treatments to alleviate DKD are an extremely desired and far‐from‐met medical need.^[^ ^7^ ^]^

Emerging evidence suggests that glomerular injury in DKD is neither a primary event in early renal damage nor a determinant of DKD progression. Accordingly, tubular injury occurs earlier than glomerular injury and is more critical in DKD.^[^ ^8^ ^]^ Alterations in mitochondrial dynamics in proximal tubule epithelial cells (PTECs) have been observed before the onset of proteinuria excretion and renal histological defects in DKD, suggesting that mitochondrial dysfunction in PTECs is an initiating factor in DKD.^[^ ^9^ ^]^ The kidney is an organ that is highly rich in mitochondria, second only to the heart. Reabsorption is the main function of the proximal tubule, and this active reabsorption process consumes a lot of energy, which leads to the mitochondrial density in PTECs far exceeding that of other kidney cells. In the early stage of DKD, multiple factors such as high glucose led to a substantial increase in mtROS, inducing mitochondrial dysfunction and oxidative stress in PTECs, thus causing renal tissue damage and renal function loss.^[^ ^10^ ^]^ Moreover, PTECs are prone to iron accumulation and lipid peroxidation in DKD, due to chronic high glucose and oxidative stress, which in turn initiates ferroptosis to induce inflammatory cell infiltration, causes renal fibrosis, and ultimately leads to ESRD.^[^ ^11^ ^]^ Therefore, high levels of mtROS and subsequent dysfunctional mitochondria in PTECs are at the pathological core of tubulopathy in DKD, especially in early renal injury. Despite this, no antioxidants are currently approved for clinical use in the treatment of DKD. The root cause is that common antioxidants often lack mitochondrial targeting and renal tubular targeting in vivo. Modification with mitochondrial targeting groups can endow antioxidants with certain mitochondrial targeting capabilities. However, currently common mitochondrial targeting groups such as triphenyl phosphonium are usually highly positively charged.^[^ ^12^ ^]^ Antioxidants modified with mitochondrial targeting groups preferentially accumulate in the liver or lungs rather than the kidneys in vivo, thereby significantly reducing bioavailability and increasing toxic side effects.^[^ ^13^ ^]^ Moreover, the ROS level in PTECs is a steady‐state process. ROS is also an essential biological signaling molecule, and excessive removal of ROS can lead to cell dysfunction.^[^ ^14^, ^15^ ^]^ Since DKD is a chronic disease, long‐term or repeated administration of antioxidants is required, causing systemic‐ROS fluctuations, leading to side effects and reduced therapeutic efficacy due to their‐toxicity and nonspecific distribution.^[^ ^16^ ^]^ There is an urgent need for a negatively charged antioxidant nanodrug that can target mitochondria and adaptively eliminate mtROS to restore mitochondrial function in PTECs.

**Scheme 1:**
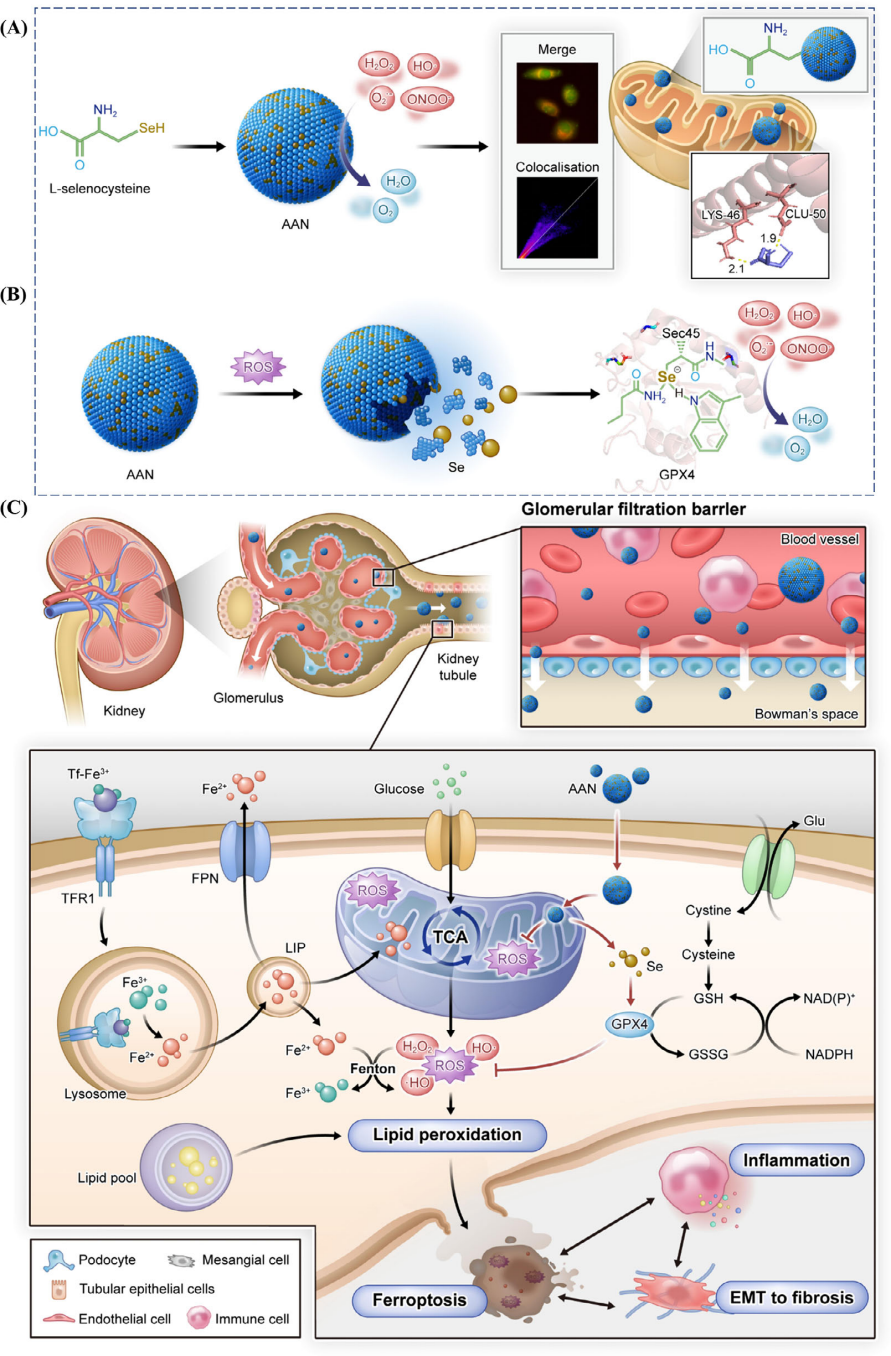
Synthesis of AAN, mitochondrial targeting, and DKD genesis. A) In vitro antioxidation and mitochondrial targeting of AAN. B) AAN is slowly released and becomes the raw material for GPX4 synthesis. C) AAN enters renal tubules with its very small particle size, targets mitochondria to eliminate ROS, and then releases Se to synthesize GPX4 in vivo, which further eliminates ROS, inhibits lipid peroxidation, and thus alleviates ferroptosis. DKD is accompanied by inflammation and fibrosis, which interacts with ferroptosis, AAN can also alleviate the occurrence of inflammation and fibrosis at the same time.

Here, we developed a novel adaptive antioxidant strategy to efficiently treat DKD via a highly mitochondrial‐targeted adaptive antioxidant nanodrug (AAN). AAN is a selenium‐doped carbon composite nanoparticle formed by temperate thermal decomposition of selenocysteine ​​under alkaline conditions. AAN has the ability to efficiently eliminate various ROS due to its abundant Se and phenolic hydroxyl groups. Moreover, AAN inherits the aminoacetic acid group from selenocysteine, ensuring that AAN is not only negatively charged but also highly mitochondrial targeted. In previous studies, positively charged groups were frequently employed for mitochondria targeting, primarily due to the abundance of negatively charged phospholipids on the mitochondrial membrane. However, in addition to phospholipids, the mitochondrial membrane contains an exceptionally riched mitochondrial membrane proteins, which provides a potential targeting strategy using negatively charged groups.^[^ ^13^, ^17^ ^]^ In DKD mouse, intravenously injected AAN was highly enriched in the kidneys, and could pass through the glomerular barrier to reach PTECs due to its ultra‐small size. AAN is then highly enriched in mitochondria of PTECs, eliminates excessive mtROS and releases soluble selenium, which is efficiently converted into GPX4 by PTECs. As a key antioxidant enzyme, GPX4 automatically adapts to changes in the redox environment, accurately regulates the redox balance in PTECs, inhibits iron overload, reduces lipid peroxidation, and thereby protects PTECs from ferroptosis.^[^ ^9^, ^18^, ^19^ ^]^ Thanks to this highly efficient adaptive antioxidant strategy, AAN significantly improved the renal function of DKD mice, reduced renal damage and inhibited the process of fibrosis, with the effect being significantly better than Metformin, the first‐line drug for type 2 diabetes. This innovative therapeutic strategy provides an effective treatment for DKD and opens up new directions for the research and clinical application of targeted cell death mechanisms (**Scheme** **1**).

## 2 Results

### 2.1 Characterization of AAN

**Figure 1:**
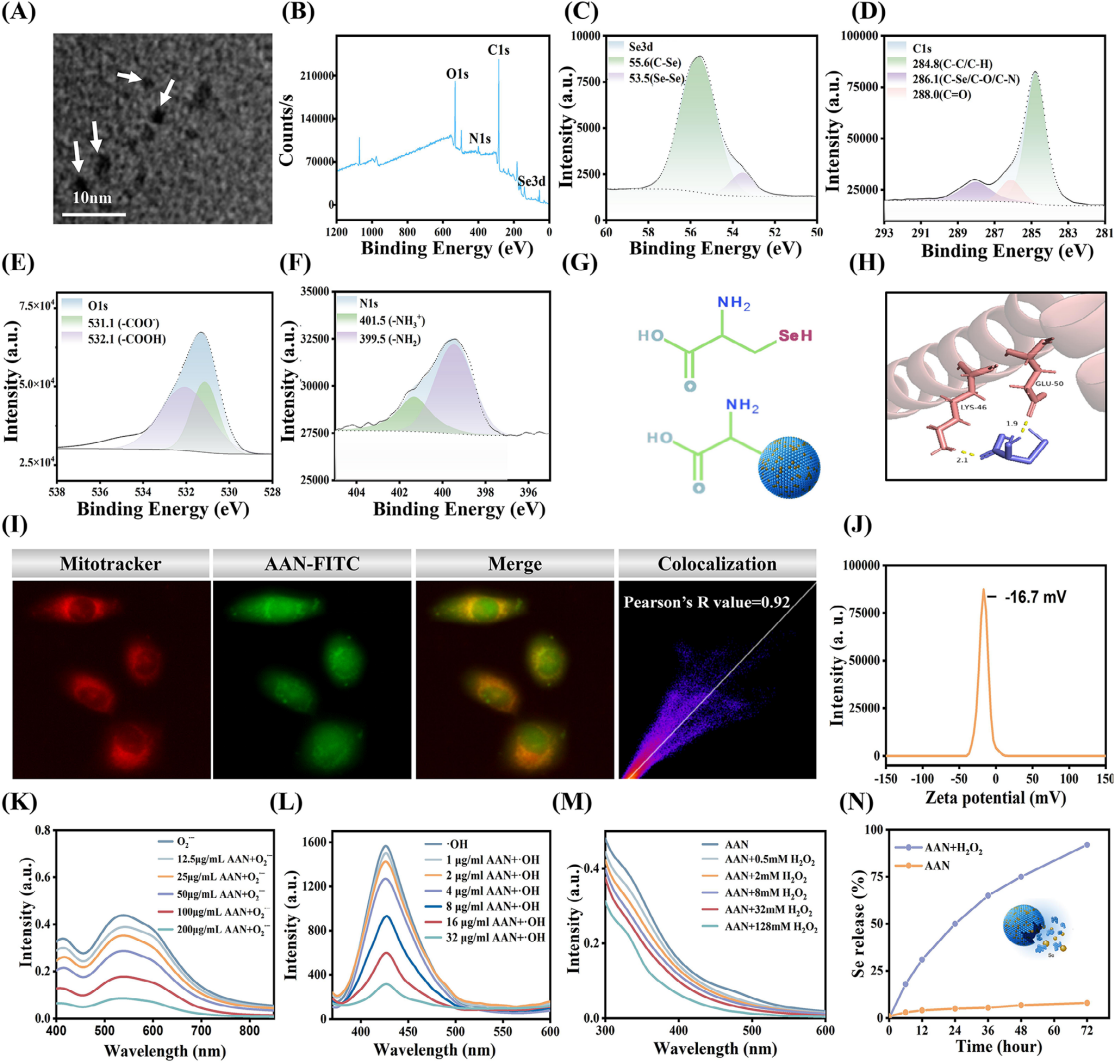
Synthesis and characterization of AAN. A) TEM image of AAN, Scale bar: 10 nm. B‐F) XPS spectrum of AAN (B), Se3d(C), C1s(D), O1s(E), N1s(F) in AAN. G) Schematic diagram of the inheritance of the selenocysteine‐derived aminoacetic acid group by AAN. H) AAN docks with the mitochondrial outer membrane protein TOM20 molecule. I) Mitotracker‐stained with AAN‐FITC colocalization and Pearson coefficient in HK‐2 cells, EX/EM = 579/599nm. J) Zeta potential distribution intensity of AAN. K‐L) Elimination of O_2_^∙−^(K), ∙OH (L) by different concentrations of AAN. M) The ability of AAN to eliminate different concentrations of H_2_O_2_. N) Release degree of Se in AAN with or without H_2_O_2_.

AAN was prepared by polymerization of L‐selenocysteine leading to partial carbonization under alkaline conditions at an appropriate temperature (60 °C). At this temperature, a high proportion of Se can be embedded into the carbon skeleton of AAN and also enable AAN to inherit the aminoacetic acid group from L‐selenocysteine. Through transmission electron microscopy (TEM), AAN is a monodisperse nanodot with a diameter of about 5–10 nm (**Figure** **1**A). Through X‐ray photoelectron (XPS), the main components of AAN are C, N, O, and Se, among which the Se content reaches 10.37% (Figure 1B). At the same time, STEM‐EDS mapping provided direct visualization of the spatial distribution of carbon, nitrogen, oxygen, and selenium. Selenium was found to be distributed relatively uniformly across the carbon platform (Figure S1, Supporting Information). Through Se3d XPS fine spectrum, the Se peak at 55.6 eV is completely attributed to the C‐Se bond in AAN, indicating that Se is embedded in the carbon skeleton through covalent bonds (Figure 1C). Strong covalent bonds linking Se can ensure the stability of Se in AAN under physiological conditions. The XPS fine peaks of C in AAN were further deconvoluted into three different peaks at 284.8 eV, 286.1 eV, and 288.0 eV, which were attributed to C‐C, C‐Se/C‐N/C‐O, and C = O bonds, respectively (Figure 1D). Further, the peaks at 531.1 eV and 532.1 eV in the XPS fine spectrum of O1s indicate that AAN has abundant carbonyl and carboxyl groups (Figure 1E). Finally, the N1s XPS fine spectrum in AAN showed the primary amino peaks at 399.5 eV and 401.5 eV (Figure 1F). Considering that AAN is prepared from the only raw material – selenocystein and combined with the above XPS evidence, AAN contains C‐C bonds, carboxyl groups, and amino groups, indicating that AAN inherits the aminoacetic acid group derived from selenocysteine (Figure 1G). To further demonstrate the existence of amino and carboxyl groups, we used Fourier transform infrared spectroscopy (FTIR) to detect characteristic peak functional groups, and the results confirmed that the AAN structure contains amino and carboxyl groups (Figure S2, Supporting Information). The X‐ray diffraction (XRD) results show that the XRD diffraction peak is located at 24°, indicating that AAN has a typical graphite sheet structure (Figure S3, Supporting Information). Through molecular docking, the aminoacetic acid group in selenocysteine has a strong binding force with TOM20 (the most abundant outer membrane protein in mitochondria), and the binding energy can reach −2.13 kcal mol^−1^ (Figure 1H). In addition to TOM20, we also tested its binding energies with TOM34, TOM40, TOM70, VADC1, and VADC2, as shown in Figure S4 (Supporting Information). The binding energies were −4.2, −3.83, −4.98, −4.62, and −4.04 kcal mol^−1^, respectively, indicating good binding ability and suggesting its excellent targeting ability towards mitochondria. Subsequently, AAN targeting of mitochondria in human kidney 2 (HK‐2) cells was further explored. FITC‐labeled AAN (Figure S5, Supporting Information) and mitochondrial indicator Mitotracker red were co‐incubated with HK‐2 cells. As shown in Figure I, AAN can be highly enriched in the mitochondria of cells, with a Pearson coefficient as high as 0.92, which indicates that AAN has a high mitochondrial targeting function because AAN inherits the aminoacetic acid group from selenocysteine. The Pearson coefficient, as a statistic for quantifying colocalization, takes values between 1 and ‐1. A value of 1 indicates a perfect linear correlation between the fluorescence intensities of two images, while a value of ‐1 indicates a perfect but inverse correlation between the fluorescence intensities of two images.^[^ ^20^ ^]^ In addition, we co‐incubated FITC labeled L‐selenocysteine with mitochondrial indicator Mitotracker red in HK‐2 cells. Figure S6 (Supporting Information) shows that L‐selenocysteine is slightly enriched in the cell mitochondria, with a Pearson correlation coefficient of 0.82, further indicating that AAN has better mitochondrial targeting than L‐selenocysteine. Moreover, the surface of AAN has a strong negative charge as measured by zeta potential (‐16.7 mV) (Figure 1J). Given the many reducing and nucleophilic groups (such as C‐Se and phenolic hydroxyl groups) contained in AAN, AAN has great potential to scavenge ROS with oxidative and electrophilic properties. AAN has multiple ROS scavenging activities and can effectively scavenge a variety of ROS including O_2_^·−^(Figure 1K), ·OH (Figure 1L), H_2_O_2_ (Figure 1M) and ONOO^−^ (Figure S7, Supporting Information). Finally, the stability of Se in AANs was tested by ICP·MS under oxidative stress condition (H_2_O_2_) and normal physiological condition. H_2_O_2_ can etch the C‐Se bonds in AAN and lead to the slow release of soluble selenium (the release rate can reach 90% in 72 h), while in a normal environment, AAN can remain stable and the Se release is only about 10% in 72 h (Figure 1N). The ROS‐responsive release of soluble selenium by AAN not only facilitates the uptake of selenium by renal tissue and promotes the synthesis of selenium‐containing antioxidant protein GPX4, but also effectively avoids tissue damage caused by excessive selenium ions.

### 2.2 The Distribution of AAN in DKD Mice

**Figure 2:**
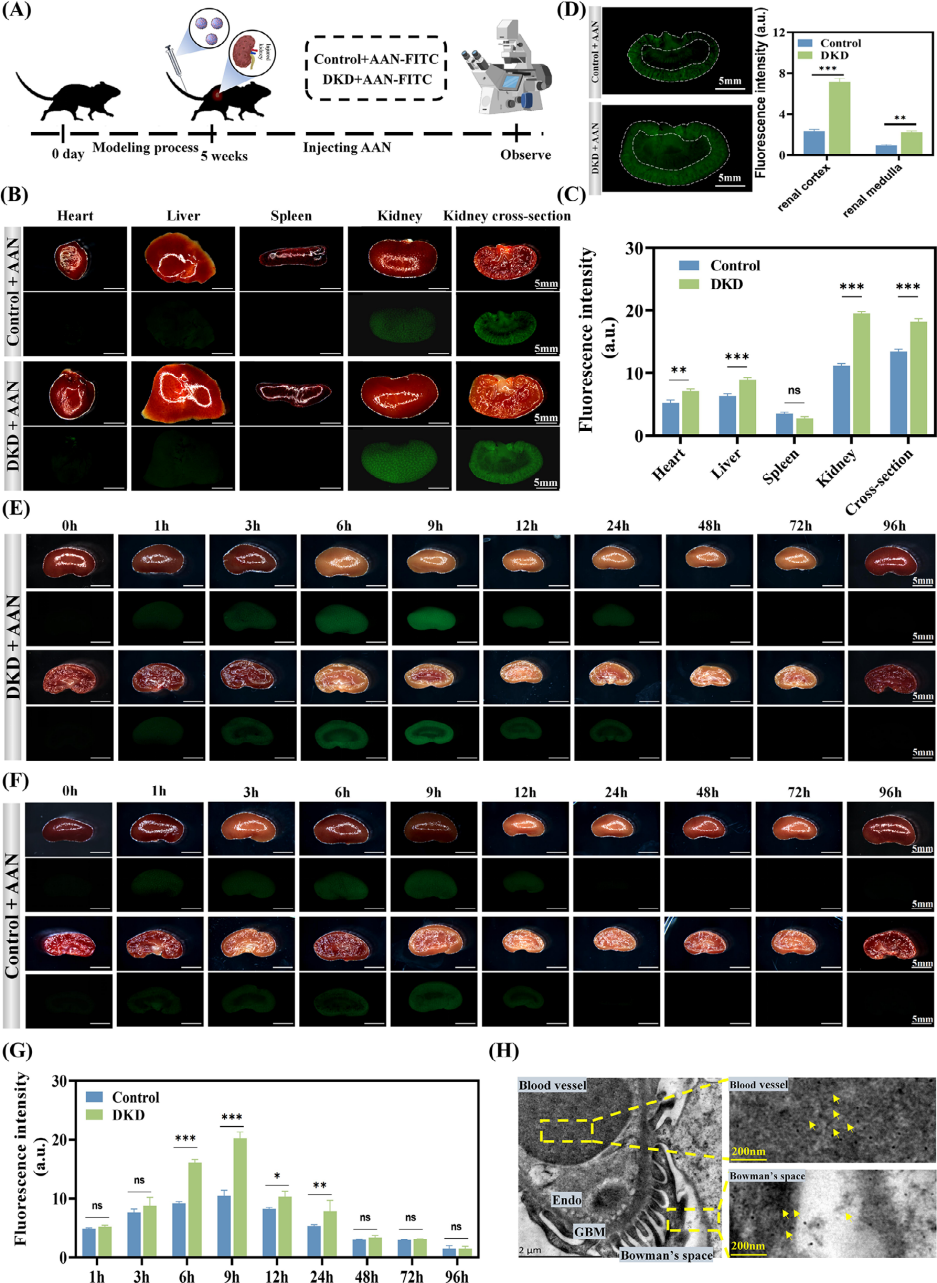
Distribution of AAN in vivo. A) C57BL/6J construction of diabetic kidney disease process and administration of AAN‐FITC process. B) Distribution of AAN‐FITC in the renal cortex and medulla of C57BL/6J mice, Scale bar: 5 mm. C‐D) Representative fluorescence images of different organs (C), and quantitative analysis (D) in DKD mice 9 h after intravenous injection of AAN, Scale bar: 5 mm. E‐G) Representative fluorescence images of kidneys in DKD mice (E), Control mice (F) and quantitative analysis (G) at various time points (0‐96 h) after intravenous injection of AAN‐FITC. H) TEM image of glomerulus after intravenous injection of AAN, Scale bar: 2 µm and 200 nm. *P < 0.05, **P < 0.01, ***P < 0.001, ns means *P* > 0.05.

The targeting of AAN to the DKD kidney was investigated. Because AAN has an ultra‐small particle size and negative charge, AAN can effectively penetrate the glomerulus and reach the renal tubules. To this end, a DKD mice model was established in C57BL/6J mice by high‐fat diet and streptozotocin (STZ) (**Figure** **2**A). After one week of adaptive feeding, C57BL/6J mice were fed a high‐fat and high‐sugar diet for 4 consecutive weeks, followed by STZ injections for 5 consecutive days. The fasting blood glucose was measured to be greater than 11.1 mmol L^−1^ for 5 consecutive days, indicating that the diabetes model was successfully established. At the 6th week, the renal function of the model mice was severely impaired and the urine albumin/creatinine ratio of the model group mice was 15 times that of the healthy group (Figure S8, Supporting Information), confirming that the DKD mice model was successfully established. Subsequently, AAN‐FITC was injected into the normal mice and the DKD model mice through the tail vein. After 9 h, the distribution of AAN in the main organs was determined by stereofluorescence microscopy. In both groups of mice, AAN mainly accumulated in the kidney tissue and had almost no distribution in other major organs such as the heart, liver, and spleen (Figure 2B‐C). Notably, AAN was mostly distributed in the cortex of the kidney, which is rich in tubular epithelial cells (more than 90% of tubules are in the cortex), while they are less distributed in the medulla (Figure 2D). The distribution of AAN in the kidneys of DKD mice was significantly higher than that in the kidneys of normal mice, about 1.95 times, which suggests that AAN can specifically target the kidneys of DKD and has high bioavailability for the treatment of DKD. The metabolism and clearance of AAN are also important for the treatment of DKD. In the DKD group, the distribution of AAN in the kidneys increased over time, reaching the highest level at 9 h. Subsequently, the distribution of AAN in the kidney gradually decreased and most of the AAN was excreted from the kidneys after 72 h (Figure 2E). AAN can also be distributed and excreted in the kidneys of healthy mice with a similar trend, except that the distribution of AAN in the healthy group was lower than that in the DKD group (Figure 2F‐G). Based on this, urine samples were collected from the mice 12 h later, and X‐ray photoelectron spectroscopy (XPS) analysis was performed on AAN in the urine. As shown in Figure S9 (Supporting Information), the primary components of the urine were C, O, N, and Se, with the Se content reaching as high as 10.47%. Fine spectral analysis of Se3d XPS revealed that the selenium peak at 55.7 eV was entirely attributed to the C‐Se bond in AAN, indicating that selenium was embedded in the carbon skeleton via covalent bonding. In DKD, renal capillary endothelial damage promotes AAN to more easily pass through the glomerulus to reach the renal tubules, which makes AAN more likely to be targeted to the kidneys in the DKD group. The kidney TEM results also confirmed that AAN was distributed in the glomerular capillaries, glomerular mesangium, renal capsule space, and tubular epithelial cells, indicating that AAN penetrated the glomerular filtration barrier and reached the renal tubules (Figure 2H). The above results show that AAN has strong kidney‐specific targeting and is mainly enriched in the cortical area rich in renal tubules. In addition, AAN is mainly excreted through the kidneys, preventing AAN from accumulating in the body and causing toxicity.

### 2.3 AAN Alleviates Renal Injury in DKD Mice

The therapeutic effect of AAN on DKD was investigated. The high lesion targeting of AAN provides a guarantee for AAN to exert its adaptive antioxidant effect. We first confirmed the treatment doses and treatment cycles in the preliminary experiments (Figures S10–S14, Supporting Information). The two dose groups of 5 mg kg^−1^ and 10 mg kg^−1^ showed the most significant therapeutic effect in terms of weight changes and kidney functions (Figures S10–S13, Supporting Information). Therefore, 5 mg kg^−1^ and 10 mg kg^−1^ were used for treatment. Since we observed a significant effect at 6 weeks, and there was no significant difference in 24‐hour urine protein, serum creatinine, blood urea nitrogen, and urine albumin/creatinine compared to 8 weeks and 12 weeks (Figure S14, Supporting Information), adhering to the principle of precise treatment, we chose a 6‐week treatment to evaluate the efficacy of AAN. We also used a common antioxidant, NAC, at the same dose (10 mg kg^−1^) as a Control. And NAC treatment of 6 weeks showed no effect on DKD (Figure S15, Supporting Information).

**Figure 3:**
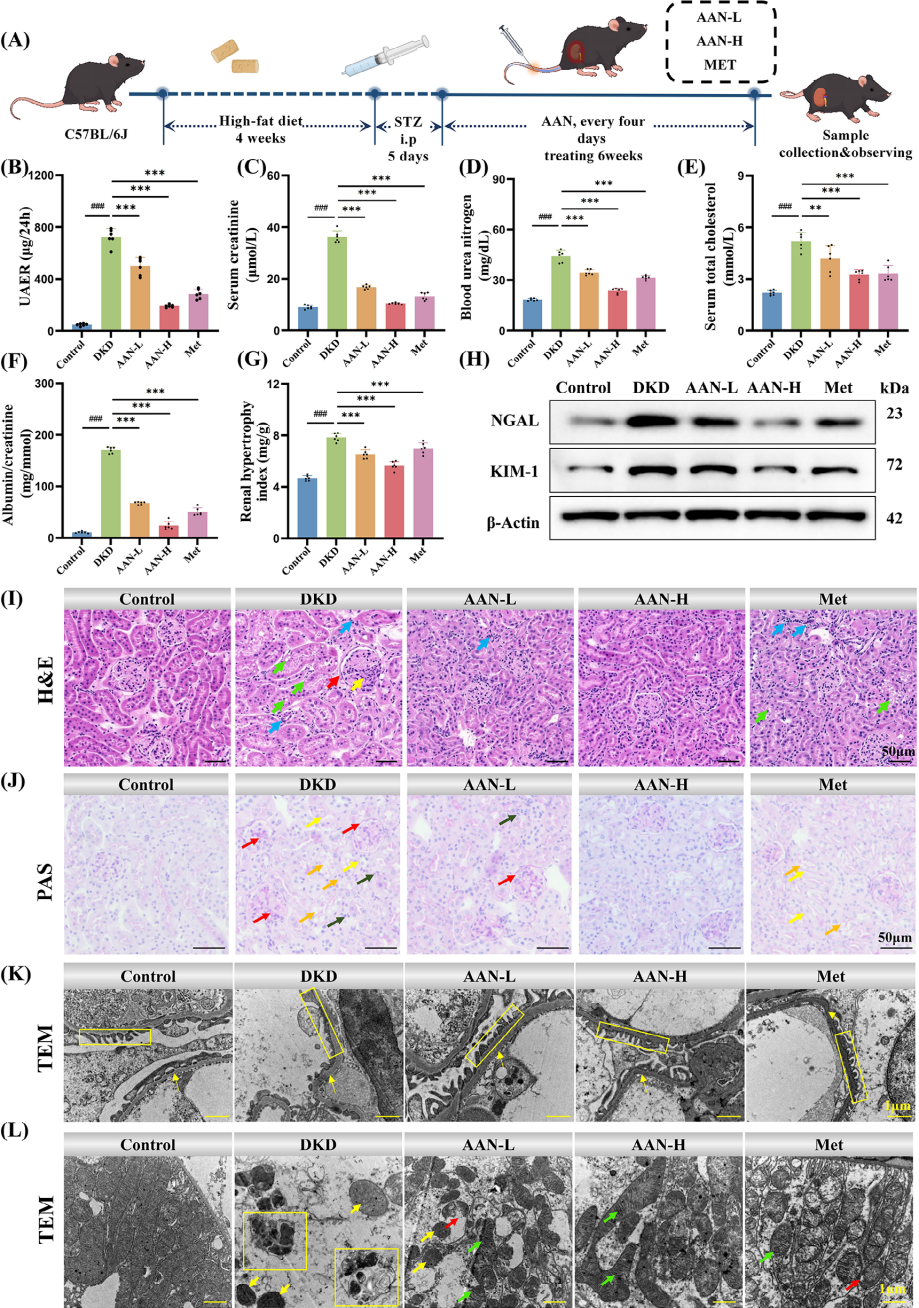
The therapeutic effect of AAN in DKD mice. A) Schematic illustration of the establishment and treatment schedule of DKD mice. B‐G) 24h urinary albumin excretion rates (B), blood creatinine (C), blood urea nitrogen (D), blood cholesterol (E), urinary creatinine/albumin ratio (F), and renal index (G) in different groups of mice. H) Western Blotting of KIM‐1 and NGAL in kidney tissues of different groups of mice. (I‐L) HE‐staining I), PAS glycogen‐staining J), TEM images of renal glomerular K), TEM images of renal tubular (L) in different groups of mice. Data represent means ± S.D. from three or six independent replicates. ^###^ *P* < 0.01 vs Control group; **P < 0.01, ***P < 0.001 vs DKD group.

Considering the long‐term excretion of AAN by kidney in DKD mice, two different doses (5 mg kg^−1^ and 10 mg kg^−1^) of AAN were administered by intravenous injection every 4 days for 6 weeks. Metformin was used as a positive Control (**Figure** **3**A). Metformin is clinically used to treat diabetic nephropathy, moreover, Metformin can inhibit electron transport chain (ETC) complex I and reduce mitochondria ROS.^[^ ^21^, ^22^ ^]^ Therefore, Metformin was used as a positive drug in this study. After treatment, renal function in different groups was evaluated by 24‐hour proteinuria, serum creatinine (CRE), blood urea nitrogen (BUN), serum total cholesterol, and urine albumin/creatinine. Compared with the Control group, the renal function of mice in the DKD group was significantly impaired, and the 24‐hour urine protein (Figure 3B), CRE (Figure 3C), BUN (Figure 3D), total cholesterol (Figure 3E) and urine albumin/creatinine ratio (Figure 3F) were significantly increased, which were 14.9 times, 1.67 times, 4 times, 2.42 times and 2.34 times that of the Control group, respectively. As a strong contrast, both groups of AAN at different doses could significantly reduce these indicators, and the high‐dose group could restore renal function to normal levels, and the effect was even better than that of the Met group. Renal hypertrophy is one of the early pathological features of diabetic nephropathy, manifested by the proliferation and enlargement of renal cells (such as glomerular mesangial cells and renal tubular epithelial cells) and renal tissue fibrosis.^[^ ^23^, ^24^ ^]^ As shown in Figure 3G, AAN can significantly alleviate the increase of renal hypertrophy index in DKD, and the effect of AAN high‐dose group is better than that of Met group.

The effect of AAN on alleviating kidney damage was further investigated in DKD. Western Blotting (WB) verified the expression of kidney injury molecule‐1 (KIM‐1) and neutrophil gelatinase‐associated lipocalin (NGAL) (protein biomarker closely related to renal injury) in different groups. The levels of KIM‐1 and NGAL in the kidneys of mice in the DKD group were 2.10 and 2.03 times higher than those in the Control group, respectively. AAN treatment effectively reduced the expression of KIM‐1 and NGAL in the kidneys of DKD mice. In particular, the high‐dose AAN group was extremely close to the value of the Control group, indicating that it has the effect of alleviating renal tubular damage (Figure 3H; Figure S16, Supporting Information). Through Hematoxylin and eosin (H&E) staining, compared with the Control group, the renal tubule tissue of the DKD group mice was severely damaged, with glomerular hypertrophy (yellow arrow), enlarged Bowman's membrane space (red arrow), tubular cavitation (green arrow), and inflammatory cell infiltration (blue arrow). After AAN treatment, the morphology and structure of renal tissue were effectively restored (Figure 3I). Through periodic acid schiff (PAS) glycogen staining, the renal tubular basement membrane was thickened (yellow arrows) and the epithelium was vacuolated (orange arrows) in the renal tissue of mice in the DKD group. Moreover, in the DKD group, glomeruli were atrophied, the basement membranes of glomeruli and renal capsules were homogeneously thickened (red arrows), and a large number of inflammatory cells infiltrated the renal interstitium (green arrows). After treatment with both doses of AAN and Metformin, significant improvements were observed in both renal tubules and glomeruli, with the most significant improvement in the high‐dose AAN group (Figure 3J). By TEM, compared with the Control group, the basement membrane (yellow arrow) in the DKD group was homogeneously thickened, the podocytes (orange wireframe) were edematous, detached, and the foot processes were fused. After AAN treatment, the glomerular morphology was significantly restored, the basement membrane thickness was reduced, and although the podocytes were still slightly edematous, the regular arrangement of the foot processes was clearly visible (Figure 3K). Finally, the proximal tubule area between the different groups was directly observed by TEM (Figure 3L). The mitochondria in the Control group were numerous, dense, and neatly arranged, with no abnormal mitochondrial state. Mitochondrial morphological changes are typical features of ferroptosis.^[^ ^25^ ^]^ The mitochondria in the DKD group mice showed typical characteristics of ferroptosis, including mitochondrial atrophy (yellow frame), mitochondrial membrane fragmentation (red arrow), and reduced (yellow arrow) or even disappeared mitochondrial cristae, indicating that ferroptosis occurred in the kidneys of DKD mice. In comparison, in the high‐dose AAN group, the number of mitochondrial cristae increased (green arrows), the volume of mitochondria enlarged, no mitochondrial membrane fragmentation was observed, and the mitochondrial morphological changes were significantly improved, indicating that AAN treatment effectively alleviated mitochondrial damage associated with ferroptosis. The above results show that AAN can effectively restore renal function and alleviate kidney injury in DKD, and the effect of AAN in the high‐dose group is much better than that of Metformin.

### 2.4 AAN Inhibits Tubular Ferroptosis in DKD

**Figure 4:**
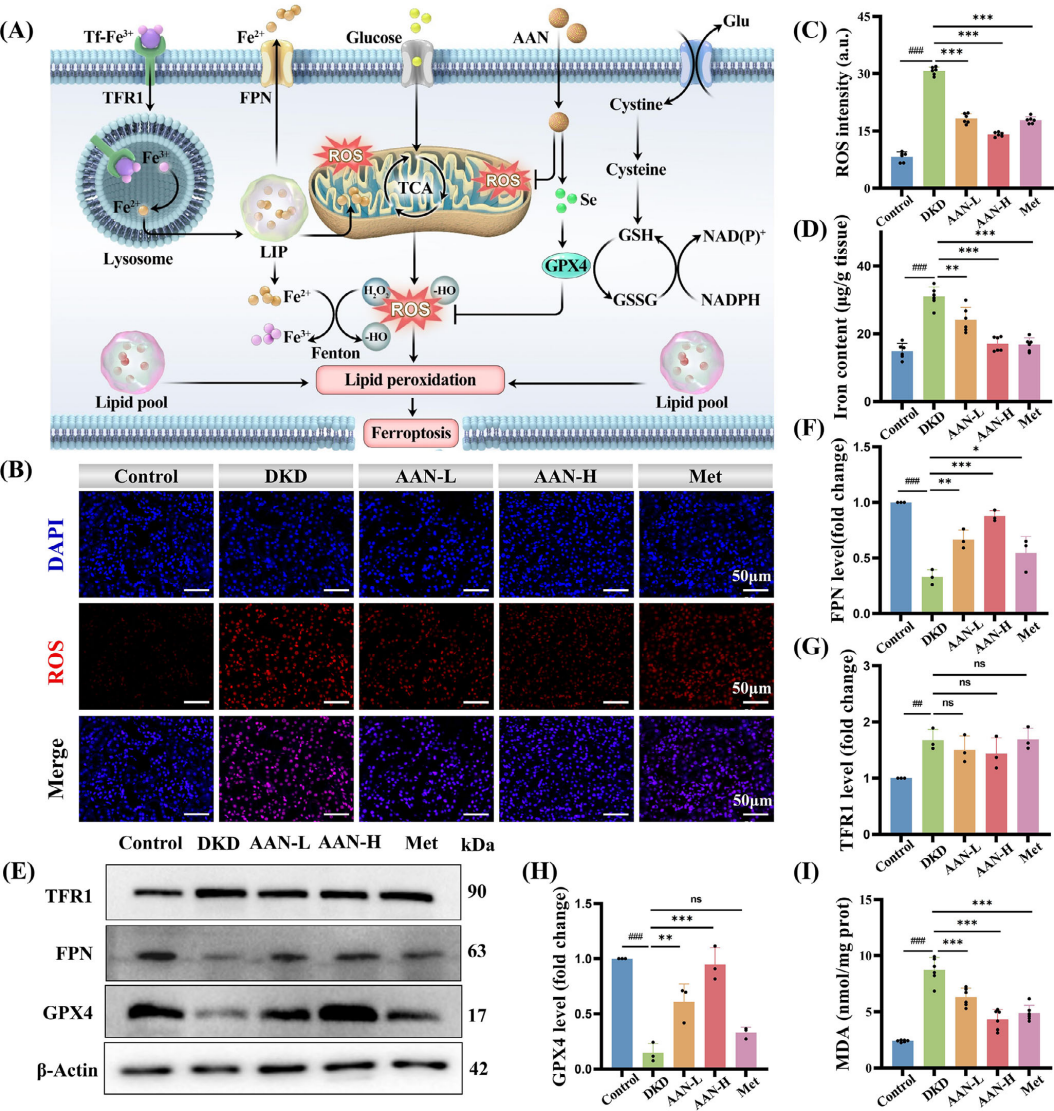
AAN inhibits tubular ferroptosis in DKD. A) Schematic diagram of ferroptosis mechanism. B‐C) representative images of DHE (B) and quantitative analysis (C), Scale bar: 50 µm. D) the iron content in the kidney tissue of different mice. E‐H) Western blot analysis of ferroptosis‐related protein expression levels in the kidney tissue of different mice (E) and quantitative analysis of FPN (F), TFR1 (G), GPX4 (H). I) MDA content in the kidney tissue of different mice. Data represent means ± S.D. from three independent replicates. ^##^ *P* < 0.01, ^###^ *P* < 0.001 vs Control group; *P < 0.05, **P < 0.01, ***P < 0.001 vs DKD group. ns means *P* > 0.05.

Ferroptosis is a type of programmed cell death caused mainly by iron‐dependent oxidative damage.^[^ ^26^ ^]^ Excessive iron deposition in the renal tubular tissue can lead to a significant increase in ROS levels, resulting in lipid peroxidation, mitochondrial membrane damage, and ultimately tubular cell ferroptosis.^[^ ^27^, ^28^ ^]^ Therefore, AAN can not only inhibit ferroptosis by directly eliminating ROS, but also ensure that the soluble Se released is subsequently converted into GPX4. As a core antioxidant enzyme, GPX4 can adaptively eliminate ROS and lipid peroxidation, playing a role in long‐term inhibition of ferroptosis^[^ ^29^ ^]^ (**Figure** **4**A). To this end, the ROS levels in the renal tubular tissues of different groups were analyzed by dihydroethidium (DHE) staining. Compared with the Control group, the ROS level in the DKD group was significantly increased. AAN can effectively reduce the ROS level, especially high‐dose AAN (Figure 4B‐C). By Tissue Iron Content Assay, the iron content in the kidney tissue of mice in the DKD group increased significantly, while AAN treatment significantly decreased the iron content (Figure 4D). Intracellular iron is mainly regulated by transferrin receptor 1 (TFR1) and ferroportin (FPN). Specifically, Fe is taken up through TFR1, while excess Fe is transferred out of the cell through FPN. Increased expression of TFR1 and decreased expression of FPN both lead to accumulation of Fe, which in turn promotes ferroptosis.^[^ ^30^, ^31^, ^32^ ^]^ Similarly, GPX4, as a core antioxidant defense protein, participates in the ferroptosis process and can inhibit ferroptosis. However, GPX4 content decreases when ferroptosis occurs.^[^ ^29^ ^]^ Through WB, the expression of FPN and GPX4 in the DKD group was significantly reduced, and the expression of TFR was increased, indicating the occurrence of ferroptosis in DKD (Figure 4E). After AAN treatment, especially high‐dose AAN, the levels of FPN were effectively increased (Figure 4F). The expression of TFR1 decreased under AAN treatment, but it was not statistically significant (Figure 4G). Therefore, AAN reduces iron deposition mainly by increasing the expression of FPN to exclude iron from renal tubular cells. Notedly, the expression of GPX4 was significantly increased at both doses of AAN (Figure 4H). Under high‐dose AAN treatment, the expression of GPX4 has returned to a healthy level, which is much better than Metformin. This fully demonstrates that Se released by AAN under ROS conditions has been successfully converted into GPX4. Malondialdehyde (MDA) is one of the final products of lipid peroxidation and an important indicator for evaluating ferroptosis. As shown in Figure 4I, the content of MDA was significantly increased in the kidney tissue of the DKD group, and AAN could significantly reduce the content of MDA. In addition, the contents of GSH and GSSG/GSH in renal tissues of different groups were analyzed. The contents of GSH and GSSG/GSH in the kidneys of mice in the DKD group were significantly decreased. After AAN treatment, the GSH content (Figure S17A, Supporting Information) and GSSG/GSH value (Figure S17B, Supporting Information) were restored, and the high dose of AAN was close to the normal level. The above data fully demonstrate that AAN can not only eliminate ROS, but also the Se released by it can be effectively converted into GPX4 to enhance the endogenous antioxidant capacity of DKD mice and effectively slow down ferroptosis.

### 2.5 AAN Inhibits High Glucose‐Induced Ferroptosis in HK‐2 Cells

**Figure 5:**
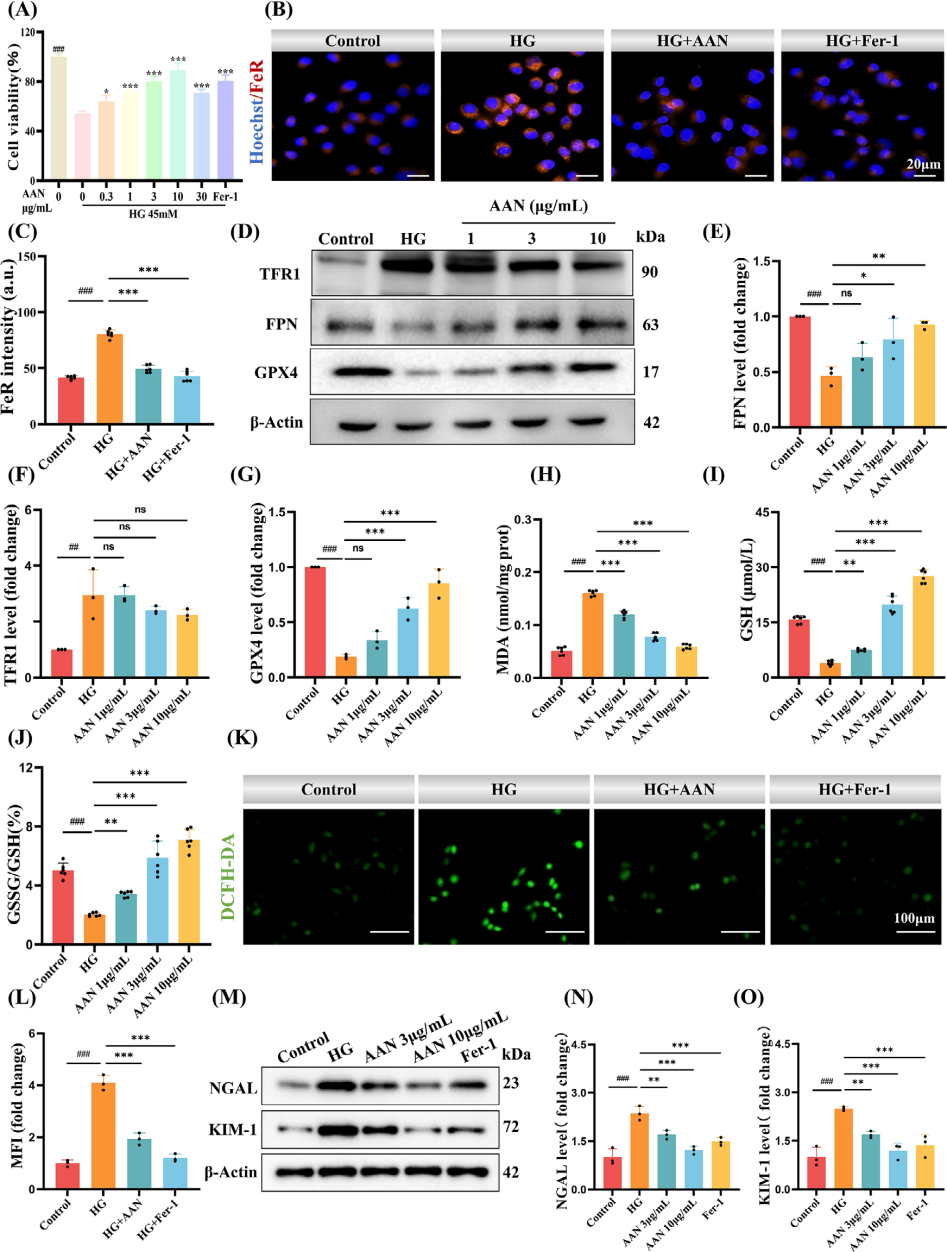
AAN inhibits high glucose‐induced ferroptosis in HK‐2 cells. A) Cell viability of high glucose‐induced HK‐2 cells treated with different concentrations of AAN and positive Control Fer‐1. B‐C) Representative images of FeRhoNox‐1(FeR)‐staining in high glucose‐induced HK‐2 cells treated with AAN and positive Control Fer‐1 (B), and quantitative analysis (C), Scale bar: 20 µm, EX/EM = 545/585nm. D‐G) Western blot analysis of ferroptosis‐related protein expression levels in HK‐2 cells(D) and quantitative analysis of FPN (E), TFR1 (F) and GPX4 (G). H‐J) MDA contents (H), GSH (I), GSSG/GSH ratio (J) in HK‐2 cells. K‐L) Representative images of DCFH‐DA‐staining in high glucose‐induced HK‐2 cell treated with AAN and positive Control Fer‐1 (K), and quantitative analysis (L), Scale bar: 100 µm, EX/EM = 488/525 nm. M‐O) Western blot analysis of kidney injury‐related protein expression levels in high glucose‐induced HK‐2 cells treated with different concentrations of AAN and positive Control Fer‐1 (M) and quantitative analysis of NGAL (N) and KIM‐1(O). Data represent means ± S.D. from three independent replicates. *^##^P* < 0.01, *^###^P* < 0.001 vs Control group; *P < 0.05, **P < 0.01, ***P < 0.001 vs HG group. ns means *P* > 0.05.

In order to verify the alleviating effect of AAN on ferroptosis at the cellular level, a renal injury model was constructed using high glucose (HG) ‐induced HK‐2 cells. To verify the inhibitory effect of AAN on ferroptosis, we treated HK‐2 cells with AAN and adopted the ferroptosis inhibitor Fer‐1 as a positive Control. CCK8 assay was used to explore the therapeutic concentration of AAN for HK‐2 induced by high glucose. To explore whether the high‐glucose‐induced decrease in cell viability is related to ferroptosis, we added the ferroptosis inhibitor Fer‐1 to HK‐2 cells. Fer‐1 treatment significantly improved cell viability under high glucose conditions, indicating that high glucose does induce HK‐2 ferroptosis. Notedly, AAN significantly improved the viability of HK‐2 cells under high glucose conditions, and the effects of 3 µg mL^−1^ and 10 µg mL^−1^ were the most significant (**Figure** **5**A). It is worth noting that we compared the same concentration of selenium undoped carbon nanodots (C NDs) (similar in size) with AAN and found that AAN exhibited excellent therapeutic effects, while selenium undoped carbon nanodots (similar in size) had no significant effect (Figure S18, Supporting Information). This further suggests that it is the slowly released Se in AAN that plays a role, rather than the skeletal structure of AAN. In addition, to demonstrate the superior control release ability of AAN, L‐selenocysteine was used as a Control. As shown in Figure S19 (Supporting Information), under the condition of 3 µg mL^−1^, both L‐selenocysteine and AAN are non‐toxic. When the concentration reaches 10 µg mL^−1^ and 20 µg mL^−1^, L‐selenocysteine releases excessive se, causing toxicity, while AAN does not produce significant toxicity due to its excellent release control ability. To further confirm effects of AAN on ferroptosis, HK‐2 cells were induced with Erastin (a ferroptosis inducer) to induce direct ferroptosis. Similarly, AAN could restore the decrease in cell viability caused by Erastin(Figure S20, Supporting Information). Intracellular Fe^2+^ overload is considered to be the initial event of ferroptosis, and the accumulation of Fe^2+^ is closely related to iron transport‐related receptors.^[^ ^32^ ^]^ Through the Fe^2+^‐specific probe‐FeRhoNox‐1 (FeR), compared with the Control group, the level of Fe^2+^ in HK‐2 cells induced by high glucose was significantly increased, and the intracellular Fe^2+^ concentration was significantly decreased after treatment with AAN and Fer‐1(Figure 5B‐C). Moreover, the Fe^2+^ level in HK‐2 cells incubated with Erastin was higher than that in the Control group, and the Fe^2+^ level was significantly decreased after incubation with AAN and Fer‐1(Figure S21, Supporting Information). The effect of AAN on the expression levels of FPN, TFR, and GPX4 in high glucose‐induced HK‐2 cells was detected by WB (Figure 5D). As shown in Figure 5E‐F, AAN can significantly increase the expression of FPN and slightly reduce the expression of TFR, indicating that AAN can promote the excretion of Fe^2+^ into the extracellular space and reduce the level of intracellular Fe^2+^. Excitingly, AAN can also greatly promote the expression of GPX4(Figure 5G), indicating that Se released by AAN under high ROS levels can be converted into GPX4. MDA levels (Figure 5H), GSH level (Figure 5I), and GSSG/GSH values (Figure 5J) were also measured, and the results were consistent with those obtained from the in vivo experiment in Section 2.4, demonstrating that AAN can inhibit ferroptosis, enhance intracellular antioxidant capacity, and reduce lipid peroxidation.

The occurrence of ferroptosis is inevitably accompanied by an increase in intracellular ROS content, resulting in oxidative damage.^[^ ^27^ ^]^ Compared with the Control group, the ROS level in HK‐2 cells in the high glucose group was significantly increased by DCFH‐DA probe, while the ROS level in the AAN group and Fer‐1 group was significantly decreased (Figure 5K‐L). Similarly, AAN can also significantly reduce the level of ROS in HK‐2 cells induced by Erastin (Figure S22, Supporting Information). Further, through WB, the levels of NGAL and KIM‐1 in HK‐2 cells in the high‐glucose group were significantly higher than those in the Control group, proving that high glucose can cause HK‐2 cell damage (Figure 5M). After treatment with different concentrations of AAN and Fer‐1, the levels of NGAL (Figure 5N) and KIM‐1 (Figure 5O) decreased significantly, and the levels of NGAL and KIM‐1 in the high‐dose AAN group returned to levels close to those in the Control group. AAN also reduced the levels of NGAL and KIM‐1 in HK‐2 cells induced by Erastin (Figure S23, Supporting Information). The above experimental results confirmed that high glucose‐induced HK‐2 cells underwent ferroptosis, and AAN could inhibit ferroptosis, increase intracellular antioxidant levels, and alleviate cellular oxidative damage.

### 2.6 Mitochondrial Protection of AAN

**Figure 6:**
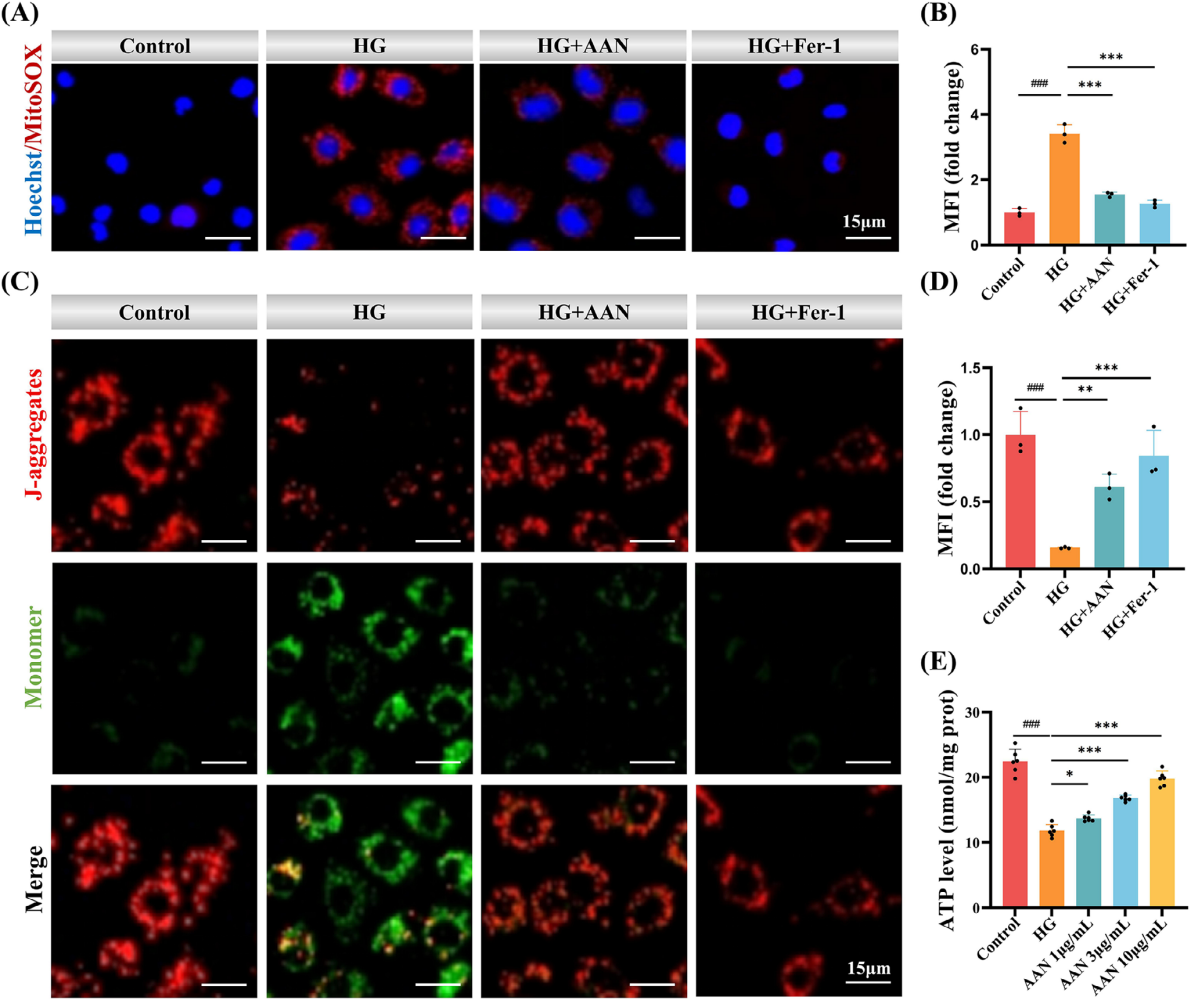
Mitochondrial protection of AAN. A‐B) Representative images of MitoSox‐stained in high glucose‐induced HK‐2 cell treat with AAN and positive Control Fer‐1 (A), and quantitative analysis (B), Scale bar: 15 µm, EX/EM = 396/610 nm. C‐D) Representative images of JC‐1‐stained in high glucose‐induced HK‐2 cell treated with AAN and positive Control Fer‐1 (C), and quantitative analysis (D), Scale bar: 15 µm, EX/EM = 490/530 nm. E) ATP level in high glucose‐induced HK‐2 cell treat with different concentrations of AAN. Data represent means ± S.D. from three independent replicates. *^###^P* < 0.001 vs Control group; *P < 0.05, **P < 0.01, ***P < 0.001 vs HG group.

Ferroptosis is closely related to mitochondrial damage, and mitochondrial dysfunction is the main causes of renal tubular injury in DKD.^[^ ^8^ ^]^ Mitochondria are the main site of intracellular ROS generation.^[^ ^33^ ^]^ In DKD, many toxic metabolites (such as fatty acids) interfere with the mitochondrial electron transport chain and induce the generation of a large amount of mtROS.^[^ ^14^, ^34^ ^]^ Excessive Fe^2+^ further promotes the generation of mtROS in mitochondria through the Fenton reaction. Through MitoSOX, the mtROS level in HK‐2 cells stimulated by high glucose increased significantly, about 3.4 times that of the Control group cells. AAN and Fer‐1 reduced the production of mtROS induced by high glucose by more than 60% (**Figure** **6**A‐B). Ferroptosis‐induced ROS can damage the mitochondrial membrane, leading to decreased mitochondrial membrane potential (MMP) and mitochondrial dysfunction.^[^ ^35^ ^]^ To this end, 5,5',6,6'‐tetrachloro‐1,1',3,3'‐tetramethylbenzimido‐dazolylcarbocyanine iodide (JC‐1) dye was used as a sensitive MMP probe. As shown in Figure 6C‐D, the MMP of HK‐2 cells stimulated by high glucose was significantly decreased, while AAN administration significantly increased MMP. MMP is a prerequisite for mitochondria to carry out oxidative phosphorylation and produce ATP.^[^ ^36^ ^]^ Mitochondria are the main site of ATP synthesis, and ATP levels can be used as a direct indicator to assess the functional status of mitochondria. Figure 6E shows that the ATP of high‐glucose‐induced HK‐2 cells was significantly reduced, about 53% of the Control group. After treatment with AAN, the ATP level gradually recovered. Subsequently, AAN was used to treat Erastin‐induced HK‐2 cells to investigate the effect of AAN on ferroptosis. As shown in Figure S24 (Supporting Information), AAN can significantly reduce the mtROS level in HK‐2 cells induced by Erastin. AAN can also effectively restore the MMP (Figure S25, Supporting Information) and ATP production rate (Figure S26, Supporting Information) of HK‐2 cells induced by Erastin. The above results further prove that AAN can effectively reduce mitochondrial damage induced by ferroptosis and restore mitochondrial function.

### 2.7 AAN Inhibits Inflammation and Fibrosis in DKD

**Figure 7:**
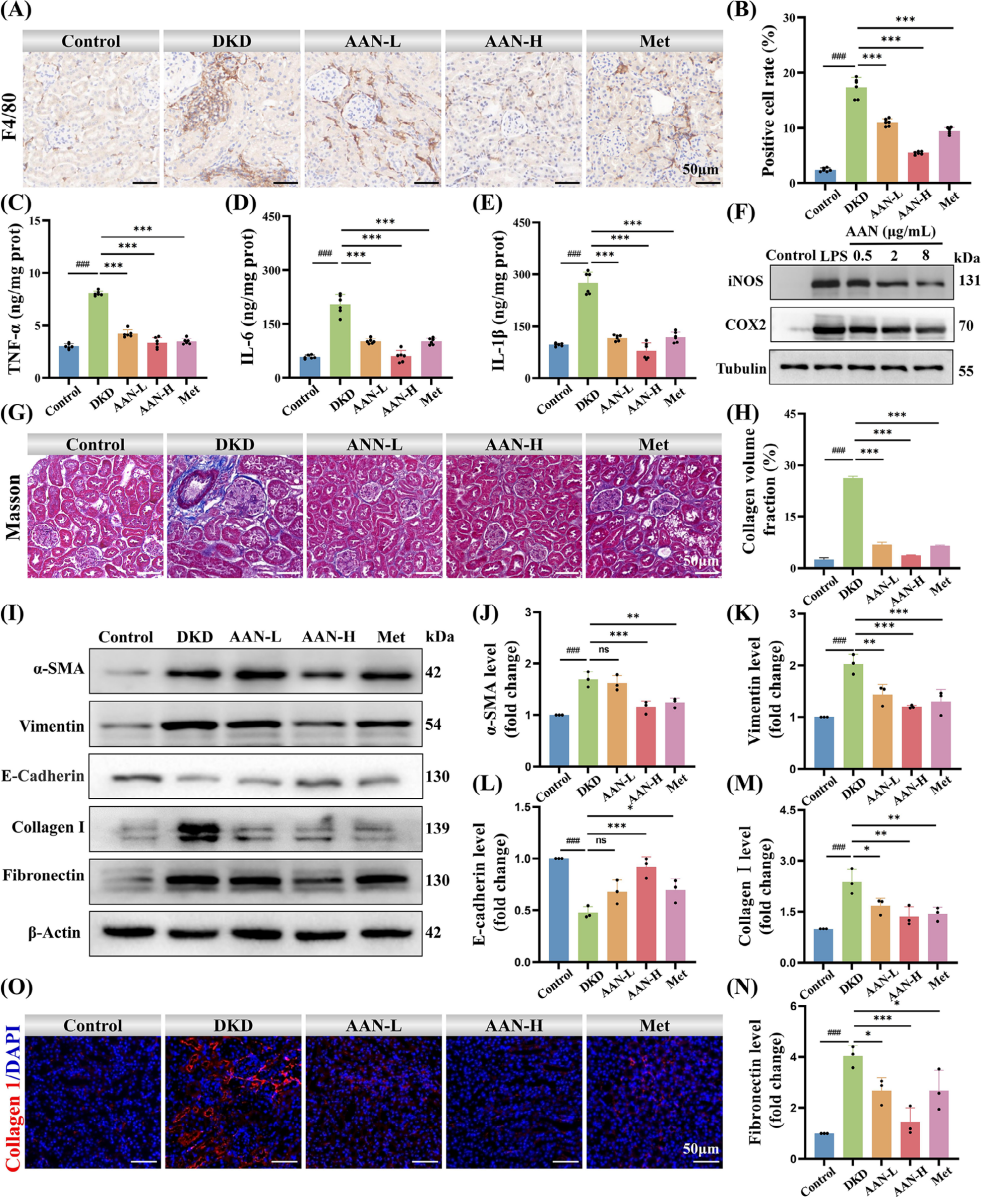
AAN inhibits inflammation and fibrosis in DKD. A‐B) F4/80 immunohistochemical staining images (A) and F4/80 positive cells rate (B) of mice in different groups, Scale bar: 50 µm. C‐E) Enzyme‐linked immunosorbent assays of TNF‐α (C), IL‐6 (D) and IL‐1β (E) in renal tissue homogenates of mice in different groups. F) Western blot analysis of INOS and COX‐2 expression levels in LPS‐induced HK‐2 cells treated with different concentrations of AAN. G‐H) Representative images of Masson‐stained in kidney of different mice (G), and Collagen fiber area statistics (H), Scale bar: 50 µm. I‐N) Western blot analysis of fibrosis‐related protein expression levels in kidney (I), and quantitative analysis of α‐SMA (J), Vimentin (K), E‐cadherin (L), Collagen I (M) and Fibronectin (N). O) Representative images of Collagen I‐stained in kidney of different mice, Scale bar: 50 µm. Data represent means ± S.D. from three independent replicates. *^###^P* < 0.001 vs Control group; *P < 0.05, **P < 0.01, ***P < 0.001 vs HG group. ns means *P* > 0.05.

Compared with other forms of death, ferroptosis is more likely to release damage‐associated molecular patterns (DAMPs) and induce inflammation.^[^ ^37^ ^]^ The infiltration of macrophages in the renal tissue of mice in different groups was analyzed by immunohistochemical staining of macrophage marker F4/80. The positive points in the DKD group were 7.21 times that of the normal group, and the low‐dose AAN group, high‐dose AAN group, and Met group were 4.5 times, 2.28 times, and 3.95 times that of the normal group (**Figure** **7**A‐B), respectively, indicating that the AAN and Met groups can effectively eliminate macrophage infiltration. In particular, the effect of the high‐dose AAN group was even better than that of Metformin. Subsequently, the inflammatory factors TNF‐α, IL‐6, and IL‐1β in the kidney tissues of each group were determined by enzyme‐linked immunosorbent assay. Compared with the Control group, the levels of inflammatory factors, including TNF‐α (Figure 7C), IL‐6 (Figure 7D) and IL‐1β (Figure 7E) in the renal tissue in the DKD group increased significantly. After treatment with low‐dose and high‐dose AAN and Metformin, the levels of the above inflammatory factors were significantly reduced. Especially, the levels of the above inflammatory factors reduced to near normal levels in the high‐dose AAN treatment group, indicating that AAN can effectively reduce the inflammatory response during DKD. DAMPs bind to Toll‐like receptors on macrophages and induce macrophage polarization to the pro‐inflammatory (M1) type, which in turn secretes various inflammatory factors.^[^ ^38^ ^]^ In vitro, Macrophage RAW264.7 cells were activated by lipopolysaccharide (LPS) stimulation. Through WB, macrophage RAW264.7 cells transformed into activated M1 macrophages after being LPS, and the expression of iNOS increased significantly. After treated with different concentrations of AAN, the expression of iNOS was gradually downregulated (Figure 7F; Figure S27, Supporting Information). AAN also significantly reduced the expression of cyclooxygenase‐2 (COX‐2), an inducible enzyme closely related to inflammatory response (Figure 7F; Figure S28, Supporting Information). The levels of inflammatory factors (TNF‐α, IL‐6, and IL‐1β) in RAW264.7 cells induced by LPS were significantly increased. After AAN treatment, the levels of inflammatory factors were significantly decreased (Figure S28, Supporting Information).

Next, the effect of AAN in alleviating renal fibrosis was verified in DKD. Masson staining was used to detect the degree of renal fibrosis in each group. The volume of collagen fibers in the different doses of AAN groups was significantly lower than that in the DKD group, especially in the high dose AAN group, which was 85% lower than that in the DKD group, indicating that AAN can effectively improve tubular interstitial fibrosis in DKD mice (Figure 7G‐H). Epithelial‐mesenchymal transition (EMT) of renal tubular epithelial cells is a core event in renal fibrosis.^[^ ^39^ ^]^ During EMT, the expression of E‐cadherin decreases, while the expression of vimentin and α‐SMA increases, accompanied by the secretion and deposition of Collagen I and Fibronectin.^[^ ^40^, ^41^ ^]^ Through WB, the levels of α‐SMA and vimentin in the renal tissue homogenate of DKD group mice were significantly increased compared with those of Control group, while the expression of E‐cadherin was reduced, and the deposition of Collagen I and Fibronectin increased, indicating that EMT process occurred in the renal tissue of DKD group mice (Figure 7I). After AAN treatment, the expressions of α‐SMA (Figure 7J) and vimentin (Figure 7K) decreased, the expression of E‐cadherin decreased (Figure 7L), and the secretions of Collagen I (Figure 7M) and Fibronectin (Figure 7N) decreased compared with the DKD group, indicating that AAN can effectively alleviate renal fibrosis in DKD. Collagen I immunofluorescence further confirmed that AAN can effectively reduce renal fibrosis in DKD (Figure 7O). In vitro, the expression levels of E‐cadherin, vimentin, α‐SMA, Collagen I and Fibronectin were detected by WB in HK‐2 cells induced by high glucose. HK‐2 cells showed significant fibrotic characteristics under high glucose stimulation (Figure S29, Supporting Information). The fibrosis of HK‐2 cells induced by high glucose was improved in a concentration‐dependent manner under AAN treatment. TGF‐β is a key fibrotic factor.^[^ ^42^ ^]^ Finally, the expression level of TGF‐β protein in kidney tissues in different groups was detected by WB. The TGF‐β in the kidneys of the DKD group was 1.98 times that of the normal group, while AAN treatment effectively reduced the TGF‐β expression in the kidneys of DKD mice extremely close to the value of the Control group (Figure S30, Supporting Information). In summary, the above results indicate that AAN can effectively inhibit inflammation and alleviate renal fibrosis in DKD.

### 2.8 The Excellent Biocompatibility of AAN

**Figure 8:**
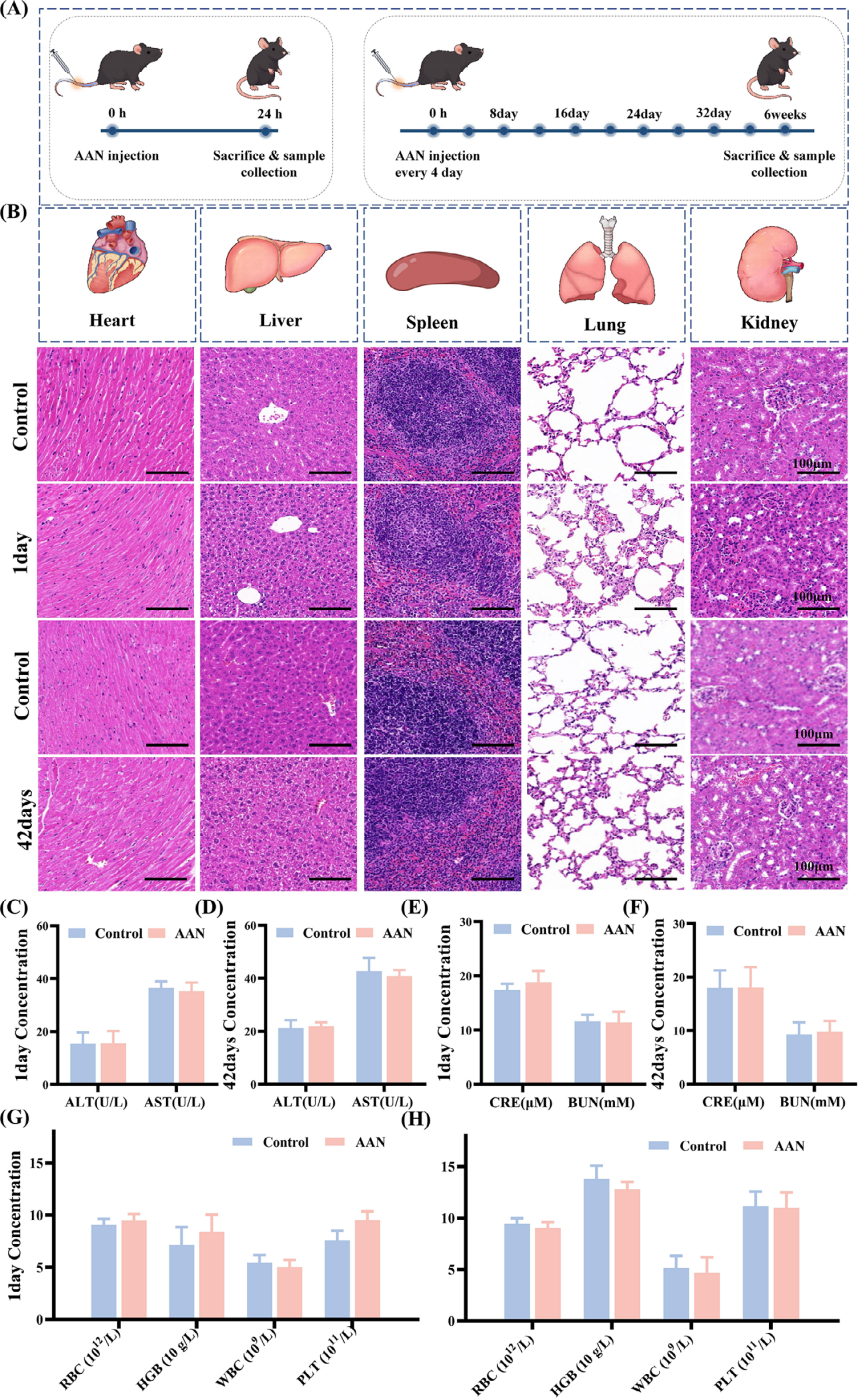
The excellent biocompatibility of AAN. A) Schematic illustration of short‐term and long‐term treatment protocol for biosafety evaluation of AAN. B) Representative H&E‐stained images of major organs from C57BL/6J mice after short‐term (1 day) and long‐term (28 days) administration of AAN or 1× PBS. C‐D) Liver function index of short‐term (C) treatment and long‐term treatment (D) in different groups of mice. E‐F) Renal function index of short‐term (E) treatment and long‐term treatment (F) in different groups of mice. G‐H) blood routine examination of short‐term (G) treatment and long‐term treatment (H) in different groups of mice.

Finally, the biocompatibility of AAN was evaluated to lay the foundation for subsequent clinical translation. The CCK8 results showed that AAN did not cause toxicity in HK‐2 cells and RAW264.7 cells when incubated at a concentration of 20 µg mL^−1^ or below (Figure S31, Supporting Information). The therapeutic concentration of AAN is 10 µg mL^−1^, which is far lower than the safe concentration of 20 µg mL^−1^, indicating that AAN exhibits excellent biosafety at the cellular level. in vivo, acute toxicity of AAN was assessed on day 1 by intravenous injection of high‐dose AAN (20 mg kg^−1^) and therapeutic dose (10 mg kg^−1^) into C57BL/6J mice. Long‐term toxicity was assessed by intravenous injection of high‐dose AAN (20 mg kg^−1^) and therapeutic dose (10 mg kg^−1^) every four days for 6 weeks (**Figure** **8**A). Through HE staining of important organs, AAN at both doses did not cause pathological damage to the heart, liver, spleen, lungs and kidneys of C57BL/6J mice in both the short and long term, indicating that AAN does not cause damage to major organs in the short or long term (Figure 8B). Through the detection of hematological indicators and liver and kidney function indicators, AAN at these two doses had no effect on the hematological indicators (RBC, HGB, PLT, WBC), liver function (ALT, AST) and kidney function (CRE, BUN) of C57BL/6J mice in both the short and long term, indicating that AAN did not affect hematopoiesis, immunity, liver function and kidney function. After 12 weeks of administration, we examined the pathology of important organs in the Control and AAN 10 mg kg^−1^ groups and found no obvious abnormal changes (Figure S32, Supporting Information). The above results all indicate that AAN has good biosafety both in vivo and in vitro, demonstrating its good potential for clinical transformation. In addition, we continuously monitored the weight changes of the mice for the first 14 days and found no significant weight changes and did not observe any behavioral abnormalities in the mice, further indicating that the AAN has good biocompatibility (Figure S33, Supporting Information).

## 3 Conclusion

Although it has been clearly established that renal tubular mtROS and the ensuing mitochondrial dysfunction are the initiation and core pathology of DKD, the low focal targeting and poor adaptability of traditional antioxidant therapies limit their application. Here, we combined precise renal tubular mitochondria targeting with an adaptive antioxidant strategy to develop an ultra‐small AAN with negative charge and aminoacetic acid group. AAN can pass through the glomerular barrier to reach the renal tubular cells and then achieve precise localization in mitochondria due to the high affinity of the aminoacetic acid group for the mitochondrial outer membrane protein TOM20. AAN can efficiently eliminate mtROS on mitochondria. Moreover, AAN slowly releases soluble Se under ROS stimulation and is converted into GPX4, thereby continuously and adaptively exerting an antioxidant effect. The combination of mitochondrial targeted elimination of mtROS and subsequent adaptive antioxidant action ultimately achieved a dosing interval of up to 96 hours, overcoming the shortcomings of traditional antioxidant therapy with low efficiency and large side effects. In the DKD mouse model, AAN can significantly protect renal tubular mitochondria, reduce tubular cell ferroptosis and lowered 24‐hour urine protein, serum creatinine, blood urea nitrogen, urine albumin/creatinine ratio, and kidney index in mice. It also restored the levels of proteins associated with kidney damage, effectively restoring kidney function, significantly reduce the infiltration of immune cells and the secretion of inflammatory factors and reduce EMT, ultimately achieving efficient treatment of DKD. Currently, the core therapies for diabetic kidney disease primarily include glycemic control, blood pressure management (renin‐angiotensin‐aldosterone system (RAAS) blockade), and sodium‐glucose cotransporter 2 (SGLT‐2) inhibitors, which have been proven effective in slowing the progression of DKD.^[^ ^43^ ^]^ Although current therapies can effectively protect the kidneys, their therapeutic effects are not ideal, and they lack targeted protective effects on the kidneys. This study addresses the limitations of current treatments by developing a nanomedicine with dual mechanisms of action: renal tubular mitochondrial targeting and antioxidant protection. This innovation provides a new therapeutic approach for the treatment of diabetic kidney disease. In conclusion, this study provides a new model for effectively repairing renal tubular mitochondria, which not only overcomes the bottleneck problems of frequent administration and poor effect of traditional antioxidants in the treatment of DKD, but also avoids the toxic side effects of antioxidants. Moreover, AAN has excellent biocompatibility and will show great advantages and prospects in the clinical transformation of the treatment of DKD.

Currently, the clinical translation of drugs faces multiple challenges, with drug toxicity/side effects and efficacy limitations being inevitable and critical aspects in preclinical trials. Drug toxicity and side effects serve as major barriers to clinical translation, leading to the elimination of numerous drug candidates during this phase. Drug efficacy, which largely determines the clinical applicability of a drug, serves as a key driver for further in‐depth research.^[^ ^44^ ^]^ Numerous studies have demonstrated that selenium‐containing nanoparticles exhibit remarkable therapeutic potential in various diseases, including osteoarthritis, Alzheimer's disease, gastrointestinal disorders, liver/kidney diseases, and cancer.^[^ ^45^, ^46^ ^]^ Given the renal protective effects and excellent biosafety profile of AAN, it holds promising prospects for clinical translation. However, several challenges remain to be addressed. First, animal models cannot fully replicate human responses, necessitating extensive experimental and clinical data for validation. Second, systematic pharmacokinetic studies are required to elucidate the absorption, distribution, metabolism, and excretion (ADME) of AAN in vivo.

## 4 Experimental Section

### Materials

Mouse TNF‐α (E‐EL‐M3063), IL‐6 (E‐EL‐M0044), IL‐1β (E‐EL‐M0037) ELISA kit were purchased from Elabscience Biotechnology (Houston, TX). Anti‐fluorescence bursting agent ProLong Glass Antifade Mountant purchased from Invitrogen. Creatinine (Cr) Assay kit (sarcosine oxidase)(C011‐2‐1), Urea Assay Kit (C013‐2‐1) and Total cholesterol assay kit (A111‐1‐1) were purchased from Nanjing Jiancheng Bioengineering Institute. Enhanced mitochondrial membrane potential assay kit with JC‐1(C2003S), Hoechst 33342 Staining Solution for Live Cells (C1028), and Enhanced ATP Assay Kit (S0027) were purchased from Beyotime Biotechnology. Fibronectin (bs‐4906R) mAb was purchased from BiossInc. Antibodies against Transferrin Receptor (TFR1, 381603), Vimentin (R22775) were obtainted from ZEN‐BIOSCIENCE. E‐cadherin (AF0131), α‐SMA (AF1032) mAb were bought from Affinity Biosciences Pty Ltd. Collagen I (14695‐1‐AP), GPX4 (67763‐1‐Ig), NGAL (30700‐1‐AP), KIM‐1 (83221‐2‐RR) and TGF‐β (21898‐1‐AP) antibodies were obtained from Proteintech Group Inc. Anti‐fluorescence quenching sealing liquid with DAPI (P36981), Goat anti‐Rabbit IgG (H + L) Highly Cross‐Adsorbed secondary antibody Alexa Fluor 488 (A11029), Goat anti‐Rabbit IgG (H + L) Cross‐Adsorbed secondary antibody Alexa Fluor 555 (A21428), and MitoSOX Red Mitochondrial Superoxide Indicator (M36008) were provided from Thermo Fisher Scientific. HRP‐conjugated Goat anti‐Mouse IgG (H+L) (AS003) and HRP‐conjugated Goat anti‐Rabbit IgG (H+L) (AS014) were purchased from Abclonal.

### Synthesis of AAN and AAN‐FITC

100 mg of L‐selenocysteine was added to 70 mL of ultrapure water, and then NaOH was added to adjust the pH to 9 to dissolve L‐selenocysteine, followed by stirring at 60 °C for 24 h. Subsequently, the precipitate was removed by centrifugation at 12 000 r for 10 min, and the supernatant was dialyzed for 24 h (with four water changes) to remove the unreacted impurities. Finally, the AAN was obtained by lyophilization.

10 mg of AAN and 12.5 mg of polyethylene glycol were added to 10 mL of Tris buffer solution and reacted for 12 h at room temperature. After that, the precipitate was dissolved in 8 mL of ultrapure water and then 2 mg of FITC (2 mL in DMSO) was added for 5 h at room temperature in the dark. Subsequently, unreacted FITC was removed by dialysis for 24 h (with four water changes). Finally, the FITC‐AAN were obtained by lyophilization.

### Characterization of AAN

TEM images were taken using a TECNAI G2 high‐resolution transmission electron microscope. XPS measurement (VG escalab MKII) was used to analyze the elemental valence state of AAN. Fluorescence spectra were determined using a F98 fluorescence spectrophotometer. UV / Vis spectra were collected with a Varian Cary 50 UV / VIS spectrophotometer. FTIR were taken by Bruker Vertex 7 Fourier Transform Infrared Spectrometer. The XRD measurements were carried out using a Bruker D8 Discover X‐ray Diffraction System.

### ROS Scavenging Capacity of AAN

The O_2_^·−^ scavenging ability of AAN was detected by nitroblue tetrazolium (NBT) method. In the presence of methionine, riboflavin can be reduced by light, and the reduced riboflavin can be easily reoxidized under aerobic conditions to produce O_2_^·−^, which can reduce yellow NBT to blue methyl hydrazone, which has maximum absorption at 560 nm. Briefly, different concentrations of AAN (0, 8, 16, 32, 64, 128 µg mL^−1^) were mixed with methionine (0.01 M), riboflavin (20 µm), NBT (0.01 M), PBS (0.1 M, pH 7.4), and deionized water. Then, the mixture was exposed to UV light for 5 min. Finally, the O_2_^·−^ scavenging ability of AAN can be analyzed by comparing the fluorescence intensity.

The ·OH scavenging efficiency of AAN was determined by fluorescence spectrophotometry. The Fenton reaction between FeSO_4_ and H_2_O_2_ catalyzes the formation of ·OH. Then ·OH binds nonfluorescent terephthalic acid (TA) and converts it to fluorescent 2‐hydroxy TA. The addition of AAN can reduce the production of the latter, thus reducing the fluorescence intensity. Briefly, different concentrations of AAN (0, 1, 2, 4, 8, 16 µg mL^−1^) were mixed with benzoic acid (0.1 mM), ferrous sulfate (0.05 mM), H_2_O_2_ (1 mM), and PBS (0.01 mM, pH 7.4). After standing for 6 min, the mixture was transferred to a cuvette, and the corresponding fluorescence intensity was scanned at an excitation wavelength of 320 nm.

The ·OH scavenging efficiency of AAN was determined by fluorescence spectrophotometry. The Fenton reaction between FeSO_4_ and H_2_O_2_ catalyzes the formation of ·OH. Then ·OH binds nonfluorescent terephthalic acid (TA) and converts it to fluorescent 2‐hydroxy TA. The addition of AAN can reduce the production of the latter, thus reducing the fluorescence intensity. Briefly, different concentrations of AAN (1, 2, 4, 8, 16, 32 µg mL^−1^) were mixed with benzoic acid (0.1 mM), ferrous sulfate (0.05 mM), H_2_O_2_ (1 mM), and PBS (10 mM), pH 7.4). After standing for 6 min, the mixture was transferred to a cuvette, and the corresponding fluorescence intensity was scanned at an excitation wavelength of 320 nm.

The scavenging ability of AAN to ONOO^−^ was detected by pyrogallol red method. Pyrogallol red has a specific absorption peak at 540 nm, and ONOO^−^ can quench pyrogallol red and reduce its specific absorption peak. 10 µL of pyrogallol red (5 mM), 9 µL of ONOO^−^ (1.73 mM) and different concentrations (0, 100, 200, 400 µg mL^−1^) of ONOO^−^ were mixed, with Pyrogallol Red solution and pyrogallol red containing ONOO^−^ as the Control. After 15 min of reaction, the absorbance of pyrogallol red at 540 nm was determined by UV/Vis spectrophotometry to determine the ONOO^−^ scavenging ability.

### Selenium Content Detection

At 6 h, 12 h, 24 h, 36 h, 48 h, 60 h, and 72 h, the content of Se in AAN with and without H_2_O_2_ was detected using Inductively Coupled Plasma Mass Spectrometry (ICP‐MS).

### Animal Experiment

C57 BL/6 mice (male, 8 weeks, 23–25 g) were purchased from Hunan STA Laboratory Animal CO., LTD (SYXK (XIANG) 2020‐0019). All animal research protocols were approved by the Institutional Animal Care and Use Committee (IACUC) of Xiangya Hospital, Central South University (ethics batch number 2024030586). These animals were fed with standard diet and water in a clean environment at 24 ± 2 °C and a 12 h light**/**dark cycle for 7 days. Then, 48 mice were fed with high‐fat diet (HFD, basic diet 63.5%, lard 8.0%, egg yolk powder 10.0%, sucrose 18.0%, sodium cholate 0.5%) for 4 weeks, and the remaining 12 mice were fed with normal diet. After that, mice fed with HFD were intraperitoneally injected with streptozotocin (STZ, sigma, 60 mg kg^−1^) daily for 5 days. After 72 h the last intraperitoneal injection, the mice were fasted for 12 h. Blood was collected from the tail vein, and fasting blood glucose of mice in each group was measured by a glucometer. If the fasting blood glucose value ≥ 11.1 mM and the model group is normal, it is considered that the diabetes modeling is successful.

12 healthy mice were selected as the normal Control group, and 48 model mice were randomly divided into four groups: 1) HFD+STZ model group, 2) HFD+STZ+AAN (5 mg kg^−1^), 3) HFD+STZ+AAN (10 mg kg^−1^), 4) HFD+STZ + Metformin (200 mg kg^−1^), with 12 mice in each group. Among them, AAN was given by tail vein injection every 4 days, and Metformin was given by gavage once a day for 6 weeks. The weight and fasting blood glucose of mice were tested every week. At the sixth week, the urine protein / creatinine value of the model group was significantly higher than that of the normal Control group, reaching about 15 times. The DKD model was successfully constructed. Blood was collected after anesthesia, and tissues were collected as soon as possible, weighed, and stored. Some tissues were immediately fixed in 4% universal tissue fixative, and some tissues were immediately frozen in liquid nitrogen and stored at ‐80 °C. The antidiabetic kidney disease effect was confirmed by three repeated animal experiments. The results presented are a representative experiment of 6–12 mice in each group.

### Biodistribution and Metabolism of AAN In Vivo

Both normal mice and DKD mice were injected with AAN‐FITC through the tail vein. Wait for 9 h, collect the heart, liver, spleen, kidney, and place them under a stereo fluorescence microscope (Leica, M205FCA) for observation and image acquisition.

Both normal mice and DKD mice were injected with AAN‐FITC through the tail vein. After waiting for 0, 1, 3, 6, 9, 12, 24, 48, 72, 96 h, the kidneys were collected and placed under a stereo fluorescence microscope (Leica, M205FCA) for observation and image acquisition.

### Hematoxylin and Eosin (H&E) Staining

After AAN treatment, kidneys were fixed in 4% universal tissue fixative for 24 h, embedded in paraffin, and then cut into sections (5 µm). Dewaxed after baking at 65 °C for 1h, and dehydrated in different concentrations of ethanol. Next, the sections were stained with hematoxylin and eosin dyes. Finally, the sections were observed under a microscope and photographed.

### Dihydroethidine (DHE) Staining

Kidney tissues were embedded in Optimal Cutting Temperature (OCT) compound and then frozen. Kidneys were cut into ≈5 µm sections and mounted on cationic anti detachment slides. The superoxide anion fluorescent probe DHE was added to the tissue sections and incubated in the dark for 30 min at room temperature. The sections were washed three times with 0.01 M PBS to remove excess dye. The sections were then sealed with anti‐fluorescence quenching sheets containing DAPI. The samples were observed and photographed under a fluorescence microscope, and the fluorescence intensity was analyzed using image J software.

### Immunofluorescence Analysis

Paraffin sections of 5 µm thick kidney tissue were prepared and dewaxed with dewaxing agent for 3 min and 3 times. The slides were gradually rehydrated in various concentrations of ethanol and washed with deionized water. To expose antigens, kidney sections were bathed in target repair solution (0.01 M sodium citrate, pH 6.0) in a 95 °C water bath for 40 min, and then washed with PBS. 0.1% Triton X‐100 was permeabilized for 10 min at room temperature, 10% goat serum solution was incubated for 60 min at room temperature, and then washed three times with PBS. Collagen I antibody (diluted in 10% goat serum) was used to cover the surface of the sections and incubated overnight at 4 °C. The sections were equilibrated to room temperature and incubated with Alexa fluor‐488‐conjugated Goat anti mouse antibody in a wet box for 1 h at 37 °C. Nuclei were stained drop by drop with DAPI and incubated for 10 min at room temperature and sealed with the anti‐fluorescent bursting agent prolong glass antifade mountant (Invitrogen). Tissue sections were then imaged using a Leica confocal laser scanning microscope (CLSM) Sp8 inverted confocal microscope under excitation of three separate laser lines (488 nm).

### Transmission Electron Microscopy (TEM) of Kidney

The kidney tissues were fixed in fresh electron microscopy fixative and then washed three times with 0.1 M PBS (pH 7.4) for 15 min each. Tissues were fixed in 1% OsO_4_ in 0.1 M PBS (pH 7.4) for 2 h at room temperature, protected from light. After removing OsO_4_, the tissue was rinsed three times for 15min in 0.1 M PBS (pH 7.4). After dehydration at room temperature, the tissue undergoes resin osmotic embedding and polymerization. The resin block was cut into 60–80 nm thin sections on an ultramicrotome, and the tissue was fished onto a 150 mesh copper grid with Formvar film. First, it was stained with 2% uranyl acetate in saturated ethanol solution for 8 min in the dark, rinsed with 70% ethanol for 3 times, and then rinsed with ultrapure water for 3 times. Next, 2.6% lead citrate avoided CO_2_ staining for 8 min, and then rinsed with ultrapure water for 3 times. After drying with filter paper, the copper grid was put into the grid plate and dried overnight at room temperature. Finally, the copper grid was observed and images were taken under TEM (Hitachi HT7800/HT77000).

### Cell Culture

HK‐2 cells were cultured in DMEM/F‐12 medium supplemented with 10% calf serum at 37 °C in an incubator provided with a humidified atmosphere of 5% CO_2_. Raw 264.7 cells were cultured in DMEM medium supplemented with 10% fetal bovine serum (FBS) and 1% penicillin streptomycin at 37 °C in an incubator provided with a humidified atmosphere of 5% CO_2_.

### Assessment of Drug Toxicity to Cells

HK‐2 cells were seeded into 96‐well plates at 1×10^4^ cells per well and treated with different concentrations of AAN and Fer‐1. Test for 1 h at 37 °C using CCK‐8 reagent at 10 µL per well. Optical density (OD) values at 450 nm were detected using a microplate reader.

RAW264.7 cells were seeded into 96‐well plates at 2 × 10^5^ per well and treated with different concentrations of AAN and Fer‐1. Test at 37 °C using CCK‐8 reagent at 10 µL per well for 1 h. OD values at 450 nm were detected using a microplate reader.

### Western Blotting

Proteins were isolated from kidney tissue supplemented with RIPA mixture (RIPA, protease inhibitors, and phosphatase inhibitors, 100:1:1) by tissue homogenizer. For cellular protein isolation, the cell pellet is collected, and protein isolation is performed using RIPA mixture (RIPA, protease inhibitors, and phosphatase inhibitors, 100:1:1). Protein concentration was measured using a BCA kit. 30 µg of total protein was taken and separated by 8%‐12% SDS‐PAGE gel electrophoresis, and after separation, the protein was transferred to a PVDF membrane (transfer, determine the transfer current and transfer time based on the molecular weight of the target protein.). Blocking the membrane with 5% milk for 1 h at room temperature. Incubate overnight at 4 °C with a specific primary antibody. Then, wash the membrane three times with TBST for 5–10 min each, and then incubate with the corresponding secondary antibody for 1 h at room temperature. Then, wash the membrane three times with TBST for 5–10 min each. We then visualized the bands using a gel recording system (Bio‐Rad, USA) and quantified them using Image Lab software.

### Mitochondrial Testing

The different treatments of live HK‐2 cells were incubated with MitoSOX reagent for 10 min at 37 °C, then stained with Hoechst 33342 live cell staining solution, and finally placed in warm buffer. Measure the mitochondrial membrane potential using JC‐1 staining working solution for 15 min at 37 °C. After incubation, the cells are washed several times and visualized using a fluorescence microscope.

### ATP Assay

Harvest HK‐2 cells with different treatments by centrifugation (14 000 g, 4 °C, 2 min) and wash with pre‐chilled PBS. ATP content is determined using an enhanced ATP assay kit. The detection solution is added to the opaque 96‐well plate and incubated for 5 min at room temperature. The supernatant of the lysed cells is then quickly mixed with the assay solution, and after 30 min, the values are read under a multimode microplate reader.

### Biochemical Analysis

Mice kidneys collected from each group are cryopreserved in a freezer at −80 °C and kidney homogenates are obtained for different experiments. TNF‐α, IL‐6 and IL‐1β levels were measured in kidney tissue homogenates by Enzyme‐Linked ImmunoSorbent Assay (ELISA) kit according to the manufacturer's protocol. MDA, GSH, and iron content determination kits are used according to the instructions.

Serum is obtained by centrifugation of mouse blood collected from each group and stored in a freezer at ‐80 °C. Use according to the test protocol of blood creatinine, blood urea nitrogen, and blood cholesterol (micromethod) kit.

### Drug Toxicity Testing In Vivo

Healthy C57BL/6 mice are randomly divided into two groups. The Control group consisted of healthy mice injected with 100 µL of 1x PBS (n = 6); The AAN group consists of healthy mice injected with 100 µL of AAN 10 mg kg^−1^ in 1x PBS (n = 6). Healthy mice are sacrificed 28 days after injection. Histological changes in mouse organs are assessed by H&E staining to assess long‐term toxicity of AAN.

Healthy C57BL/6 mice are randomly divided into two groups. The Control group consisted of healthy mice injected with 100 µL of 1x PBS (n = 6); The AAN group consists of healthy mice injected with 100 µL of AAN 20 mg kg^−1^ in 1x PBS (n = 6). Healthy mice are sacrificed 24 h after injection. Histological changes in mouse organs are assessed by H&E staining to assess short‐term toxicity of AAN.

Whole blood is used for hematological analysis with the following parameters: RBC, HGB, WBC, PLT. Indicators of liver function (ALT and AST) and renal function (BUN and CRE) were analyzed using an automated biochemistry analyzer BS‐2000 M.

### Data Analysis

All data in this study are all quantitative data, which are expressed using the mean ± standard deviation (SD), and no experimental data points are deleted. All in vitro experiments were biologically repeated three times (n = 3), and in vivo data were obtained from at least three independent experiments (n ≥ 3 animals per group: efficacy evaluation experiment, n = 6; Mechanism exploration experiment, n ≥ 3). For statistical comparison between two groups, independent samples t‐test (two‐sided) was used. For comparisons between multiple groups, one‐way ANOVA followed by post hoc multiple comparisons with SNK test was used. The significance level (α) was set at 0.05, and *P* values less than 0.05 were considered statistically significant. The assumption of homogeneity of variance was verified using Levene test. All statistical analyses were performed using image J (version 1.8.0), graphpad prism software version 8.0 (graphpad prism, San Diego, USA) and SPSS 23.0 software.

## Conflict of Interest

The authors declare no conflict of interest.

## Supporting information



Supporting Information

## Data Availability

The data that support the findings of this study are available from the corresponding author upon reasonable request.
