# Supplementary material for: Adaptive Antioxidant Nanomedicines Inhibit Ferroptosis in Renal Tubular Epithelial Cells to Alleviate Diabetic Kidney Disease
Source: Adv Sci (Weinh). 2025 Jul 6;12(37):e05168. doi: 10.1002/advs.202505168 (PMC12499382; doi:10.1002/advs.202505168)
Supplement: Supplementary file 1 — Supporting Information [file ADVS-12-e05168-s001.docx]

**Supporting Information**

**Adaptive Antioxidant Nanomedicines Inhibit Ferroptosis in Renal Tubular Epithelial Cells to Alleviate Diabetic Kidney Disease**

Zerun Liu^1,2#^, Ting Huang^1,2#^, Ying Hong^1,2#^, Yuqi Yang^1,2^, Wensheng Chen^1,2^, Qingtao Zeng^1,2^, Qiaohui Chen^3,4^, Yongqi Yang^3,4^, Xiaohong Ying^3,4^, Wan Zeng^3,4^, Ziyu Wu^3,4^, Tianjiao Zhao^3,4^, Xuesi Wan^5^, Jianlin Chen^6^, Kelong Ai^3,4,7^*, Qiong Huang^1,2^*

^1^ Department of Pharmacy, Xiangya Hospital, Central South University, Changsha, 410008, China.

^2^ National Clinical Research Center for Geriatric Disorders, Xiangya Hospital, Central South University, Changsha, 410008, China.

^3^ Xiangya School of Pharmaceutical Sciences, Central South University, Changsha, 410078, China

^4^ Hunan Provincial Key Laboratory of Cardiovascular Research, Xiangya School of Pharmaceutical Sciences, Central South University, Changsha, 410013, China.

^5^ Department of Endocrinology and Diabetes Center, The First Affiliated Hospital of Sun Yat-sen University, Guangzhou, Guangdong, PR China.

^6^ Pancreatic Surgery, Xiangya Hospital, Central South University, Changsha, 410008 China

^7^ Key Laboratory of Aging-related Bone and Joint Diseases Prevention and Treatment, Ministry of Education, Xiangya Hospital, Central South University, Changsha, 410008, China.

^#^ The authors Zerun Liu, Ting Huang and Ying Hong contributed equally to the work.

***Corresponding authors**: aikelong@csu.edu.cn (Prof. Kelong Ai), qionghuang@csu.edu.cn (Prof. Qiong Huang)


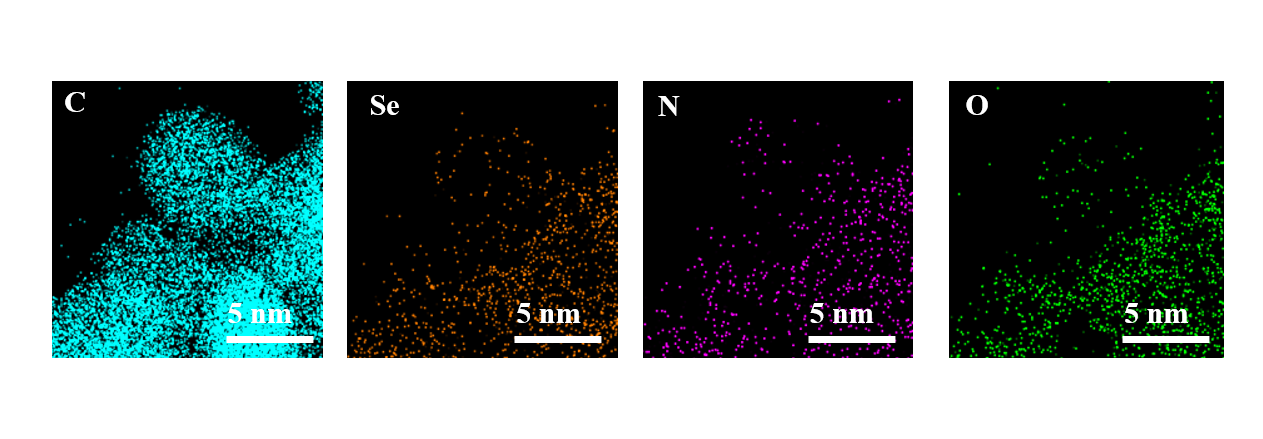


**Figure S1.** STEM-EDS of AAN. green, red, orange, and blue represent oxygen, nitrogen, selenium and carbon, respectively, Scale bar: 5 nm.


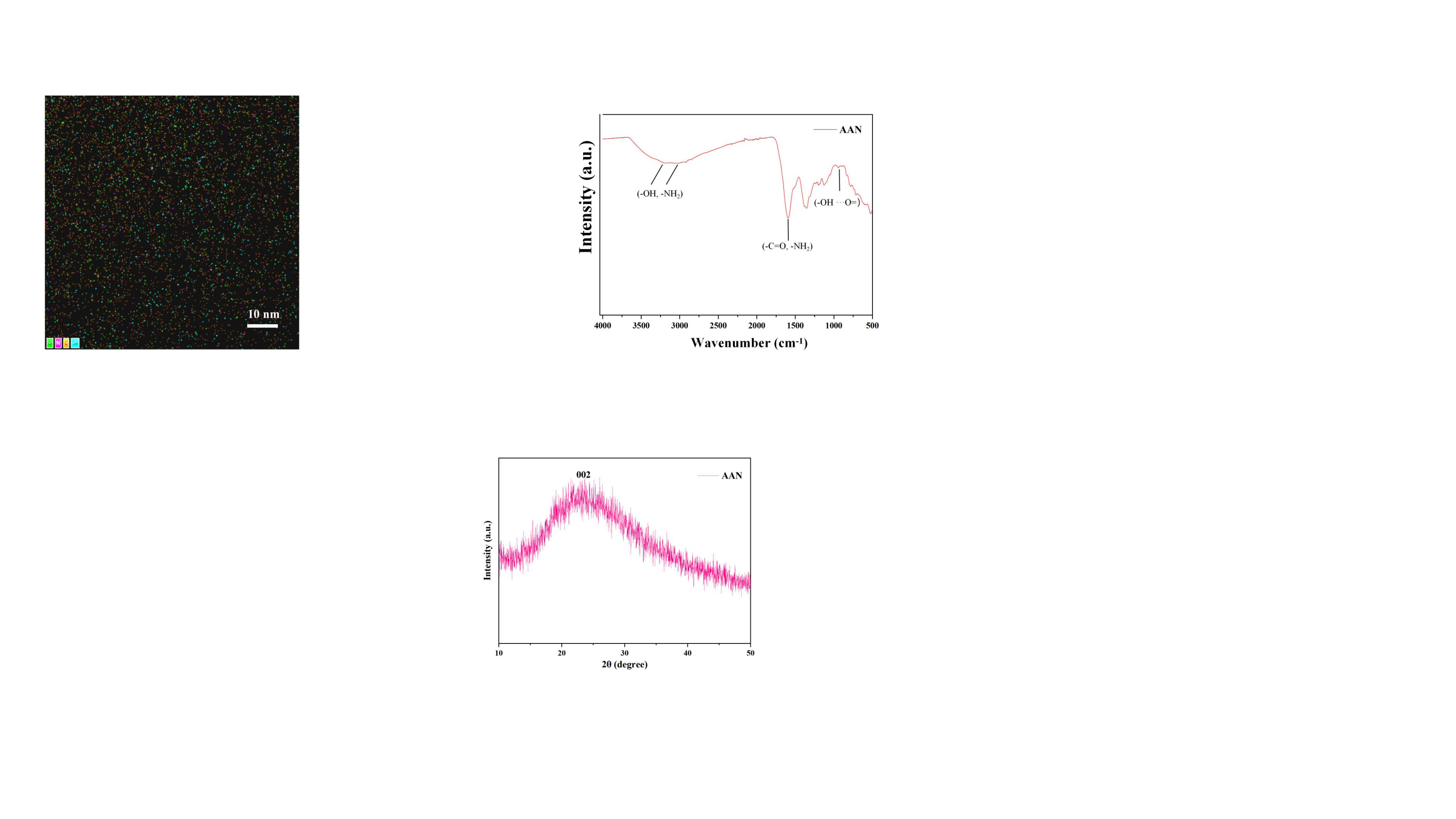


**Figure S2.** FTIR spectra of AAN.


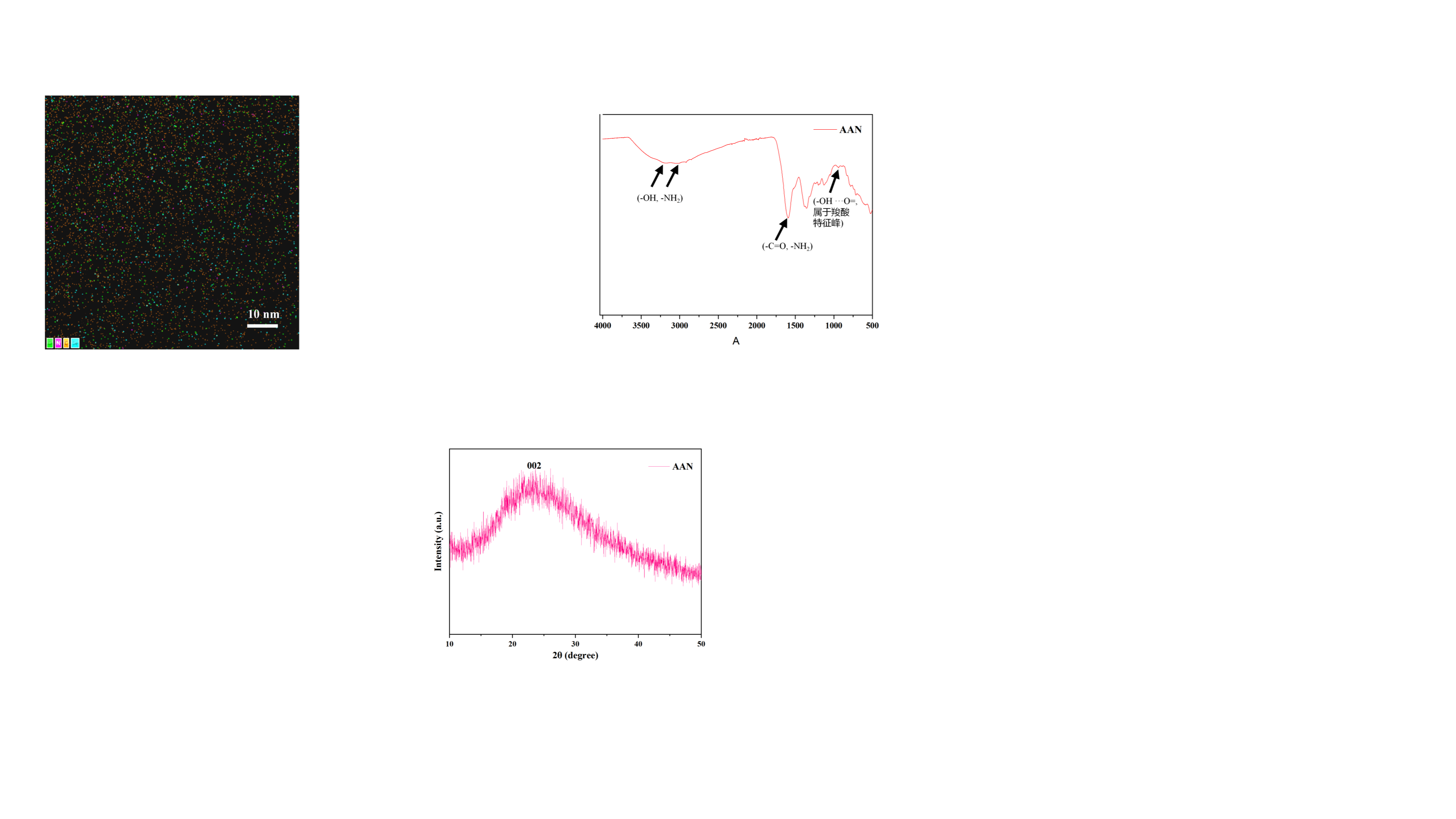


**Figure S3.** XRD of AAN.

**
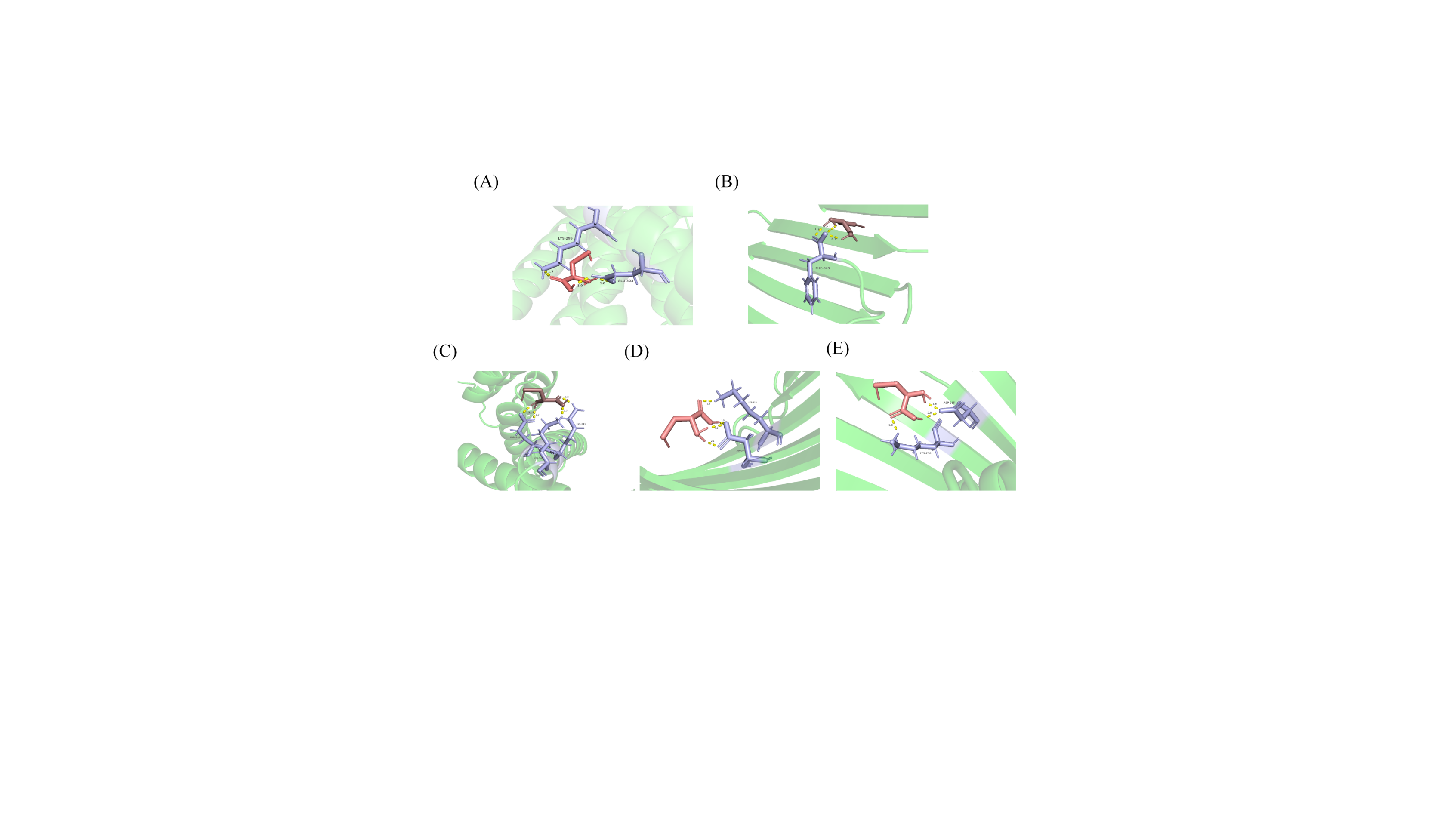
**

**Figure S4.** Molecular docking results of AAN with TOM34, TOM40, TOM70, VADC1, VADC2.


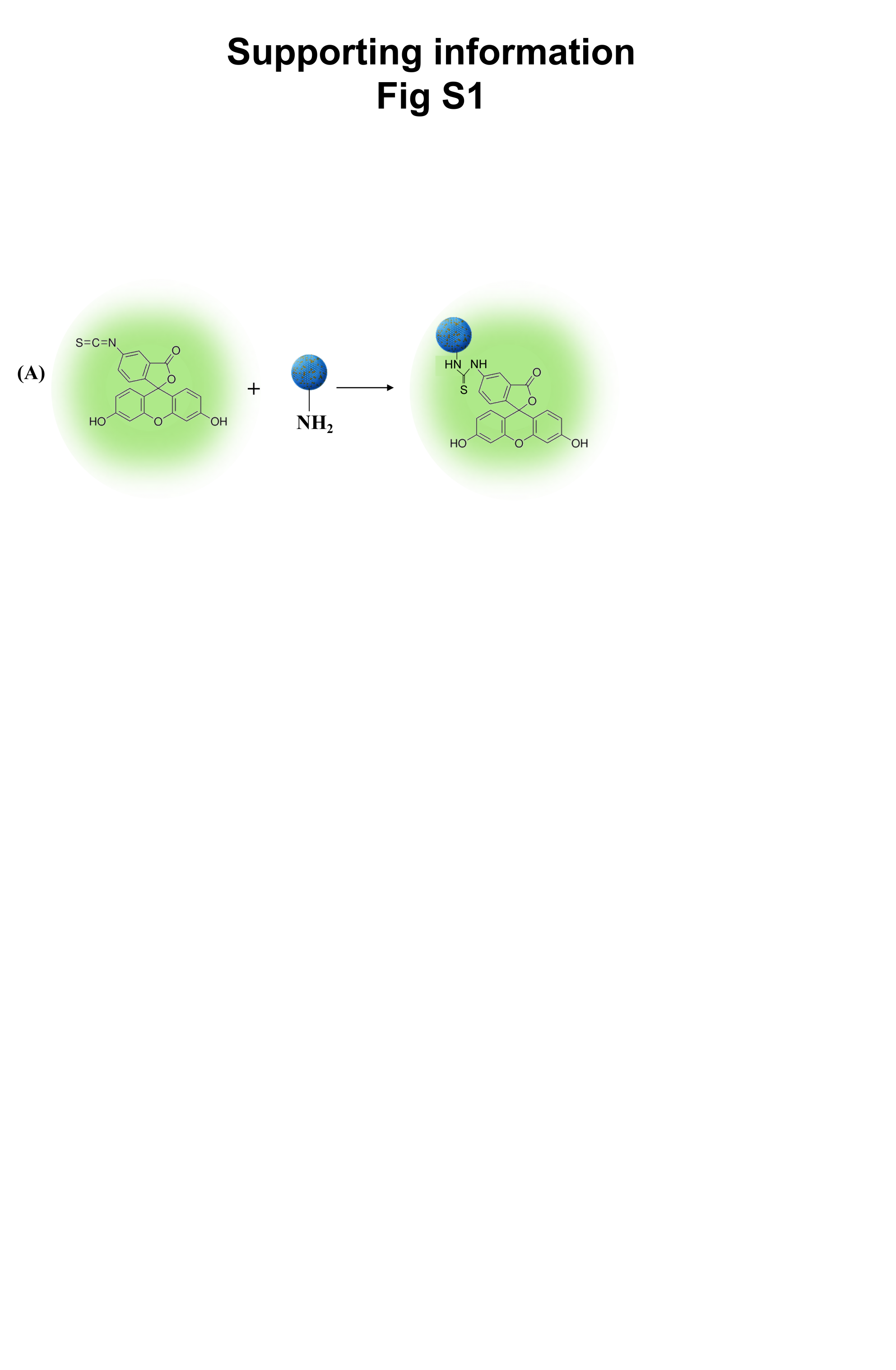


**Figure S5.** Synthesis of AAN-FITC.


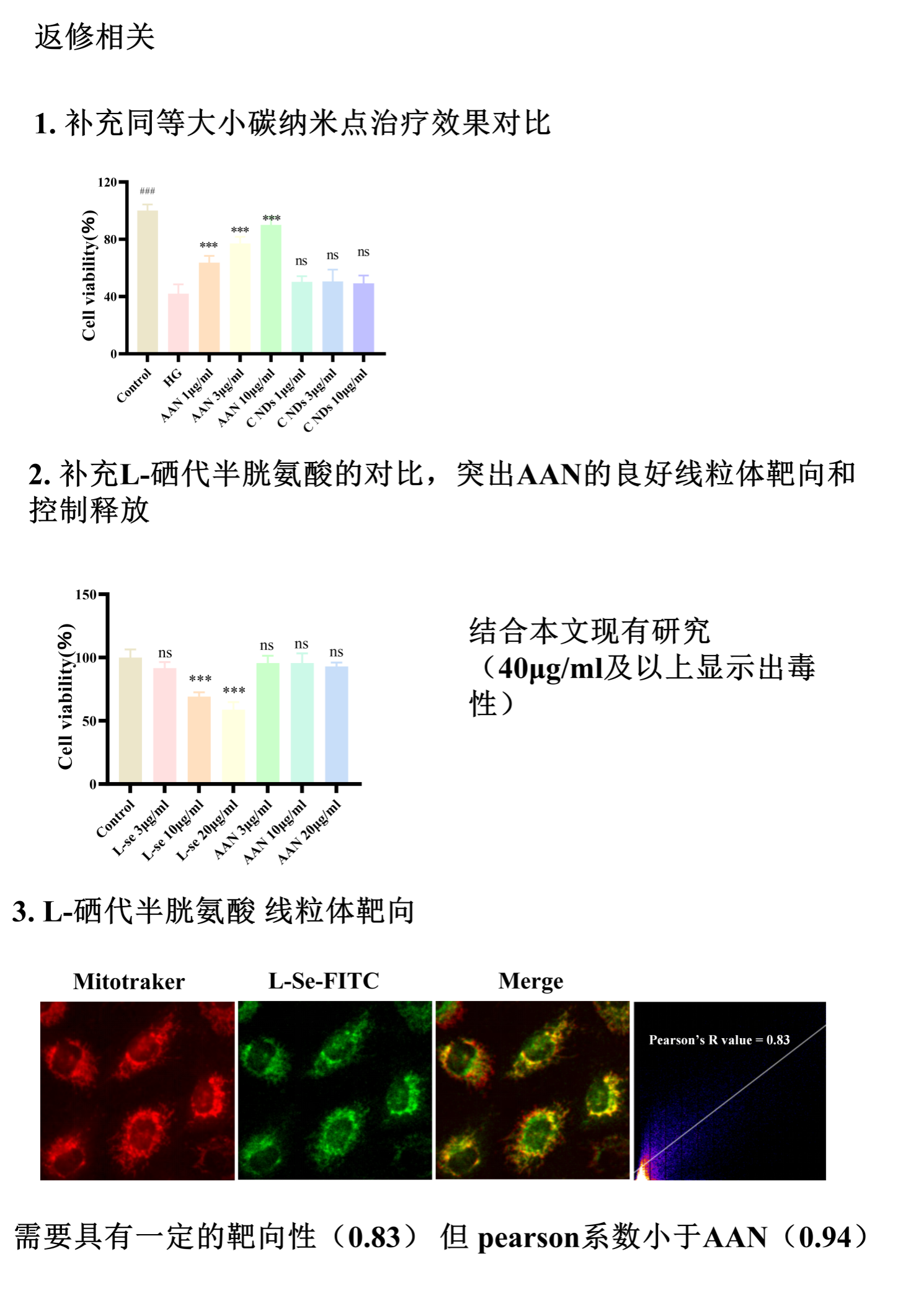


**Figure S6.** Mitotracker-stained with L-Se-FITC colocalization and Pearson coefficient in HK-2 cells, EX/EM=579/599nm.


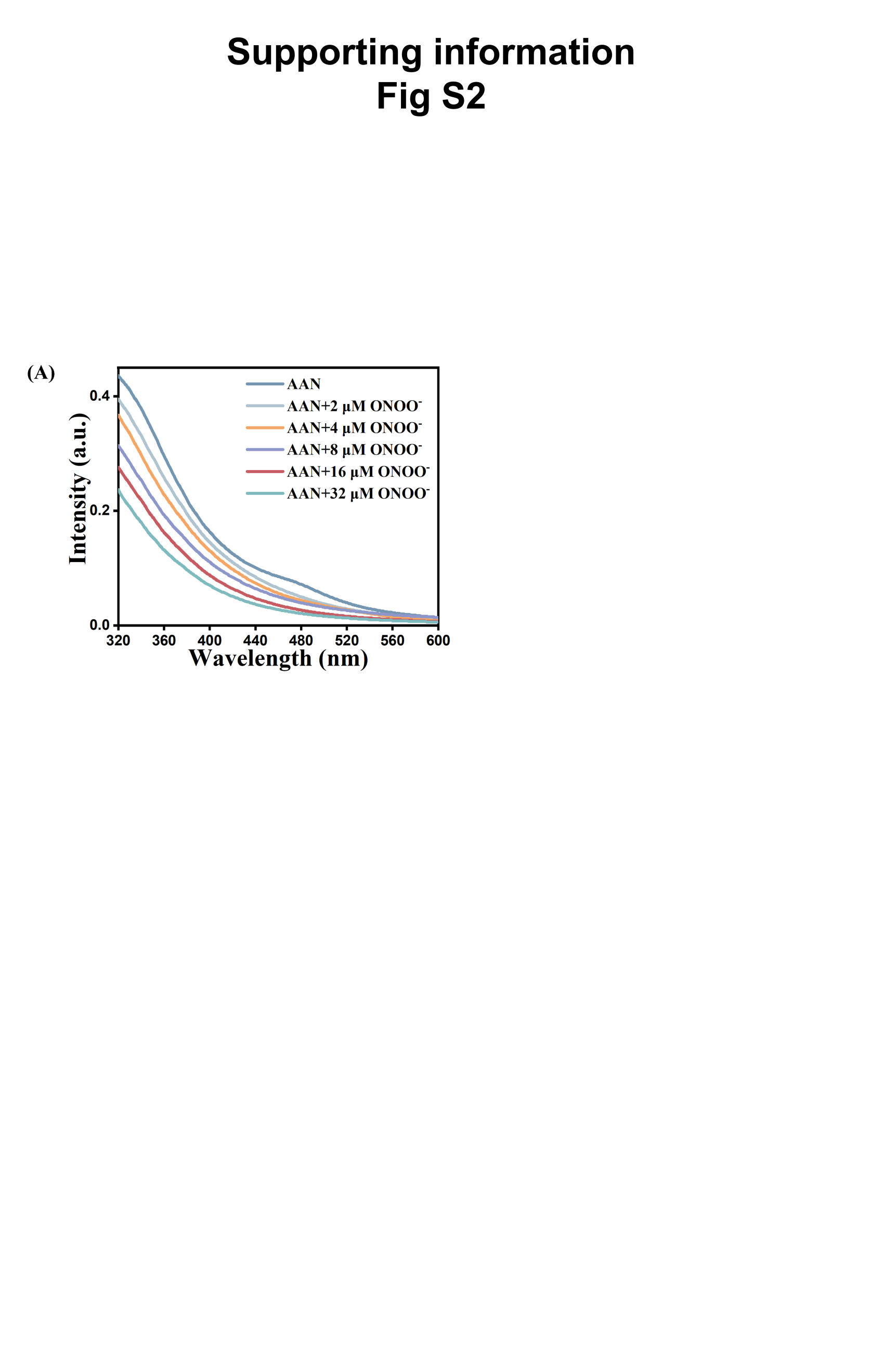


**Figure S7.** The ability of AAN to eliminate different concentrations of ONOO^-^.


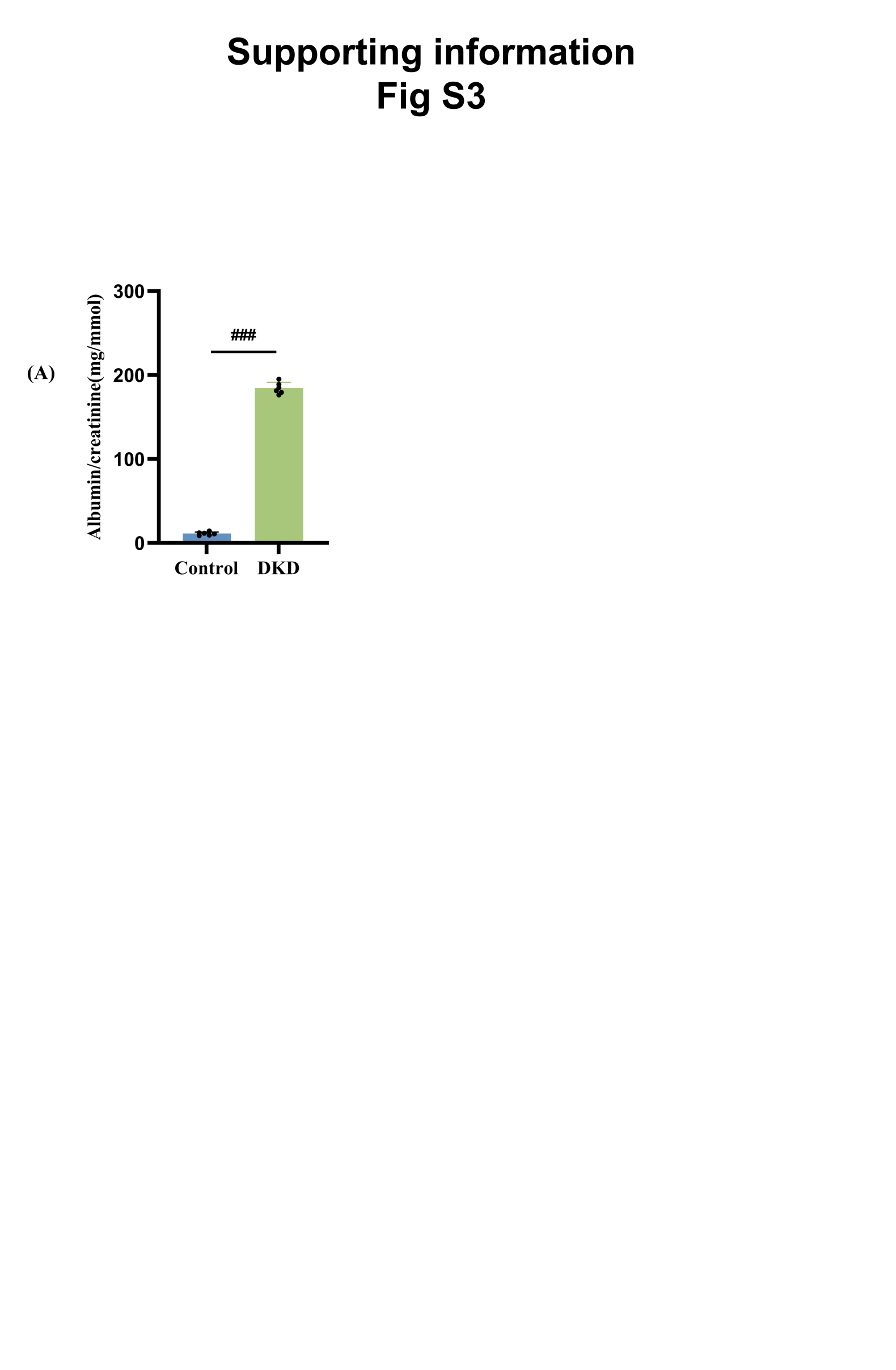


**Figure S8.** Albumin / creatinine of mice in Control and DKD groups. *^###^P* < 0.001 vs Control group.


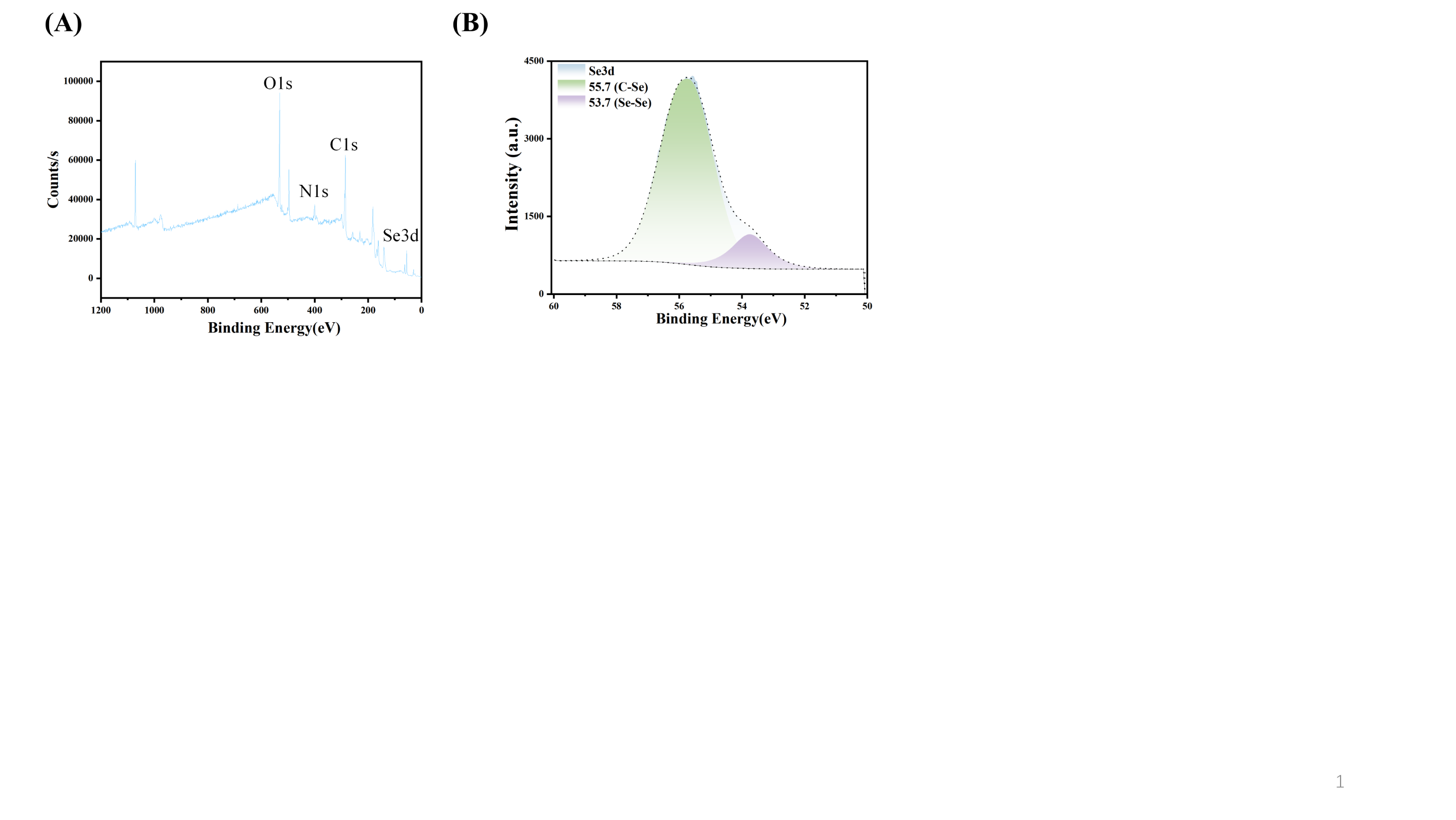


**Figure S9.** XPS spectrum of AAN in Urine (A), Se3d (B).


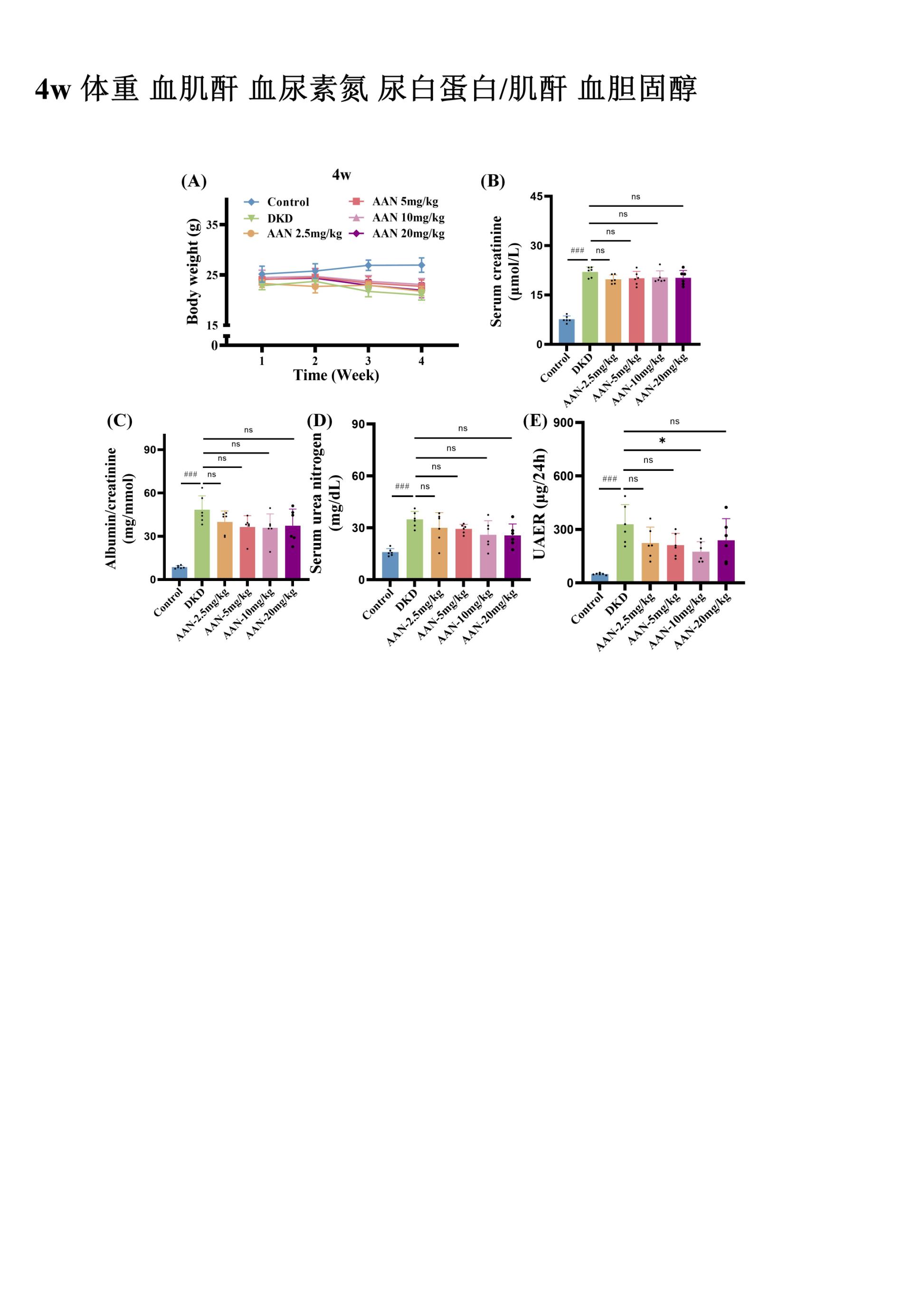


**Figure S10.** Preliminary experiment results showing the effects of various concentrations of AAN on mice's weight (A), serum creatinine (B), blood urea nitrogen (C), urinary albumin/creatinine ratio (D), and UAER (E) after 4 weeks of treatment. Data represent means ± S.D. from six independent replicates. *^###^P* < 0.001 vs Control group, **P* < 0.05 vs DKD group. ns means *P* > 0.05.


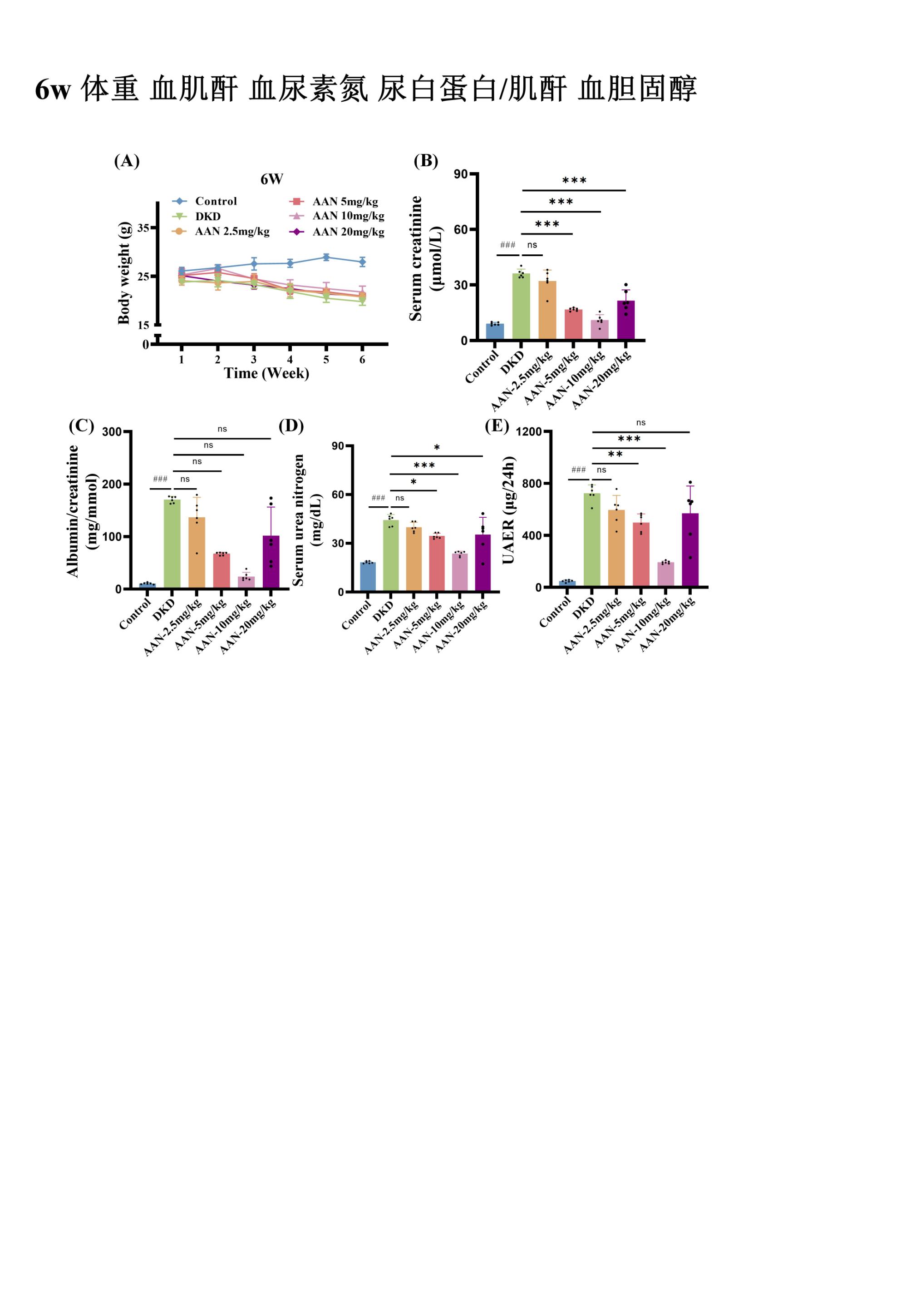


**Figure S11.** Preliminary experiment results showing the effects of various concentrations of AAN on mice's weight (A), serum creatinine (B), blood urea nitrogen (C), urinary albumin/creatinine ratio (D), and UAER (E) after 6 weeks of treatment. Data represent means ± S.D. from six independent replicates. *^###^P* < 0.001 vs Control group, **P* < 0.05, ***P* < 0.01, ****P* < 0.001 vs DKD group; ns means *P* > 0.05.


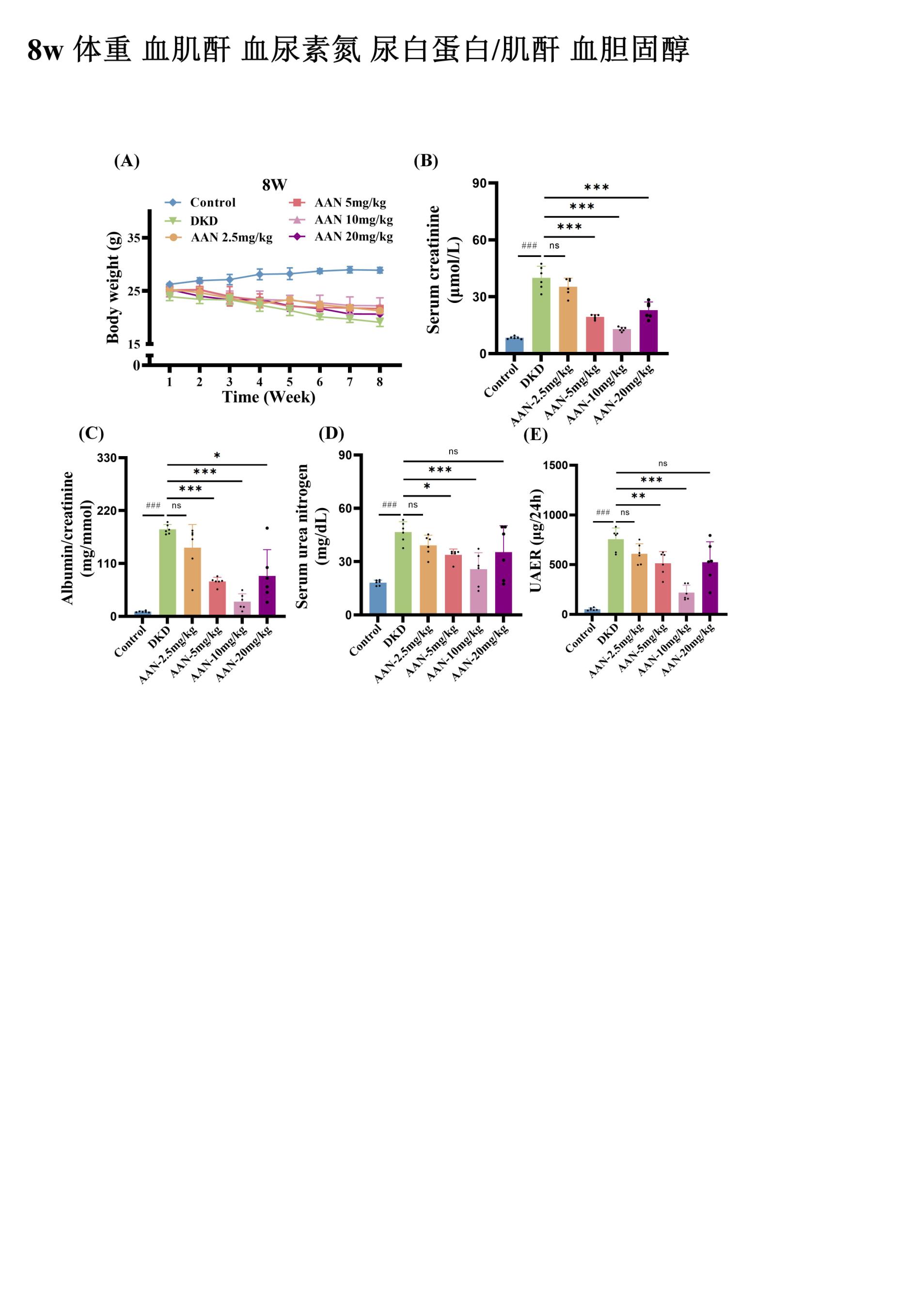


**Figure S12.** Preliminary experiment results showing the effects of various concentrations of AAN on mice's weight (A), serum creatinine (B), blood urea nitrogen (C), urinary albumin/creatinine ratio (D), and UAER (E) after 8 weeks of treatment. Data represent means ± S.D. from six independent replicates. *^###^P* < 0.001 vs Control group, **P* < 0.05, ***P* < 0.01, ****P* < 0.001 vs DKD group, ns means *P* > 0.05.


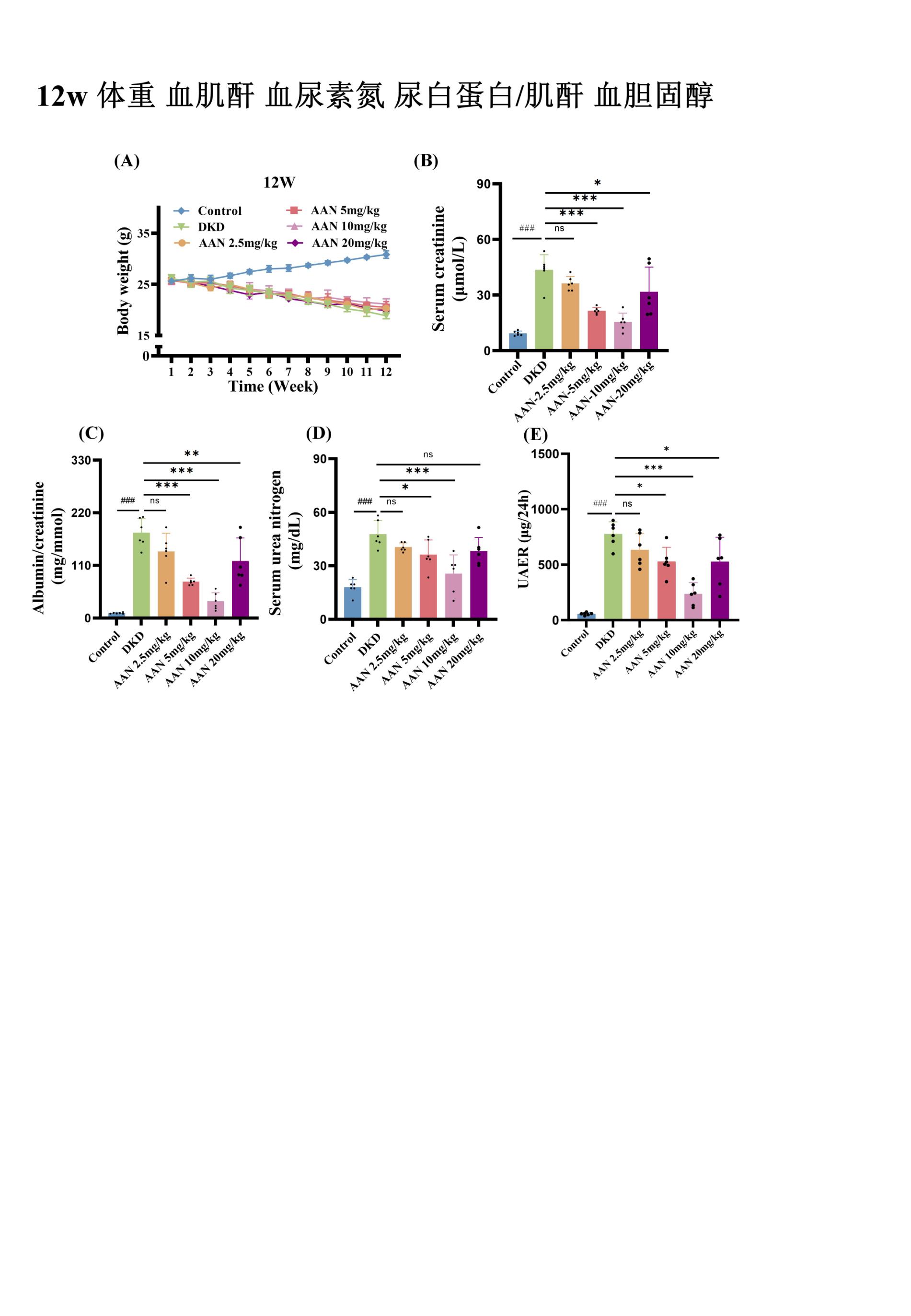


**Figure S13.** Preliminary experiment results showing the effects of various concentrations of AAN on mice's weight (A), serum creatinine (B), blood urea nitrogen (C), urinary albumin/creatinine ratio (D), and UAER (E) after 12 weeks of treatment. Data represent means ± S.D. from six independent replicates. *^###^P* < 0.001 vs Control group, **P* < 0.05, ****P* < 0.001 vs DKD group; ns means *P* > 0.05.


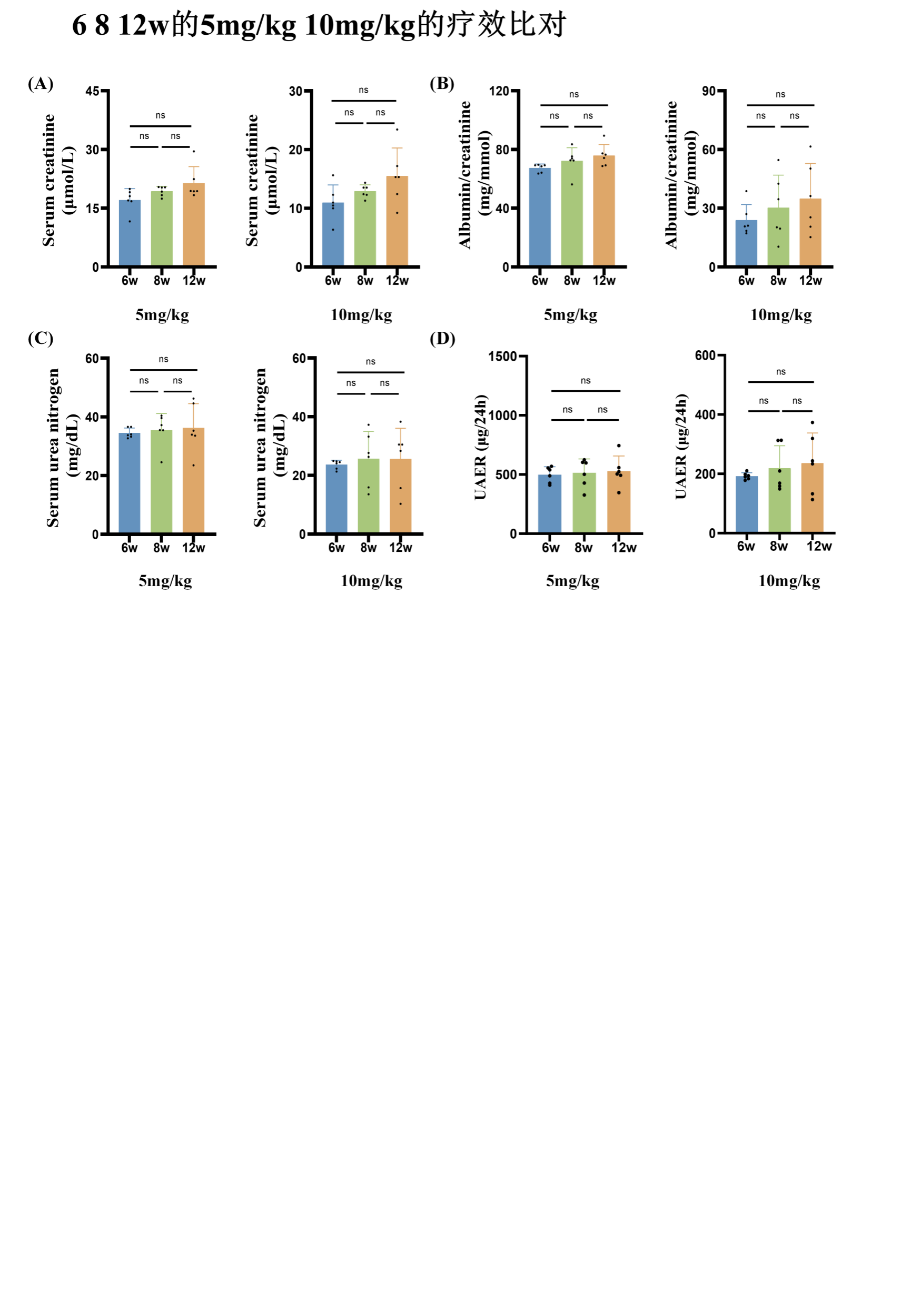


**Figure S14.** Comparison of the effect of different doses of drug treatment on serum creatinine (A), urinary albumin/creatinine ratio (B), serum urea nitrogen (C), and UAER (E) at 6, 8 and 12weeks. Data represent means ± S.D. from six independent replicates. ns means *P* > 0.05.


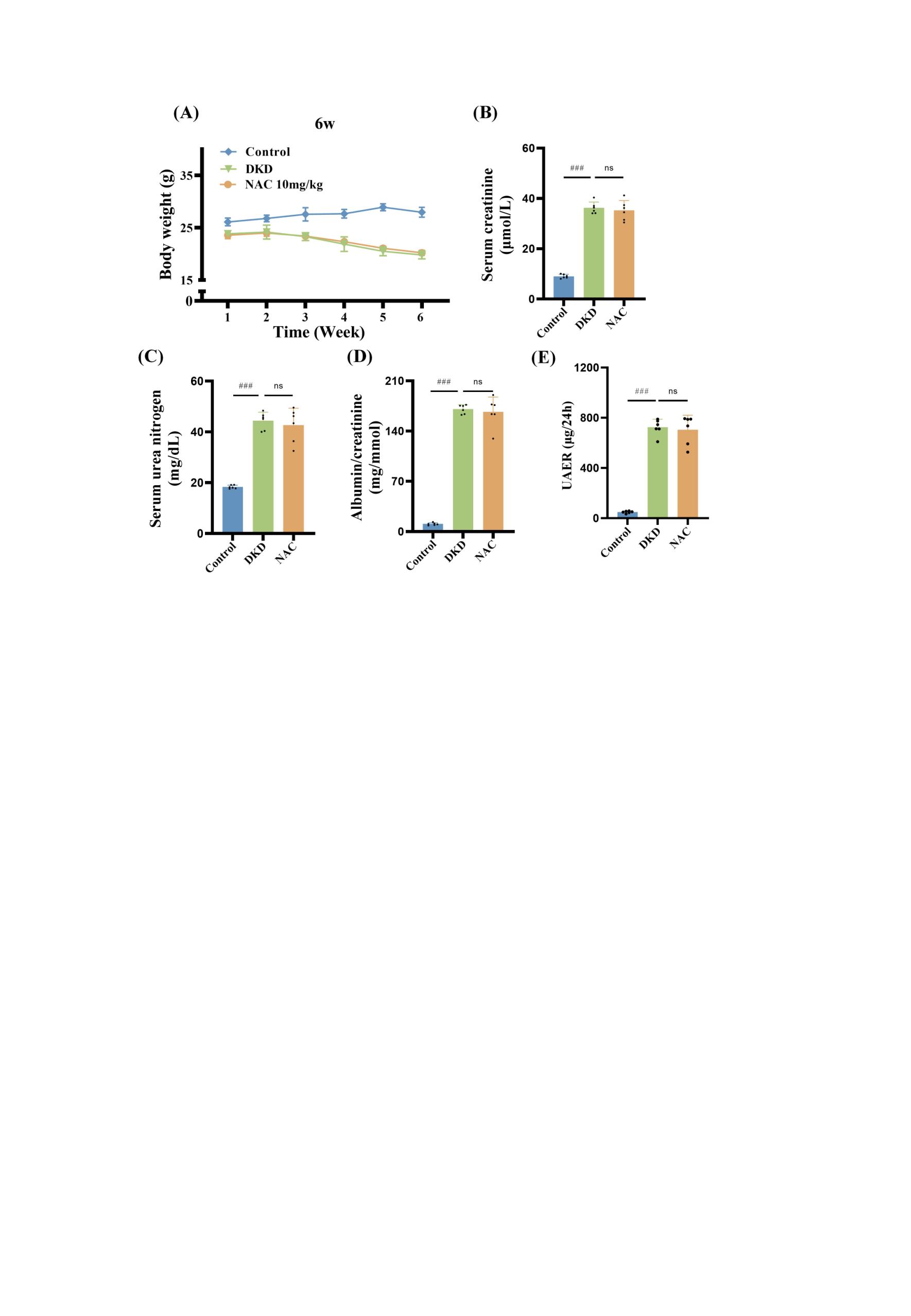


**Figure S15.** The effects of NAC on mice's weight (A), serum creatinine (B), blood urea nitrogen (C), urinary albumin/creatinine ratio (D), and UAER (E) after 12 weeks of treatment. Data represent means ± S.D. from six independent replicates. *^###^P* < 0.001 vs Control group, ns means *P* > 0.05.


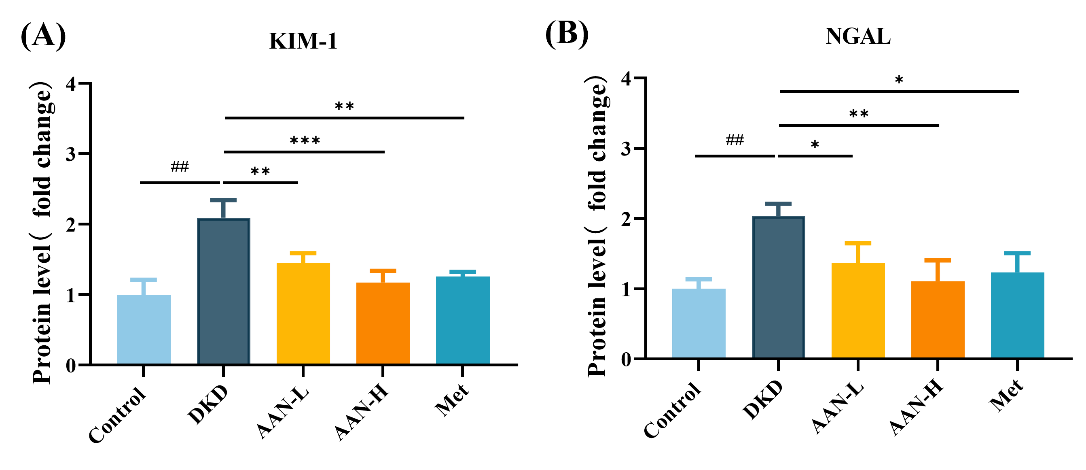


**Figure S16.** Quantitative analysis of KIM-1 (A) and NGAL (B) proteins in kidney tissues of mice in different groups. *^##^P* < 0.01 vs Control group, **P* < 0.05, ***P* < 0.01, ****P* < 0.001 vs DKD group.


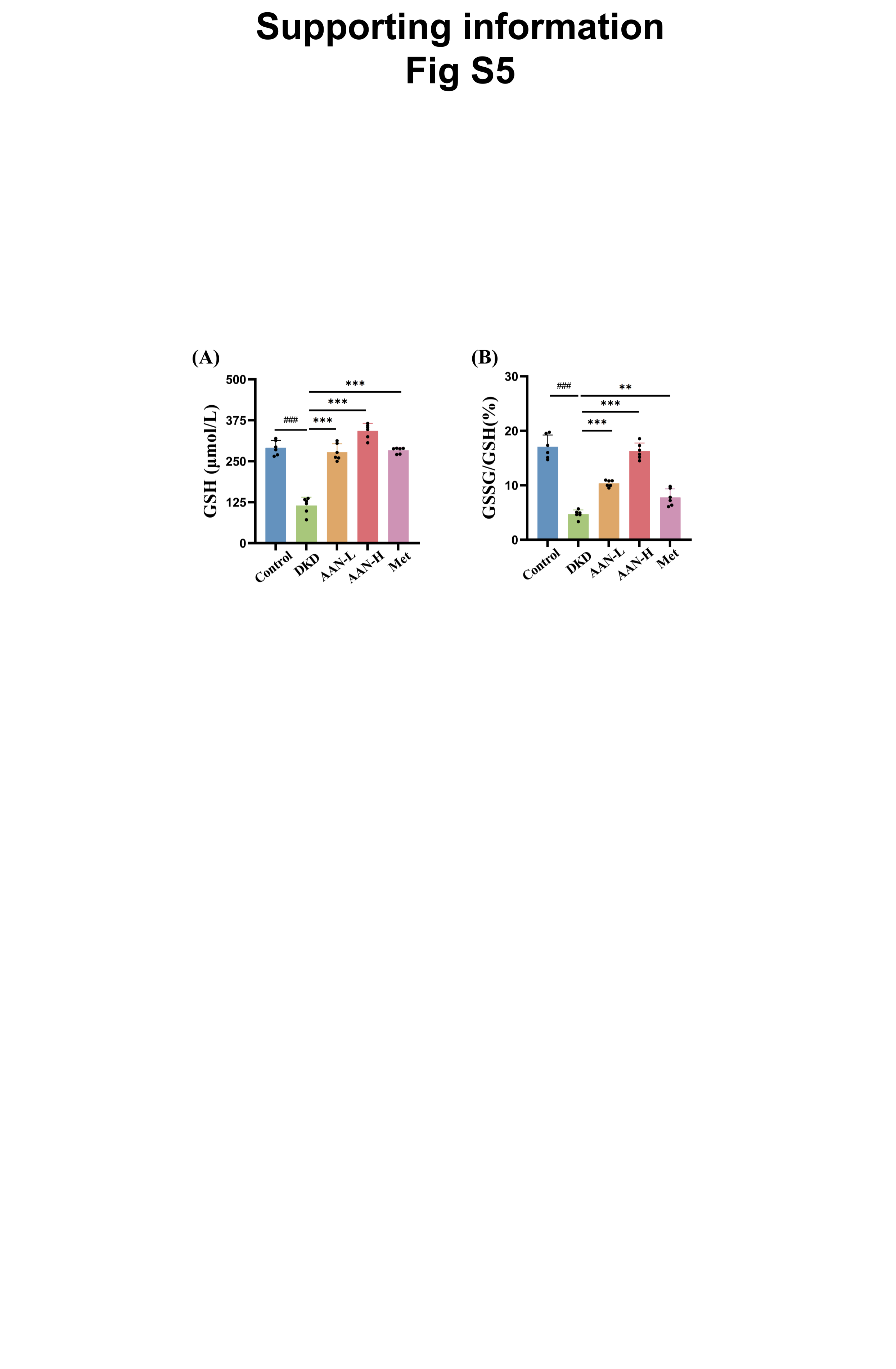


**Figure S17**. GSH content (A) and GSSG/GSH (B) ratio in kidney tissues of mice in different groups. *^###^P* < 0.01 vs Control group, ***P* < 0.01, ****P* < 0.001 vs DKD group.


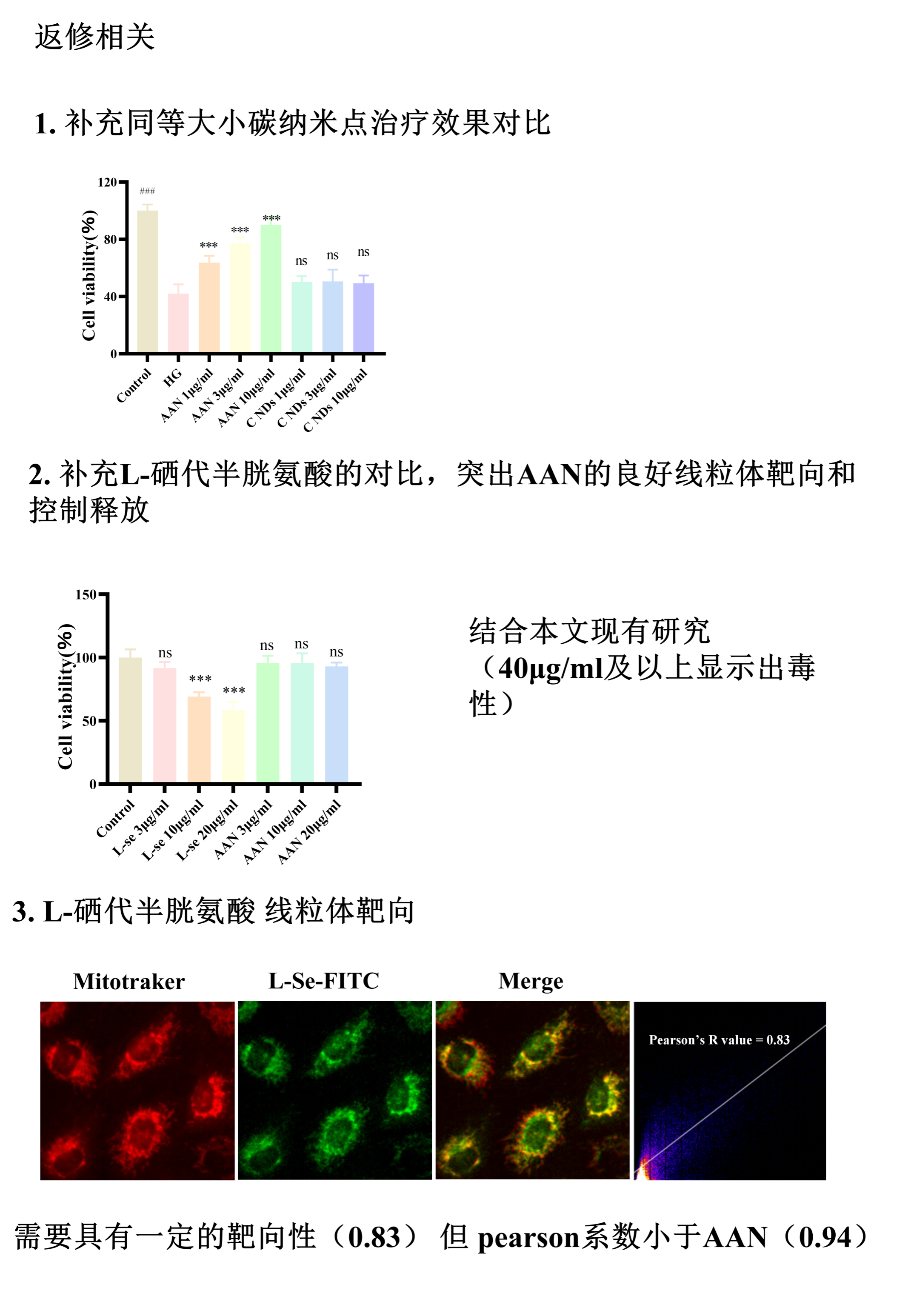


**Figure S18**. Cell viability of HK-2 cells induced by HG after treatment with different concentrations of AAN and C NDs. *^###^P* < 0.001 vs Control group, ****P* < 0.001 vs HG group, ns means *P* > 0.05.


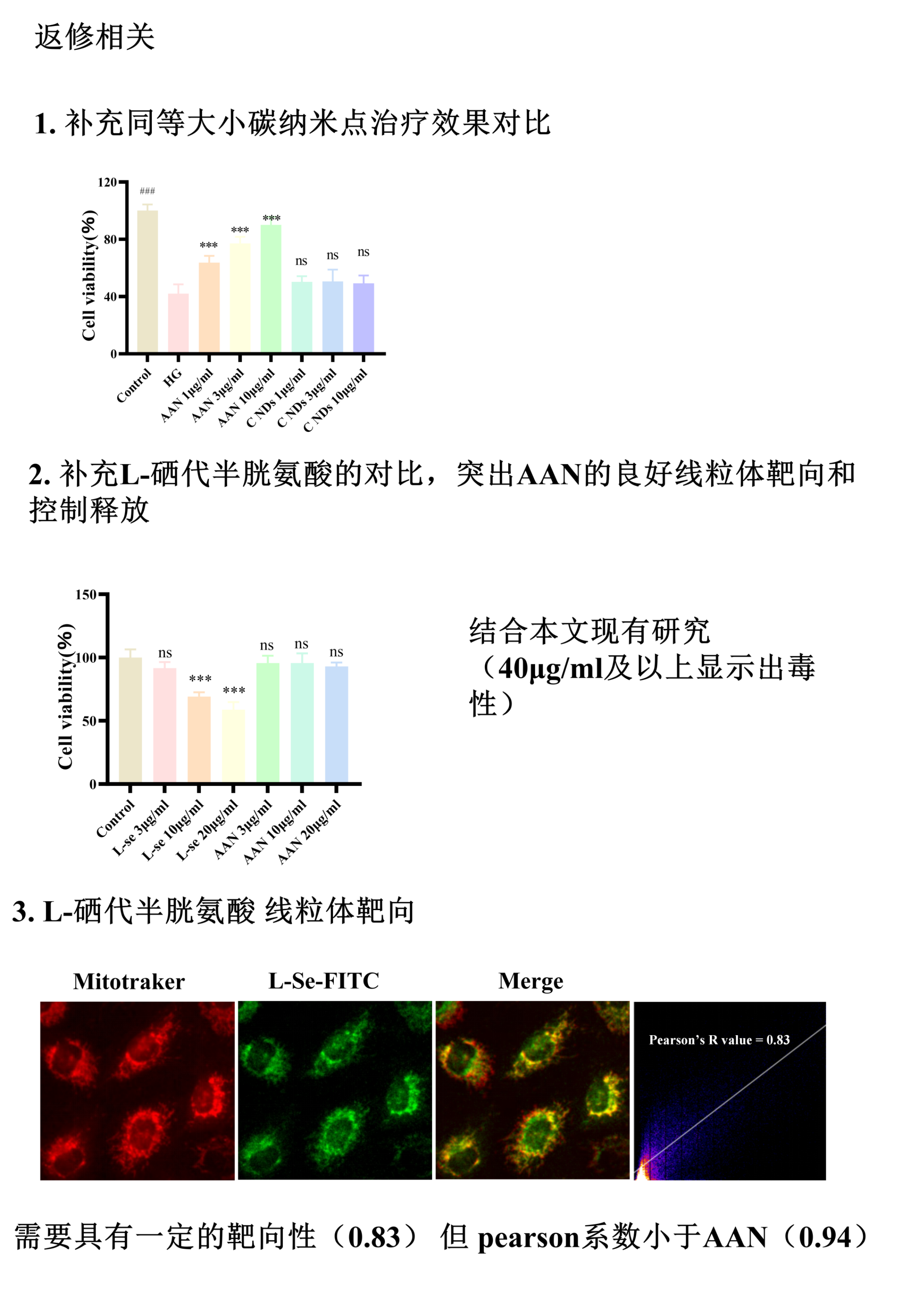


**Figure S19**. Cell viability of HK-2 cells treatment with different concentrations of AAN and L-selenocysteine. ****P* < 0.001 vs Control group, ns means *P* > 0.05.


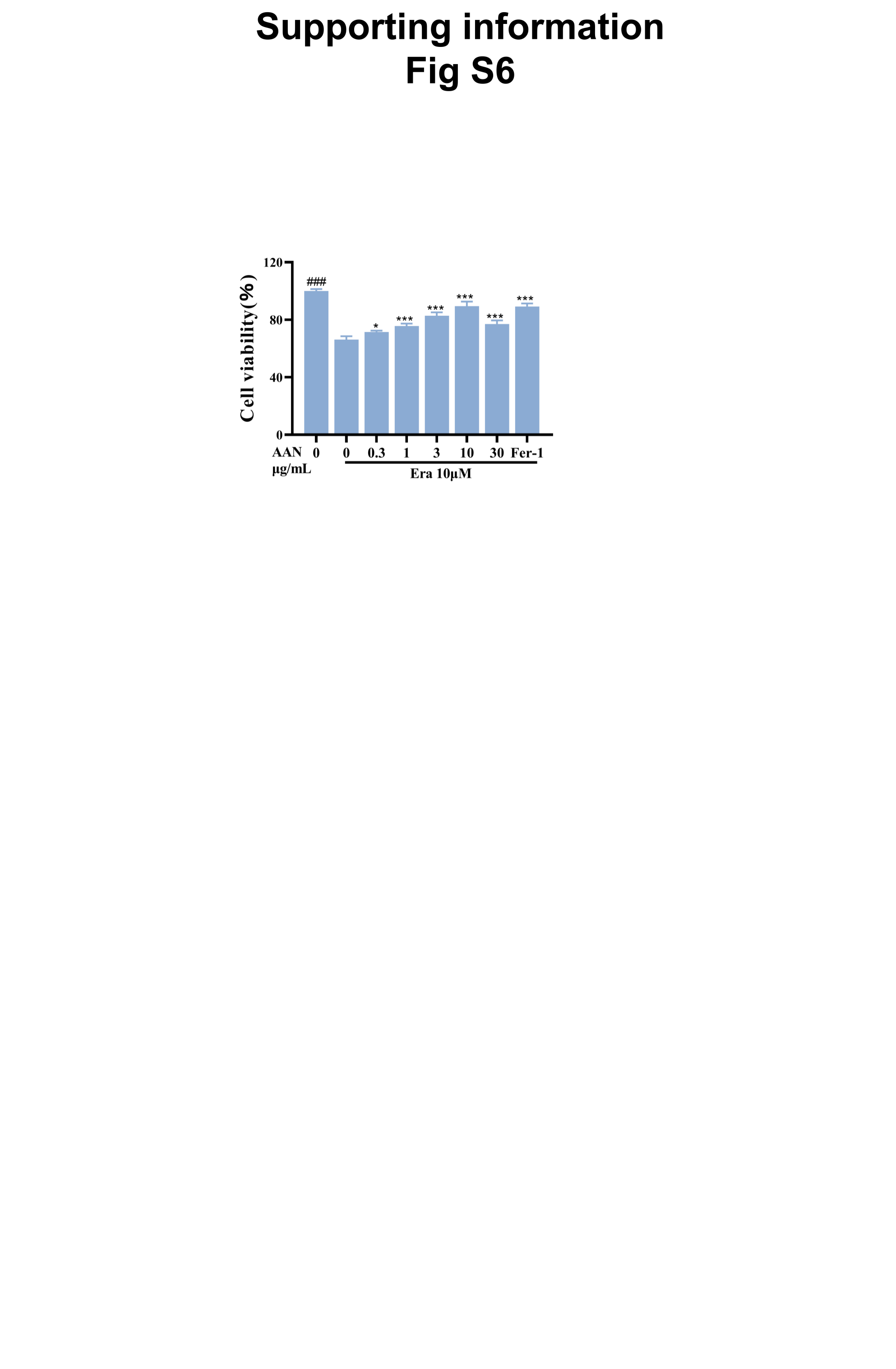


**Figure S20.** Cell viability of HK-2 cells induced by Erastin after treatment with different concentrations of AAN and positive control Fer-1. *^###^P* < 0.001 vs Control group, **P* < 0.05, ****P* < 0.001 vs Model group.


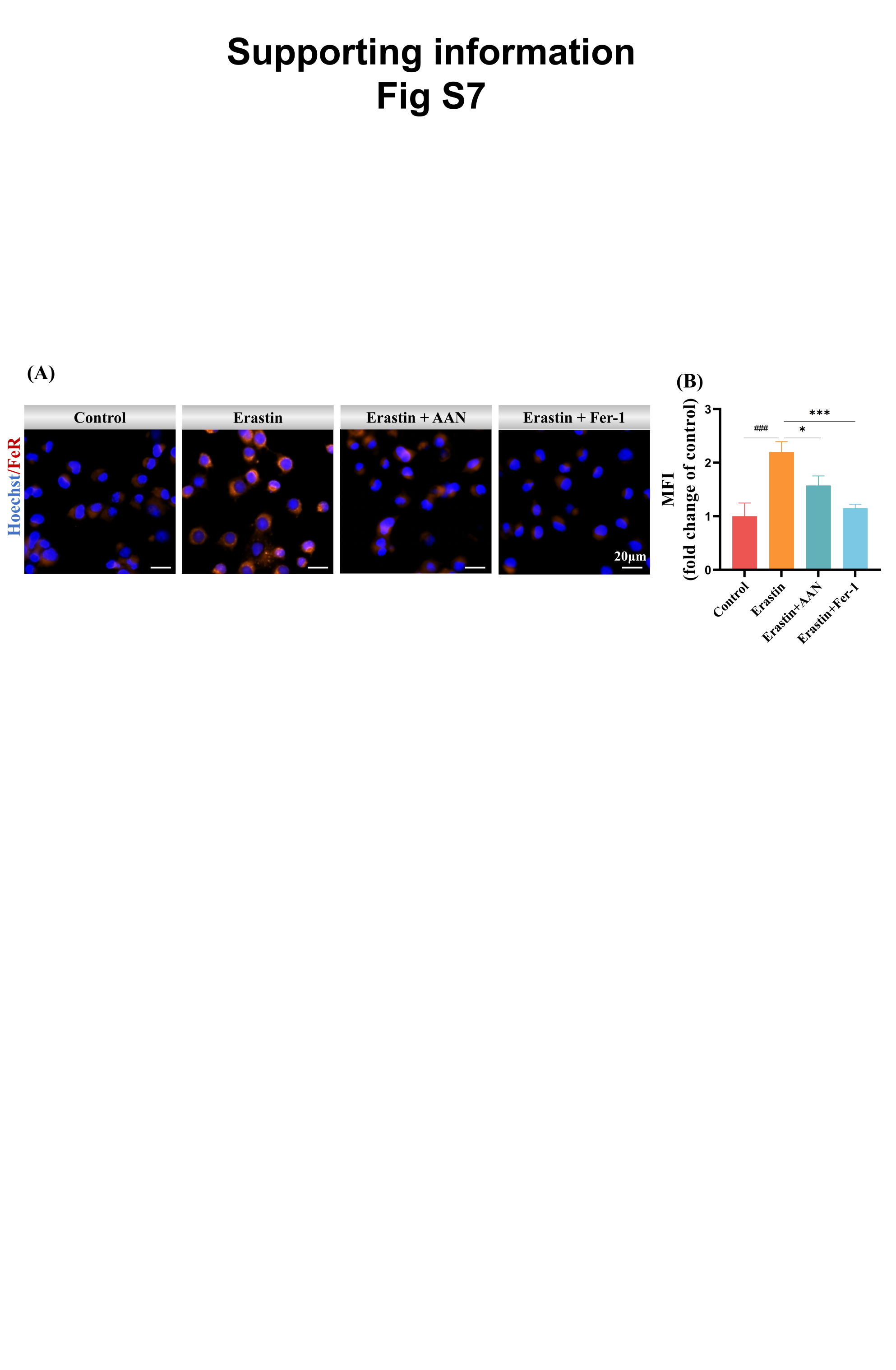


**Figure S21.** Representative images of FeRhoNox-1 (FeR)-staining in Erastin-induced HK-2 cell treated with AAN and positive Control Fer-1 (A), and quantitative analysis (B), Scale bar: 20μm. *^###^P* < 0.01 vs Control group, **P* < 0.05, ****P* < 0.001 vs Erastin group.


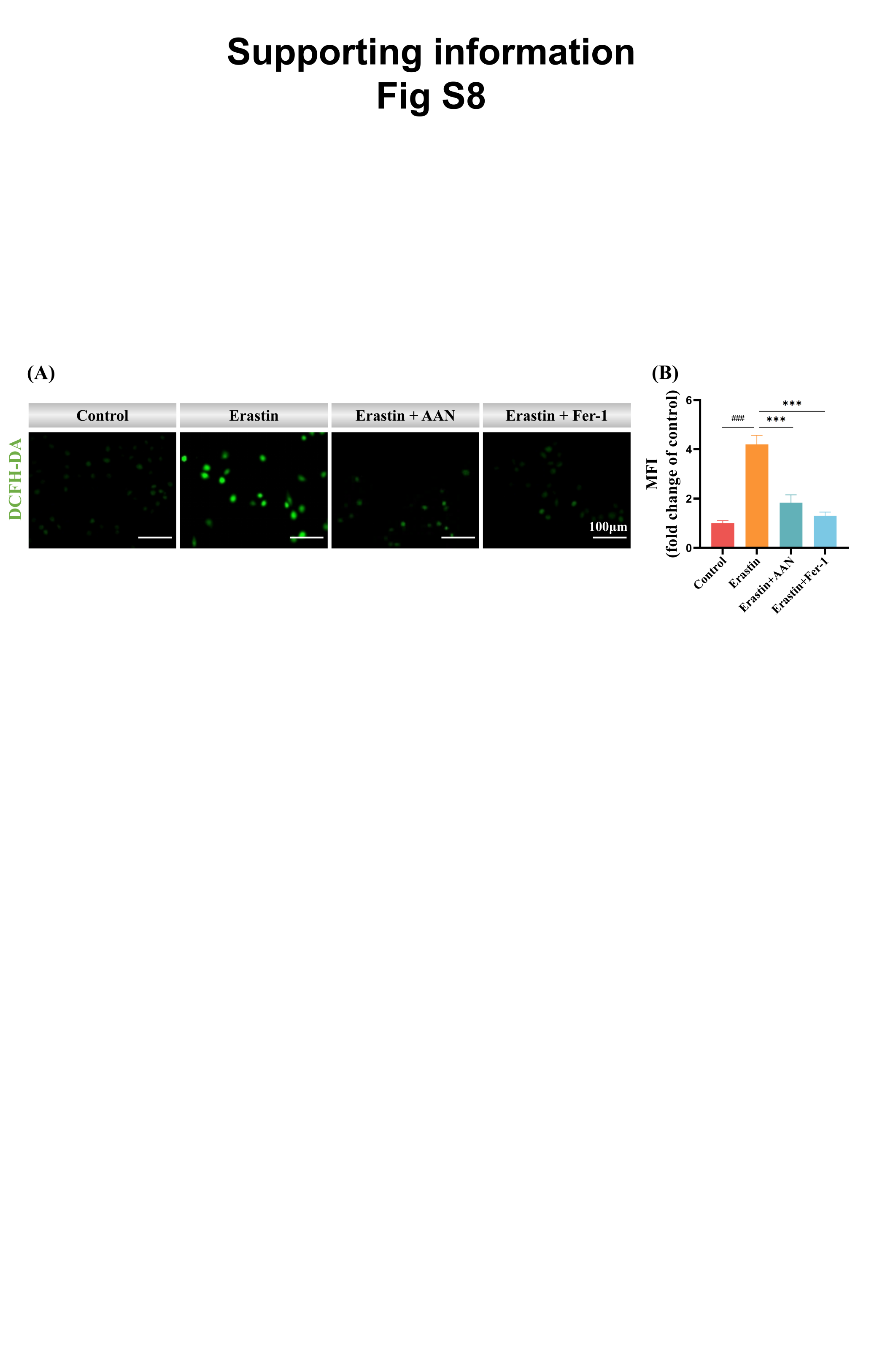


**Figure S22.** Representative images of DCFH-DA-staining in Erastin-induced HK-2 cell treated with AAN and positive Control Fer-1 (A), and quantitative analysis (B), Scale bar: 100μm. *^###^P* < 0.001 vs Control group, ****P* < 0.001 vs Erastin group.


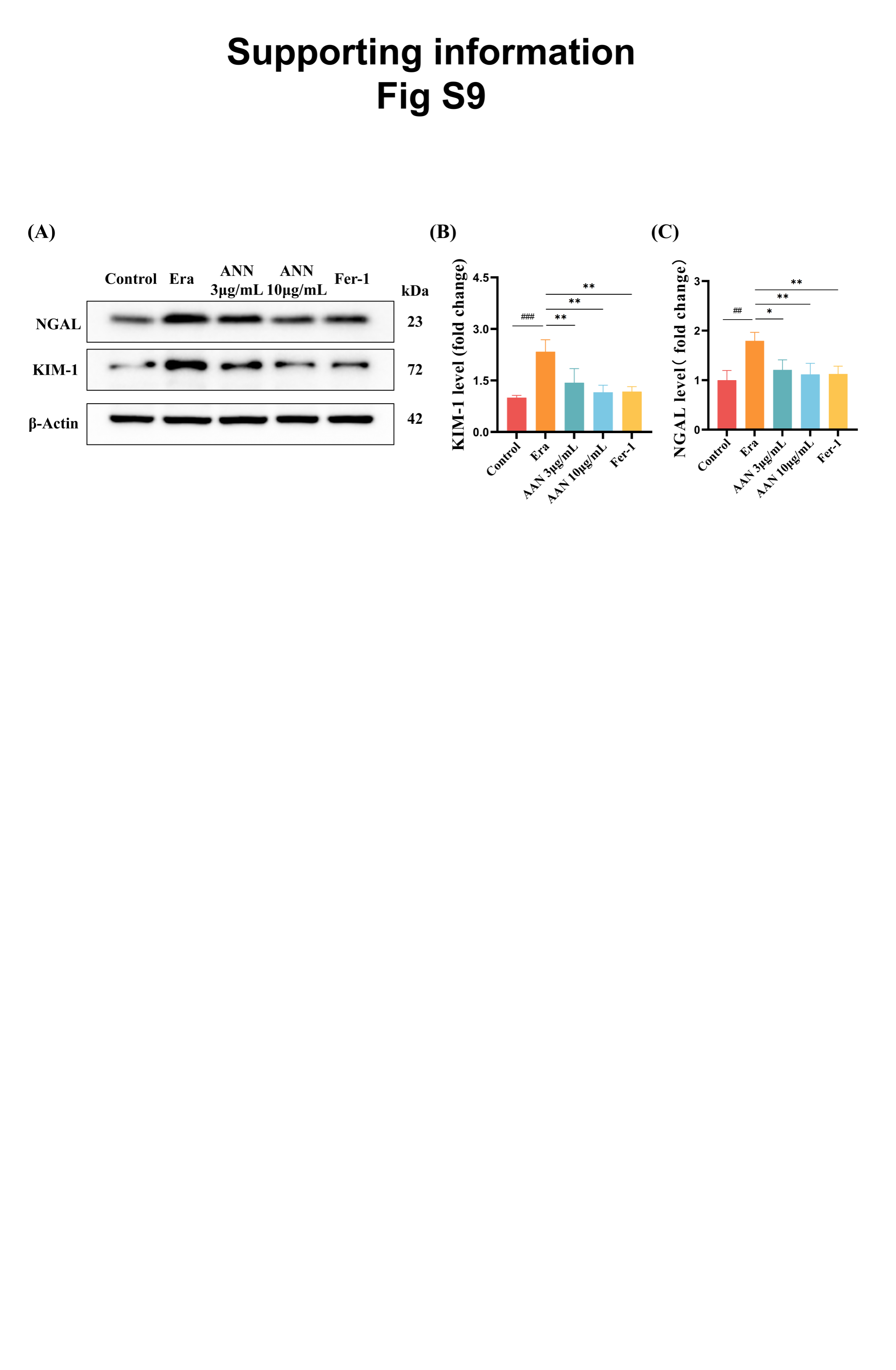


**Figure S23.** Western blot analysis of kidney injury-related protein expression levels in Erastin-induced HK-2 cells treated with different concentrations of AAN and positive Control Fer-1 (A) and quantitative analysis of KIM-1 (B), NGAL (C). *^###^P* < 0.001, *^##^P* < 0.01 vs Control group, **P* < 0.05, ***P* < 0.01 vs Erastin group.


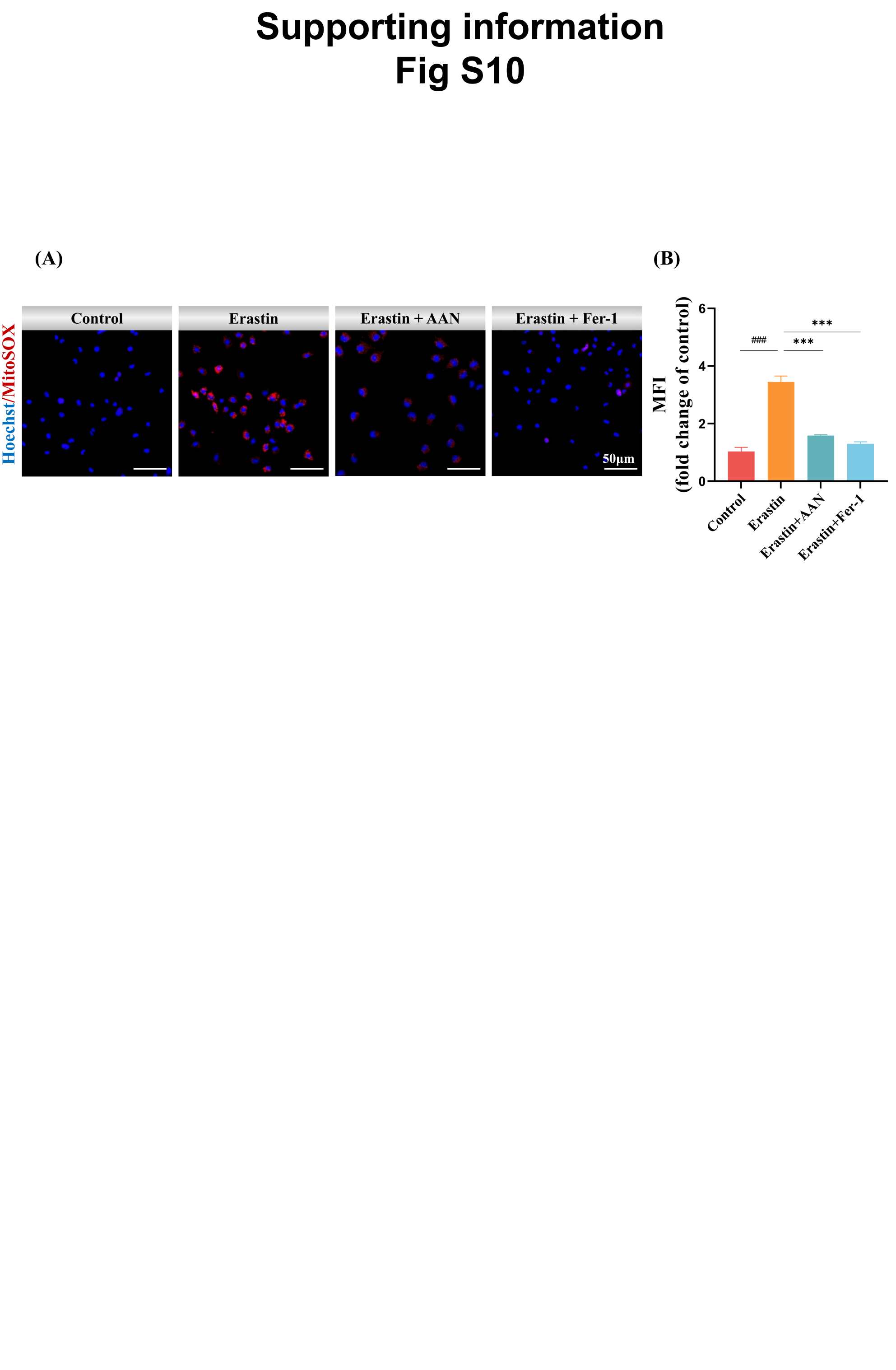


**Figure S24.** Representative images of MitoSox-staining in Erastin-induced HK-2 cell treated with AAN and positive Control Fer-1 (A), and quantitative analysis (B), Scale bar: 50μm. *^###^P* < 0.001 vs Control group, ****P* < 0.001 vs Erastin group.


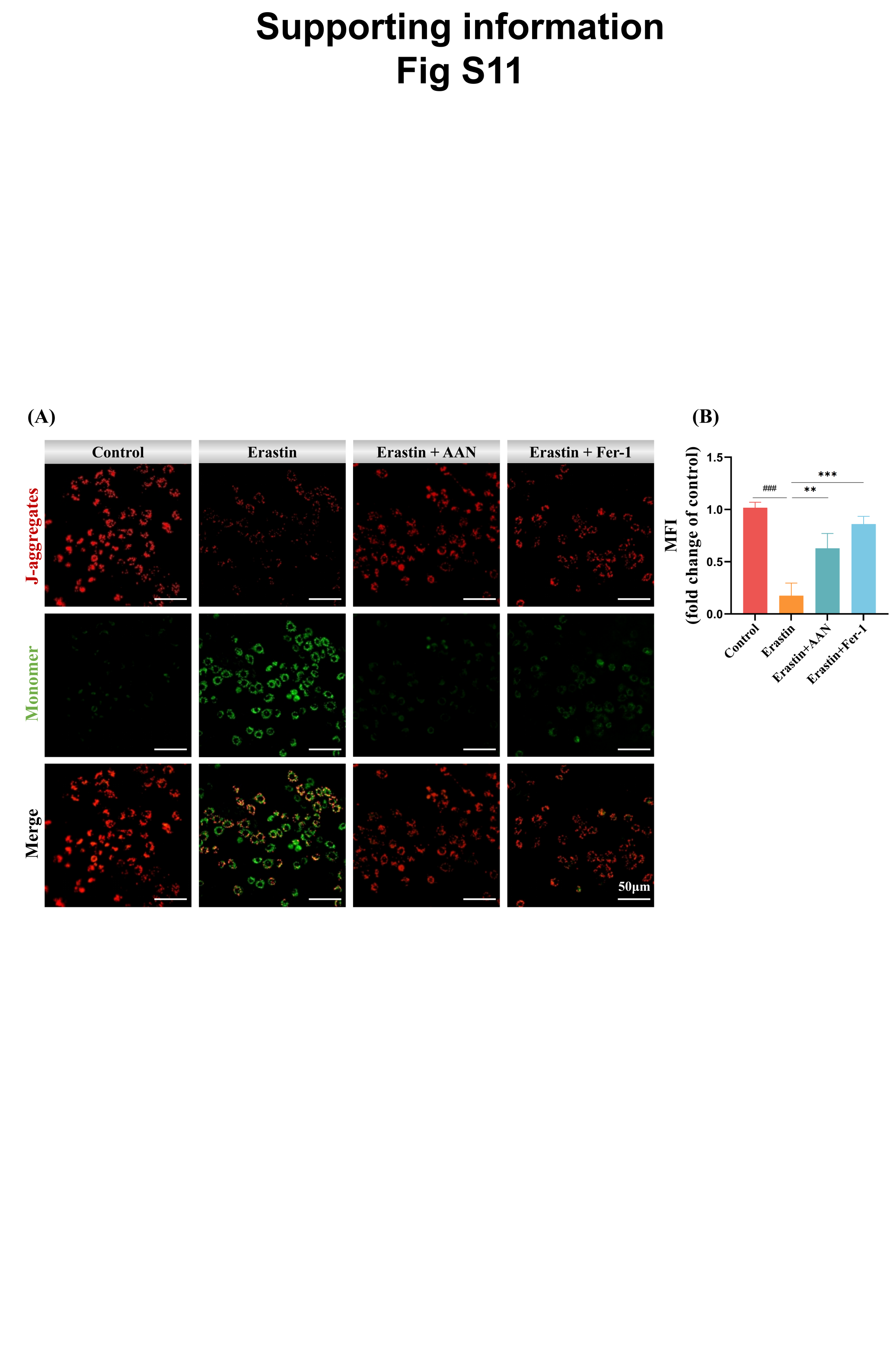


**Figure S25.** Representative images of JC-1-staining in Erastin-induced HK-2 cell treated with AAN and positive Control Fer-1 (A), and quantitative analysis (B), Scale bar: 50μm. *^###^P* < 0.001 vs Control group, ***P* < 0.001, ****P* < 0.001 vs Erastin group.


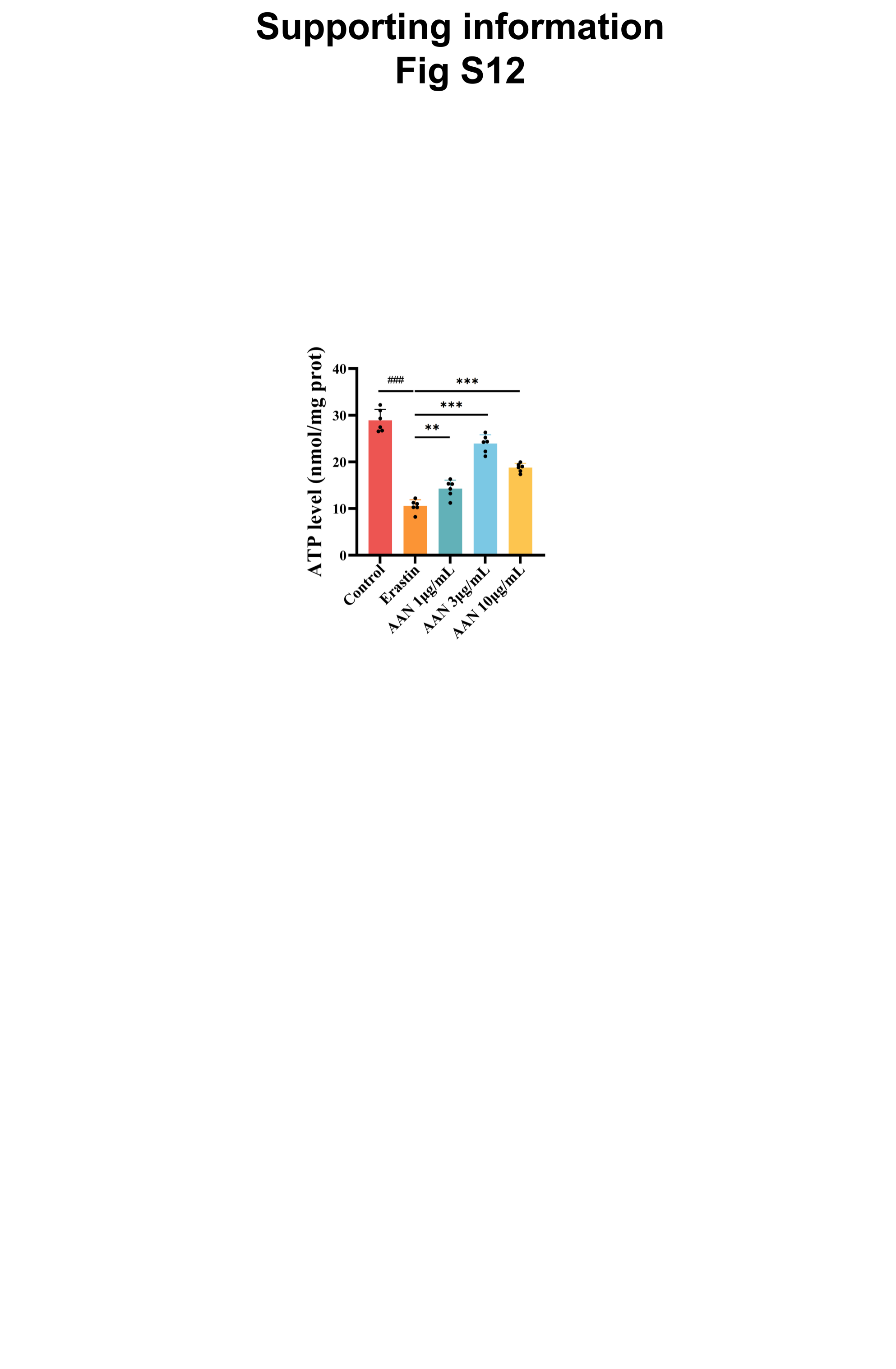


**Figure S26.** ATP level of Erastin-induced HK-2 cells treated with the different concentrations of AAN. *^###^P* < 0.001 vs Control group, ***P* < 0.001, ****P* < 0.001 vs Erastin group.


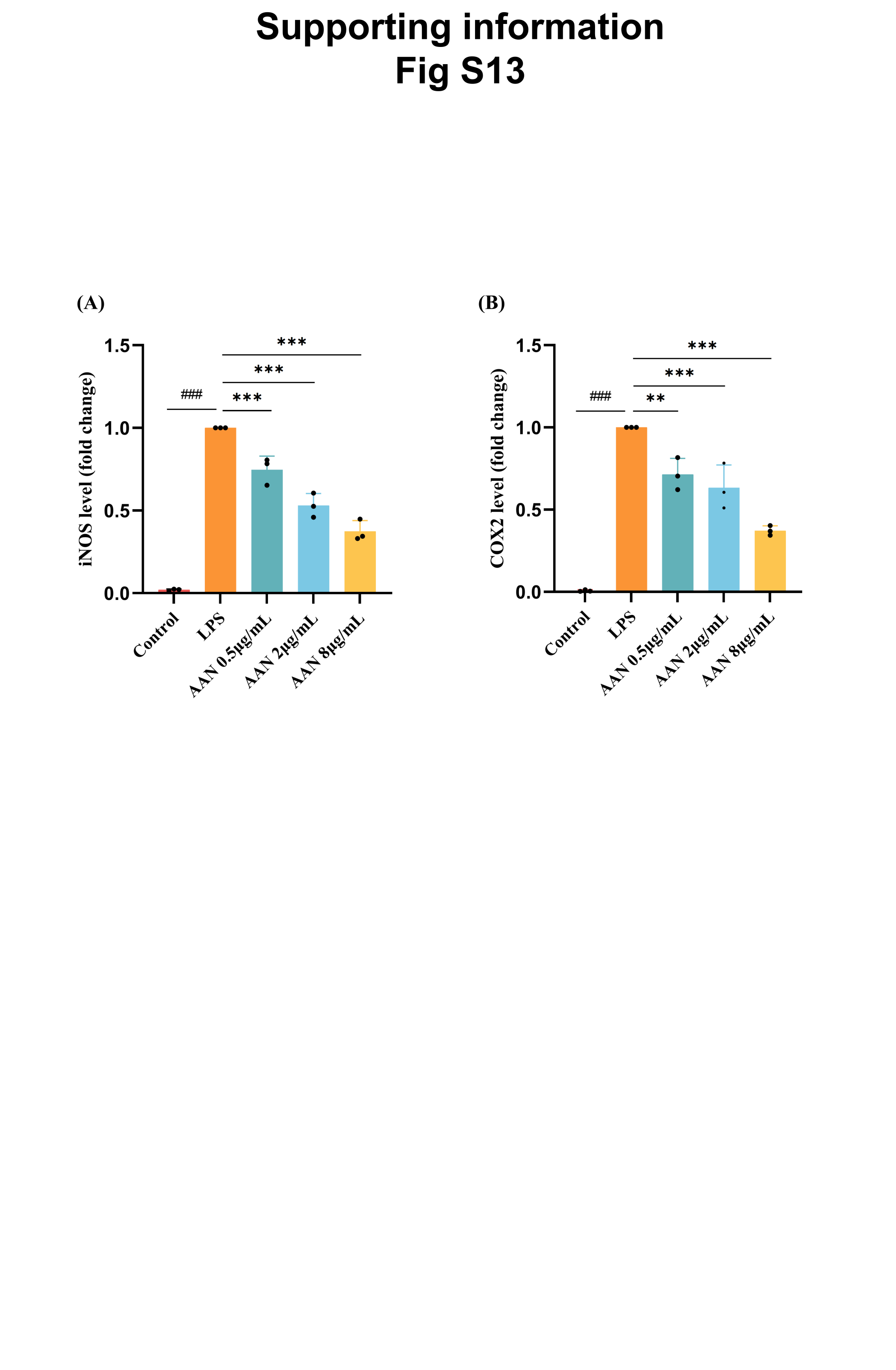


**Figure S27.** Western blot analysis of Infammation-related protein expression levels in LPS-induced RAW 264.7 cells treated with different concentrations of AAN (A) and quantitative analysis of iNOS (B), COX-2 (C). *^###^P* < 0.001 vs Control group, ***P* < 0.001, ****P* < 0.001 vs LPS group.


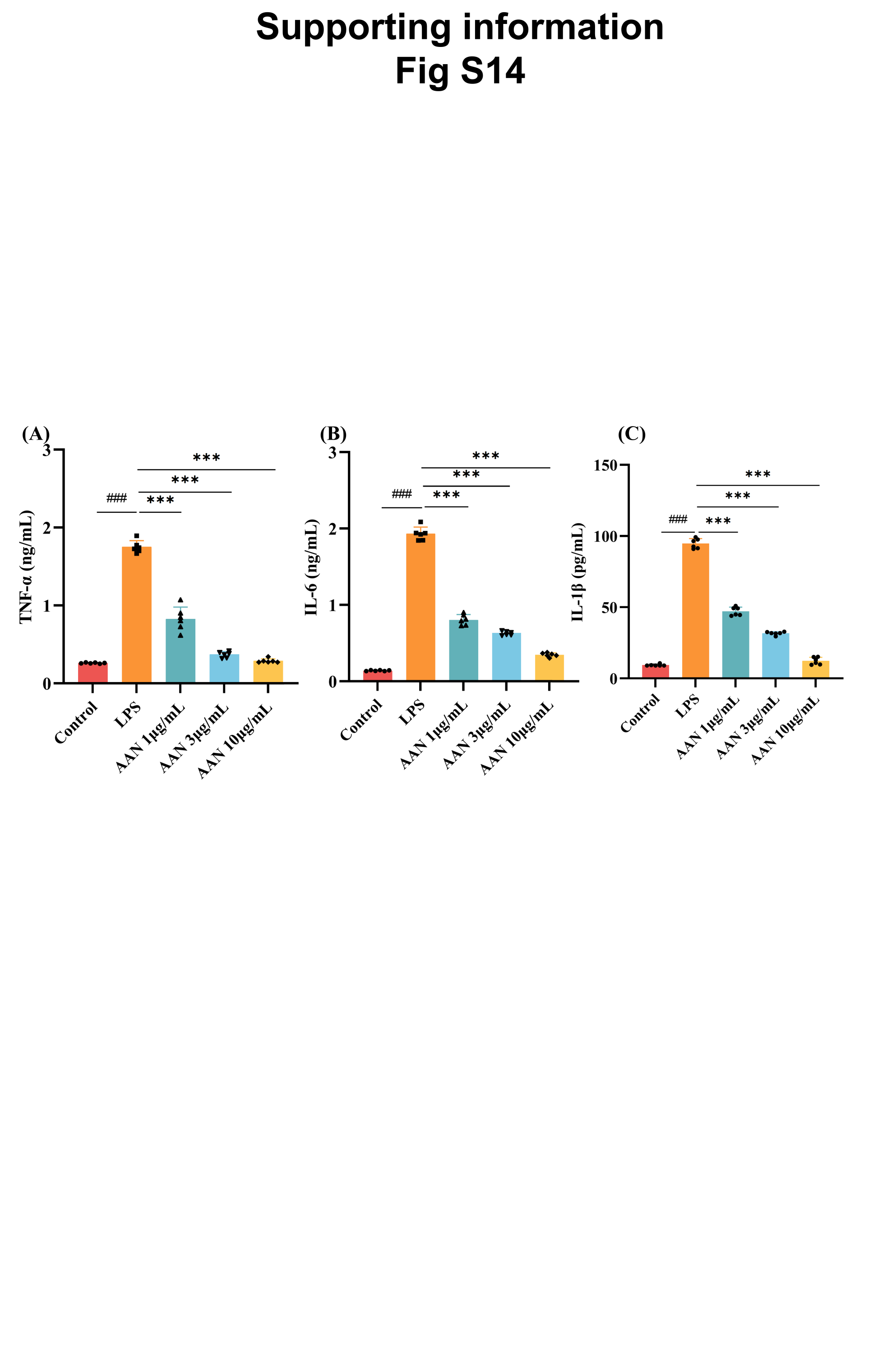


**Figure S28**. Enzyme-linked immunosorbent assays of TNF-α (A), IL-6 (B) and IL-1β (C) in LPS-induced RAW 264.7 cells treated with different concentrations of AAN. *^###^P* < 0.001 vs Control group, ****P* < 0.001 vs LPS group.


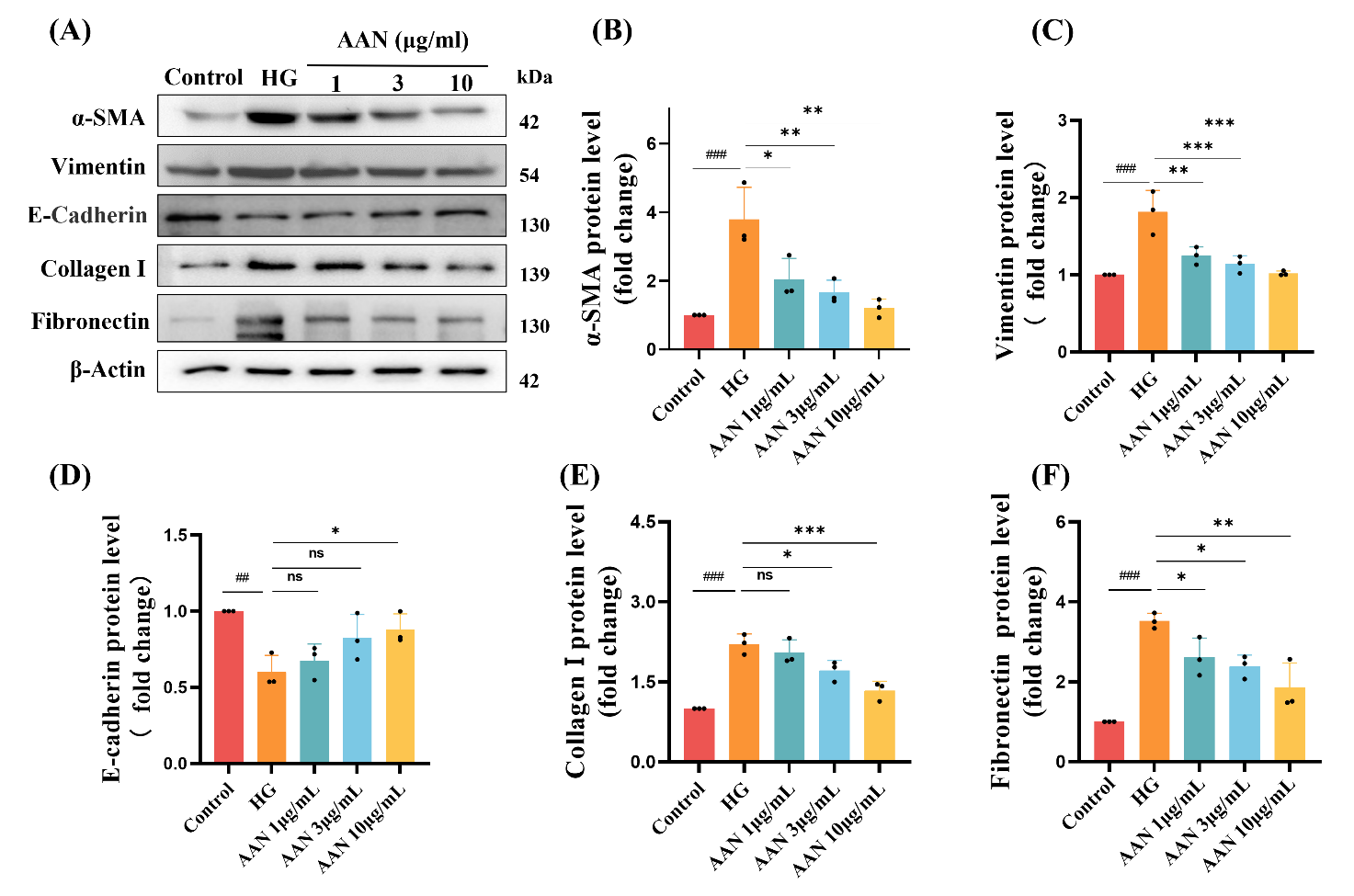


**Figure S29.** Western blot analysis of fibrosis-related protein expression levels in high glucose-induced HK-2 cells treated with different concentrations of AAN (A), and quantitative analysis of α-SMA (B), vimentin (C), E-cadherin (D), Collagen I (E) and Fibronectin (F). *^##^P* < 0.01, *^###^P* < 0.001 vs Control group, **P* < 0.05, ***P* < 0.01, ****P* < 0.001 vs HG group.


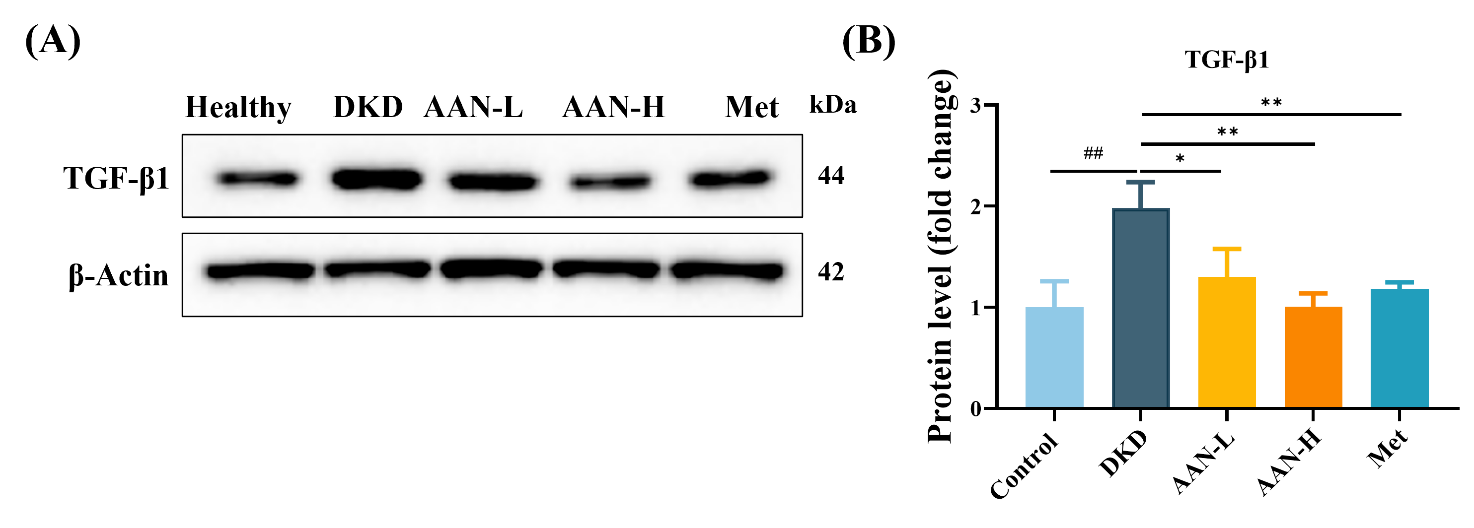


**Figure S30.** Western blot analysis of TGF-β expression levels in mice kidney(A), and quantitative analysis of TGF-β (B). *^##^P* < 0.01 vs Control group, **P* < 0.05, ***P* < 0.01 vs DKD group.


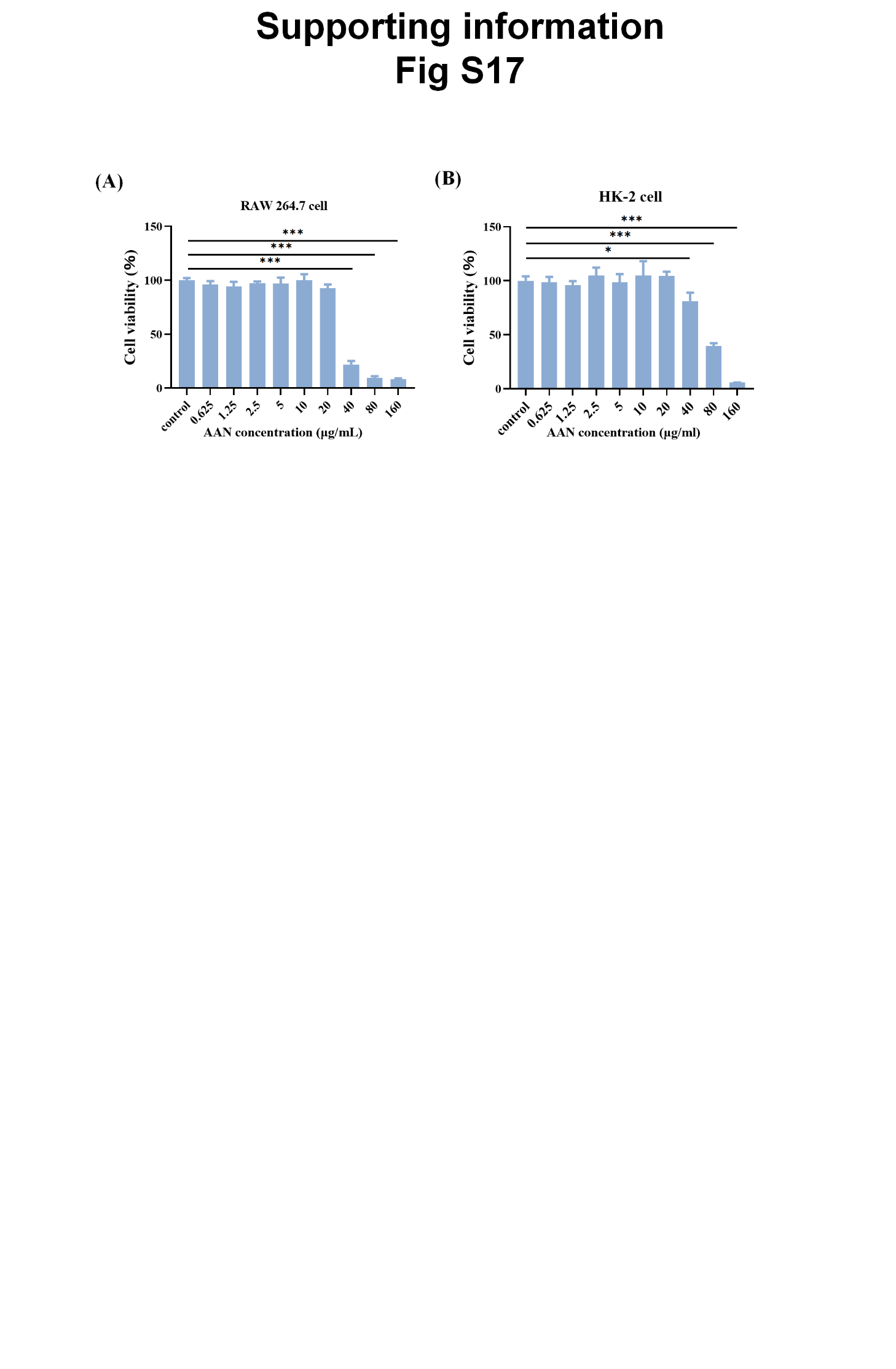


**Figure S31**. The CCK8 results of RAW264.7 cells and HK-2 cells when incubated at different concentrations. **P* < 0.05, ****P* < 0.001 vs Control group.

**
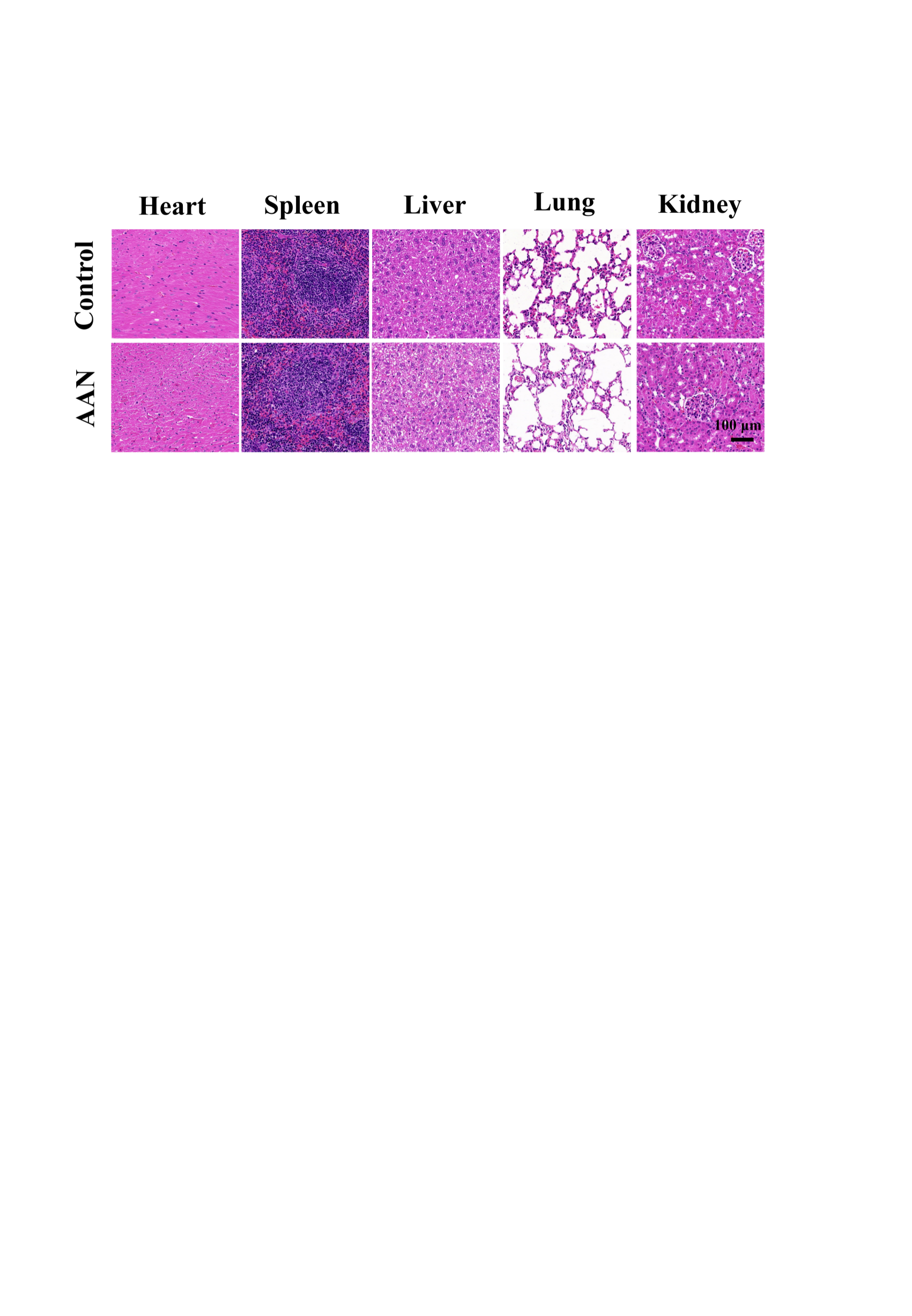
**

**Figure S32.** HE staining of tissues at 12 weeks, Scale bar: 50 μm.


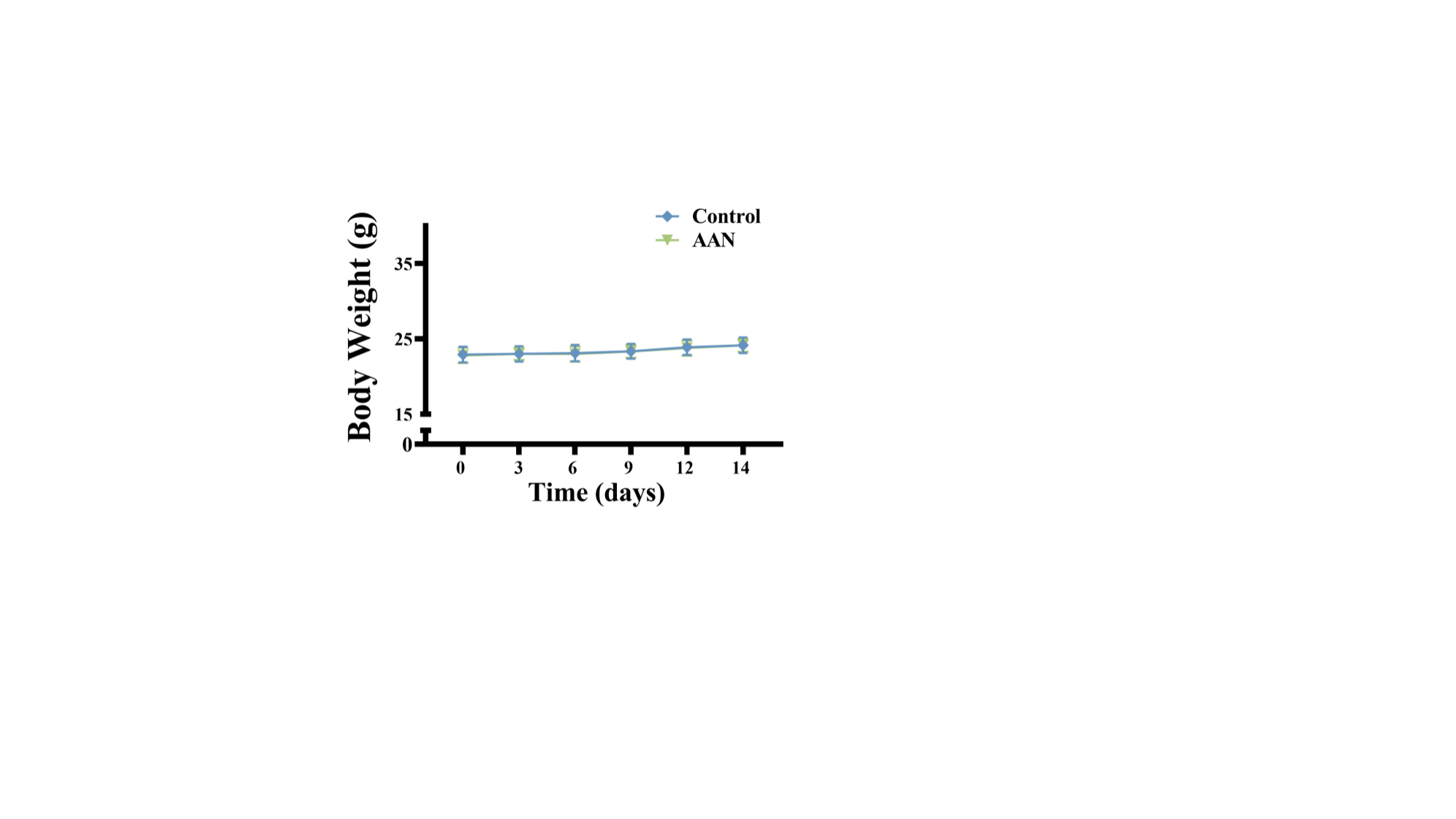


**Figure S33.** Continuous administration of AAN, body weight in the first 14 days.
